# Dispersion‐Clamped Crystalline Supramolecular Columnar π‐Stacks of Adamantyl‐Substituted Pyrenes

**DOI:** 10.1002/anie.1681939

**Published:** 2026-08-22

**Authors:** Kazutaka Shoyama

**Affiliations:** ^1^ Institut für Organische Chemie Universität Würzburg Würzburg Germany; ^2^ Center for Nanosystems Chemistry (CNC) Universität Würzburg Würzburg Germany

**Keywords:** crystalline phases, Frontier crystal orbitals, supramolecular chemistry, zero‐offset π‐stacks, π‐interactions

## Abstract

Despite their importance in supramolecular chemistry, zero‐offset columnar π‐stacks of polycyclic aromatic hydrocarbons (PAHs) are rarely observed in crystals because close cofacial packing is penalized by Pauli repulsion. Here we show a crystalline phase (CP) of adamantyl‐substituted pyrene that exhibits one‐dimensional, translationally aligned columnar π‐stacks enabled by dispersion‐assisted adamantyl “clamps”. Single‐crystal x‐ray diffraction reveals an exceptionally short interplanar separation of 3.297(3) Å at 100 K. Energy‐decomposition analysis shows that the adamantyl substituents provide larger dispersion stabilization relative to their Pauli penalty, rationalizing the formation of the zero‐offset π‐columns. The solution‐to‐crystal color shift is exceptionally large (Δ*E*_obs_ = −0.761 eV), which exceeds exciton coupling contributions and is dominated by intermolecular orbital interactions captured by a frontier crystal orbital (FCO) model. To the best of our knowledge, both the translationally zero‐offset π‐columns and the large crystallization‐induced red shift are unprecedented for ambient‐pressure CPs of neutral, closed‐shell hydrocarbon PAHs.

## Graphical abstract

Can neutral hydrocarbon π‐systems be forced into perfectly aligned cofacial stacks in crystals? An adamantyl‐decorated pyrene derivative crystallizes into rare translationally zero‐offset π‐columns, producing a short π–π distance below 3.3 Å and a large color shift arising from frontier crystal orbital (FCO) formation.

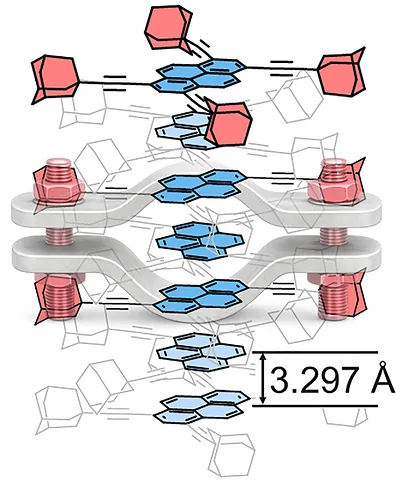

Although polymeric columnar π‐stacks (zero‐offset π‐stacks) of polycyclic aromatic hydrocarbons (PAHs) are frequently invoked in supramolecular and functional materials research, they are in reality rarely observed in crystal structures (Figure 1) [1, 2, 3, 4, 5, 6, 7, 8, 9, 10, 11, 12, 13]. Instead, slipped π‐stacks or herringbone packing dominate the intermolecular interactions of PAHs (Figure 1a) [14, 15]. This scarcity of translationally zero‐offset, columnar π‐stacks reflects the steep short‐range Pauli repulsion of perfectly cofacial contacts, whereas slipped π‐stacks can retain strong dispersion stabilization at reduced repulsion in apolar PAH crystals (Figure 1b,c) [16, 17, 18, 19, 20]. Limited cases in which columnar stacks are observed typically involve strongly directing interactions, such as pronounced electrostatics [21, 22], steric constraints (e.g. discotic liquid crystals) [23, 24, 25], or metal coordination [26, 27]. However, pristine PAHs with dispersion‐dominated attraction have so far rarely yielded translationally aligned π‐columns. We hypothesized that large dispersion interactions introduced by diamondoids such as adamantane [28] can orient apolar PAHs into zero‐offset π‐stacks.

**FIGURE 1:**
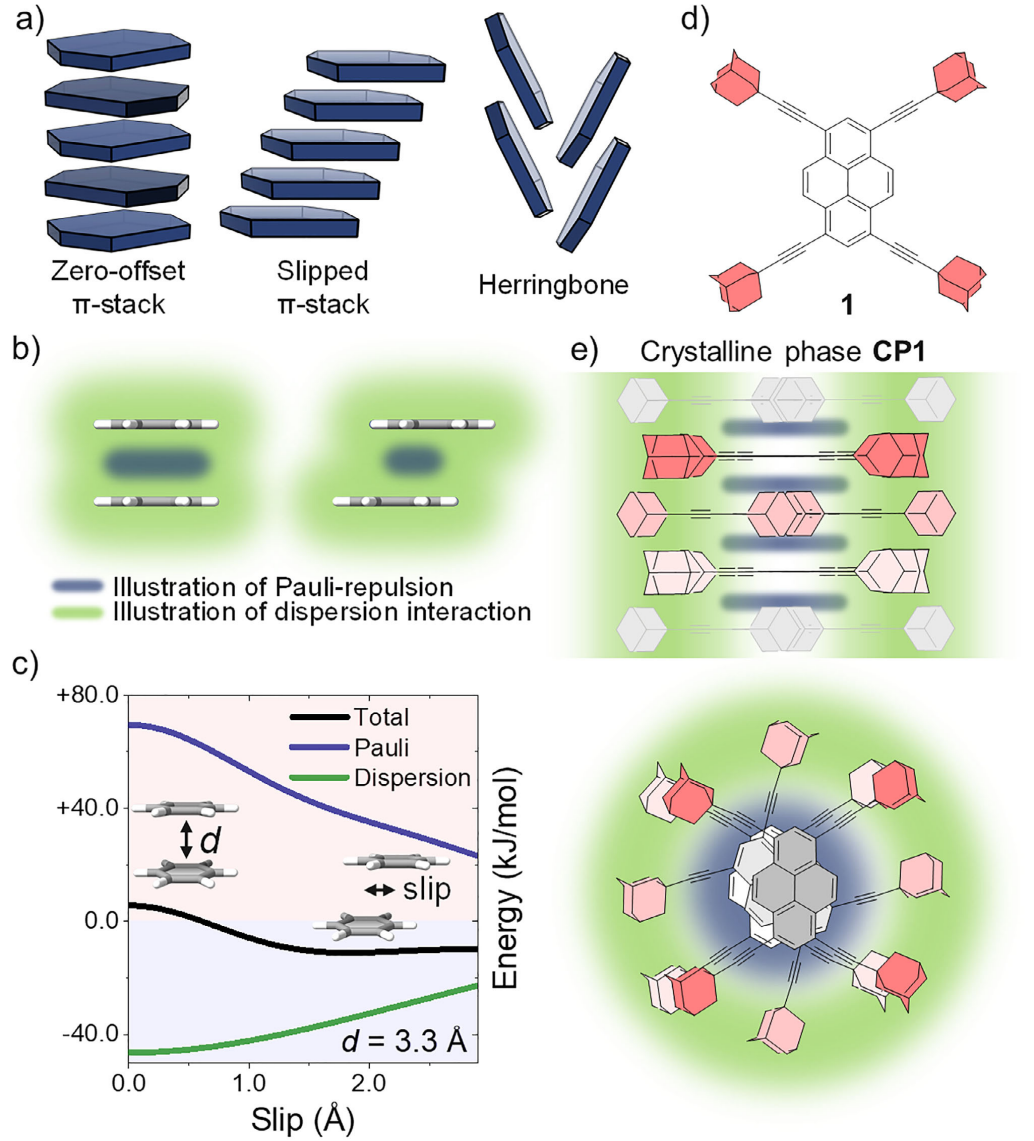
Design concept for zero‐offset π‐stacks of PAH. (a) Schematic packing motifs of PAHs: zero‐offset π‐stacks (target), slipped π‐stacks, and herringbone packing. (b) Schematic illustration of short‐range Pauli repulsion (blue) versus dispersion attraction (green). (c) Interaction‐energy landscape showing the contributions of the total interaction (black), Pauli repulsion (blue), and dispersion (green) as a function of in‐plane slip. The total contains additionally electrostatic and induction contributions. Data were adapted from ref. [20]. (d) Chemical structure of **1**. (e) Translationally zero‐offset π‐stacks observed in **CP1**.

Here we show translationally zero‐offset π‐stacks in a crystalline phase (CP) of pyrene derivative **1**, **CP1** = [**1**·(*n*‐hexane)] (Figure 1d,e), enabled by noncovalent, dispersion‐assisted “clamps” composed of four adamantylethynyl substituents. In **CP1**, the pyrene cores stack orthogonal to the π‐plane and are laterally fixed by the peripheral adamantyl groups through close, dispersion‐dominated contacts (Figure 1e). The resulting columnar π‐stacks display an unusually short interlayer π–π distance of 3.297(3) Å at 100 K, which enables strong intermolecular orbital interactions and results in a large bathochromic solution‐to‐crystal shift of −0.761 eV (colorless solution to orange crystals). The formation of the π‐columns gives rise to channel‐like voids sustained by heavily disordered *n*‐hexane solvate molecules. To the best of our knowledge, both the translationally zero‐offset π‐stack and a crystallization‐induced red shift as large as that observed for **CP1** are unprecedented for ambient‐pressure CPs of neutral, closed‐shell PAHs composed only of carbon and hydrogen. Together, these results underscore that introducing adamantyl groups to a PAH core can provide additional dispersion stabilization sufficient to steer packing away from the slipped or herringbone motifs commonly favored by the balance of Pauli repulsion and dispersion in PAH CPs. Moreover, the unusually large spectral shift of **CP1** relative to isolated **1** provides a design principle for functional organic materials, in which precise control of intermolecular interactions in the solid state is critical for tuning optoelectronic properties. We anticipate that controlling packing through the dispersion forces of purely hydrocarbon adamantyl groups will open an avenue toward PAH CPs with tailor‐made optoelectronic properties.

Crystallization of a colorless dilute dichloromethane solution of **1** [29] (*c* < 10^−4^ M) yielded orange needle‐shaped crystals of **CP1** [30] as an *n*‐hexane solvated monoclinic phase [**1**·(*n*‐hexane)], which provided unambiguous evidence for translationally zero‐offset π‐stacking (Figure 2). This new CP of **1**, **CP1**, is distinct from the previously reported pristine triclinic CP of **1**, **CP2** = [**1**] [29], which forms preferentially under more concentrated conditions (*c* ≥ 10^−4^ M) and adopts slipped π‐stacking (Table S1 and Figure S3). The preferential formation of **CP1** under dilute conditions is consistent with the incorporation of *n*‐hexane into channel‐like voids that are a geometric consequence of the zero‐offset packing motif, whereas the more densely packed slipped‐stack phase **CP2** does not contain solvent‐accessible voids (Figure S3). In the monoclinic lattice (*a–c* ⟂ *b* per se) of **CP1**, the pyrene π‐plane is aligned with the crystallographic *a–c* plane, while the center of each molecule lies on an inversion center coinciding with the crystallographic twofold axis (Figure S5). Because this axis runs along the *b* direction and is exactly orthogonal to the π‐surface, stacking occurs exactly along the π‐plane normal, producing one‐dimensional (1D) columns with perfect translational registry (Figure 2a,b). In this packing motif, the four peripheral adamantyl substituents are alternately interdigitated along the column and assemble into a thick, wall‐like ring that laterally encases each π‐stacking column, effectively providing a round, sterically stabilized separation from all neighboring columns (Figure 2a,b).

**FIGURE 2:**
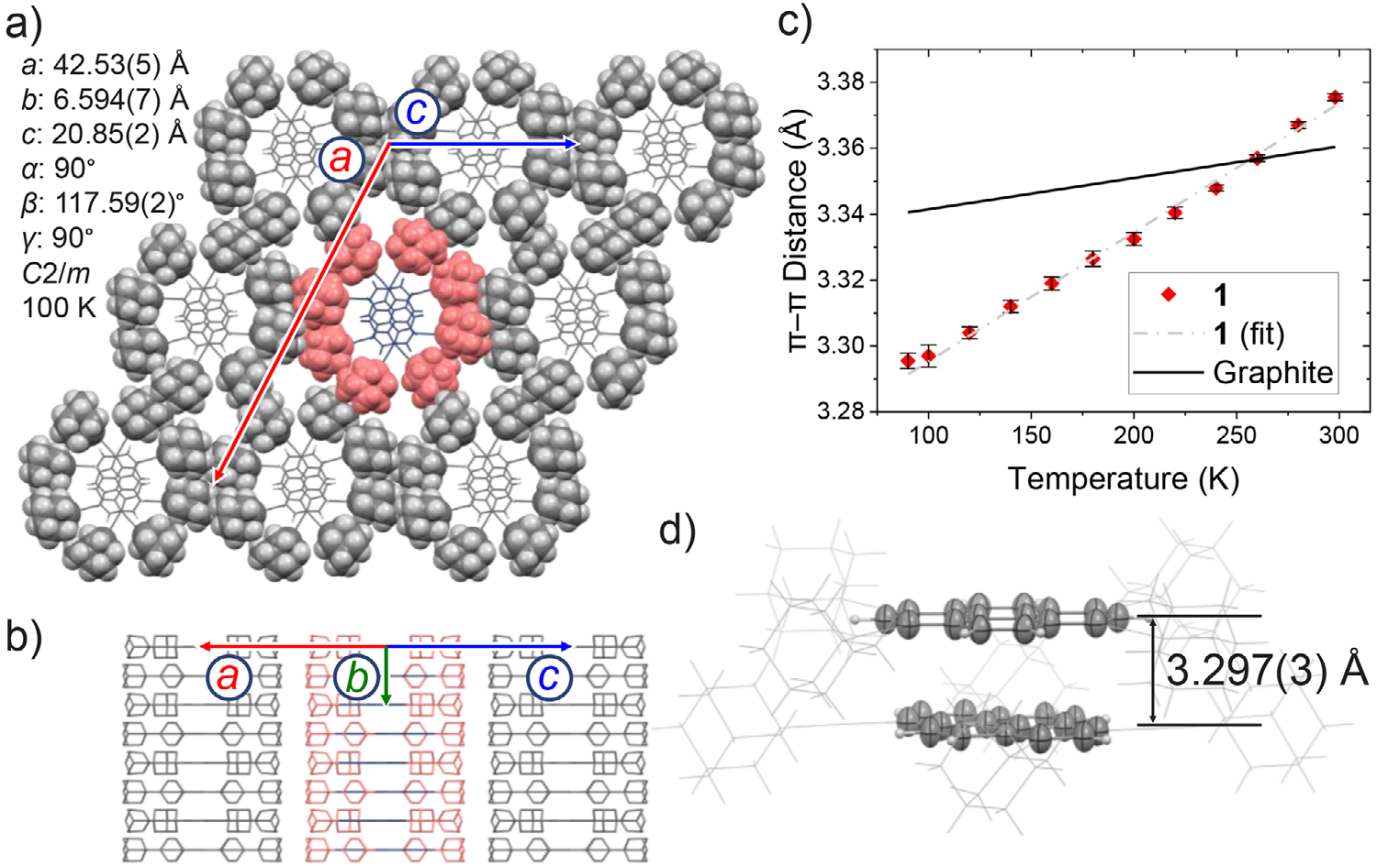
Crystal structure of **CP1**. (a) Top (view down the *b*‐axis, onto the *a–c* plane) view of the one‐dimensional columns of π‐stacked **1**. One translationally aligned π‐stacked column is highlighted by color. (b) Side‐view of the columns in Figure 2a. (c) Temperature dependence of the interplanar π–π distance. Red symbols: experimental values for **CP1**; dashed line: linear fit; solid line: graphite reference (002) spacing [31]. (d) A π‐stacking dimer from the polymeric column (3.297(3) Å at 100 K, corresponding to *b*/2). Ellipsoids drawn at 30% probability; solvating *n*‐hexane residues omitted for clarity.

An exceptionally short interplanar π–π separation was observed for the columnar stacks in **CP1**. Because the unit cell *b*‐axis spans two successive π‐layers within a given column, the single‐layer separation is *b*/2, giving *d*_π–π_ = 3.297 ± 0.003 Å at 100 K (from *b* = 6.594 ± 0.0067 Å). Interplanar π–π spacings below 3.3 Å are rare and are typically encountered only when additional structure‐directing factors or unusual electronic states enforce unusually tight packing (e.g. radical columns [32], donor–acceptor architectures [22, 33], metal‐coordinated stacks [26], or lamellar motifs [15]), whereas neutral closed‐shell PAHs more commonly adopt slipped or herringbone arrangements with longer separations. Notably, this π–π distance is also shorter than the interlayer spacing of graphite at the same temperature (≈ 3.342 Å [31]). Variable‐temperature crystallographic analysis further shows that the π–π distance in **CP1** increases with a slope of 3.92 × 10^−4^ Å K^−1^, which is about fourfold larger than the corresponding temperature coefficient for graphite (9.52 × 10^−5^ Å K^−1^, [31]) and threefold larger than that for the slipped π–π distance for coronene (1.24 × 10^−4^ Å K^−1^, ref. [34], see Supporting Information). This unusually strong thermal response is consistent with a soft, anharmonic effective potential along the stacking coordinate typical for molecular crystals [35, 36] and motivates a quantitative assessment of how strongly dispersion, particularly from the peripheral adamantyl groups, contributes to stabilizing the columnar contact.

To break down the contribution of adamantyl groups to the intermolecular interactions in **CP1**, we performed density functional theory (DFT) calculations using the absolutely localized molecular orbital energy decomposition analysis (ALMO‐EDA) [37] on a representative stacked bilayer and on fragments separating the pyrene core from the peripheral adamantyl (Ad) groups (Table 1). For the full bilayer, the interaction is dominated by a large attractive dispersion term (*E*_disp_ = −252.6) that competes directly with a large Pauli repulsion (*E*_Pauli_ = +275.6), whereas, as expected from apolar character of **1**, polarization (*E*_pol_ = −3.2) and charge transfer (*E*_CT_ = −36.3) are minor. The electrostatic contribution (*E*_elec_ = −168.2) is a term that generally scales with the overall system size and contact area of the interacting fragments, canceling out a substantial part of the Pauli term. Thus, the key energetic “tug‐of‐war” relevant to the unusually short stacking distance is captured by the balance between dispersion attraction (*E*_disp_, negative value) and the Pauli repulsion (*E*_Pauli_, positive value).

**TABLE 1:** Energy decomposition analysis of the stacking interaction for the bilayer, core‐only, and adamantyl‐only models from crystal structure measured at 100 K.^a^

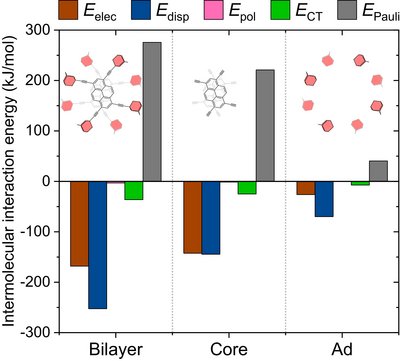						
	*E*_elec_	*E*_disp_	*E*_pol_	*E*_CT_	*E*_Pauli_	*E*_total_
Bilayer	−168.2	−252.6	−3.2	−36.3	+275.6	−184.7
Core	−142.5	−144.4	−1.4	−25.0	+221.0	−92.4
Ad	−26.2	−70.0	−0.1	−7.4	+40.5	−63.2

^a^ Energy decomposition analysis was performed using the ALMO‐EDA at the B3LYP/6‐311G(d) level of theory. Bilayer: extracted dimer from crystal structure of **CP1**; Core: corresponding core‐only dimer obtained by removing the adamantyl groups; Ad: adamantyl fragments alone extracted from the same bilayer geometry. *E*_elec_: permanent electrostatics, *E*_disp_: dispersion, *E*_pol_: polarization, *E*_CT_: charge transfer, and *E*_Pauli_: Pauli repulsion, *E*_total_ = *E*_elec_ + *E*_disp_ + *E*_pol_ + *E*_CT_ + *E*_Pauli_. Energies are reported in kJ mol^−1^; negative values are stabilizing.

Fragment analysis makes this competition transparent (Table 1). For the pyrene core, dispersion (*E*_disp_ = −144.4) cancels only roughly two‐thirds of the corresponding Pauli repulsion (*E*_Pauli_ = +221.0), proportionally lower than the corresponding ratio for the whole dimer (nearly one to one). Strikingly, the peripheral adamantyl groups exhibit outweighed dispersion stabilization of (*E*_disp_ = −70.0) against the Pauli penalty (*E*_Pauli_ = +40.5). This disproportionate dispersion stabilization rationalizes why the hydrocarbon substituents act as effective “clamps” in **CP1**: they provide a dominant attractive component that can offset the Pauli penalty at short range and thereby stabilize a tighter, more strongly dispersion‐governed packing motif than is typical for pristine PAH cores alone. The ca. 45° rotational offset between adjacent layers in **CP1** also mitigates Pauli repulsion, giving a calculated total interaction energy 63.5 kJ mol^−1^ lower than that of a hypothetical bilayer with zero rotational offset (Table S5).

Crystalline phase **CP1** exhibits an unusually large bathochromic solution‐to‐crystal shift, apparent from the color change from a colorless solution to orange crystals (Figure 3a,b; Supporting Information). Using excitation maxima at 100 K in a PMMA matrix as a proxy for absorption of monomers in solution, **1** shows a lowest‐energy absorption band at 432 nm for the monomeric state and at 587 nm for crystalline ensembles on a wafer, corresponding to Δ*E*_obs_ = −0.761 eV (Figure 3a,b, details in Supporting Information). This shift is exceptionally large for apolar organic molecular crystals (typically up to ∼ −0.2 eV; −0.198 eV for **CP2** [29]; ∼−0.293 eV for pentacene [38, 39]). Exciton coupling [5] alone cannot account for this magnitude. Coulombic and charge‐transfer (CT)‐mediated terms (*J*_Coul_ = +0.044 eV, *J*_CT_ = +0.081 eV) predict H‐type behavior for the present geometry, giving a sign‐preserved excitonic contribution $\Delta E_{\mathrm{exci}}^{\mathrm{formal}}$ = +0.075 eV and an upper‐bound red‐shifting excitonic contribution Δ*E*_exci_ = −0.426 eV (also accounting for forbidden transitions, see Supporting Information for details), both substantially smaller in magnitude than Δ*E*_obs_.

**FIGURE 3:**
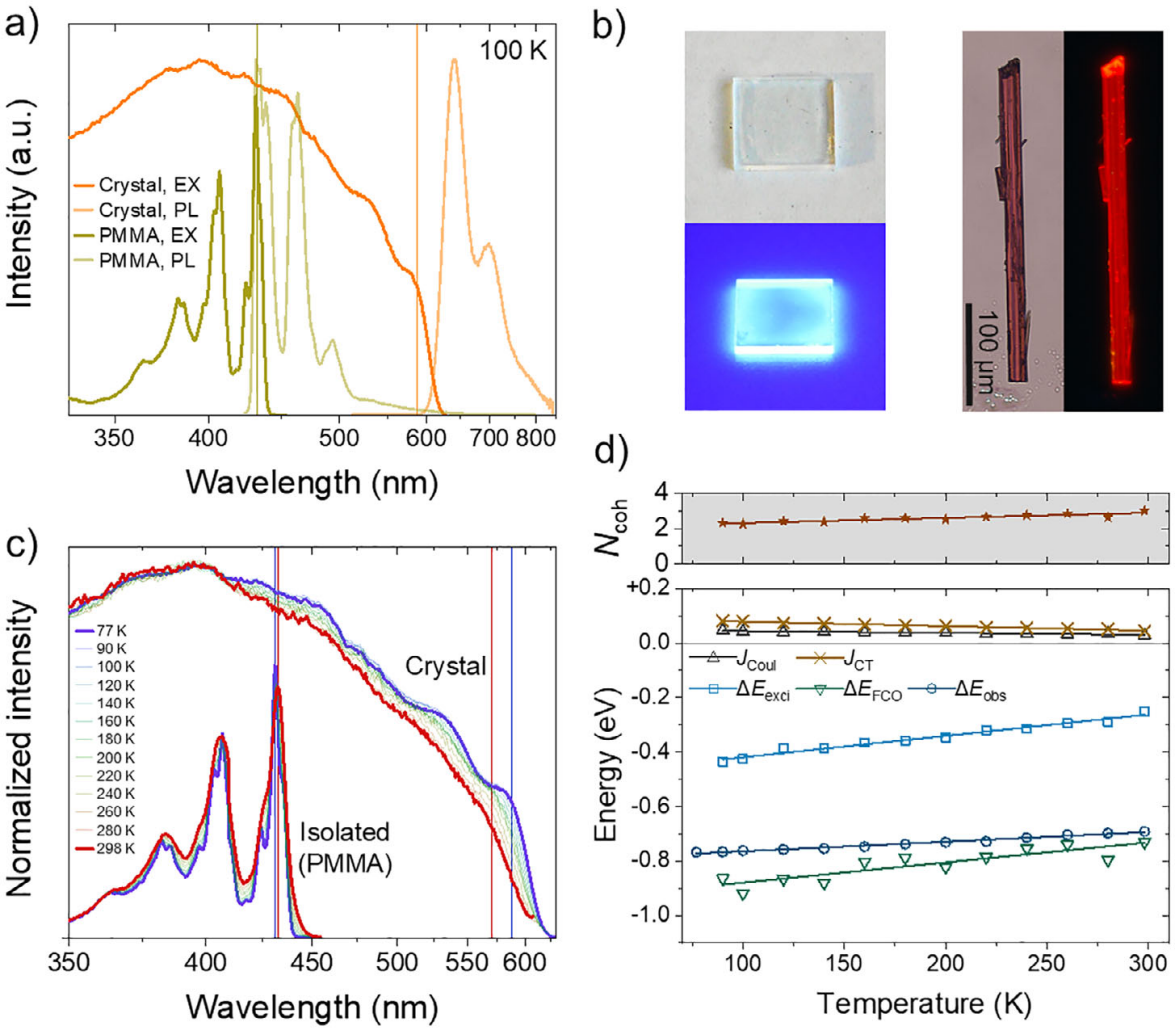
Large bathochromic solution‐to‐crystal shift (Δ*E*_obs_) of **CP1** relative to isolated **1** and its temperature dependence. (a) Excitation spectra of **1** in a PMMA matrix at 100 K compared with ensembles of **CP1** on a wafer. Vertical guide lines mark the excitation maxima at 432 nm (PMMA, yellow) and 587 nm (crystals, orange). (b) Photographs of a PMMA film of **1** (colorless/blue emission) and a crystal of **CP1** (orange/red emission) under ambient light and UV‐light. (c) Variable‐temperature excitation spectra of isolated **1** (PMMA) and **CP1** (77–298 K). Vertical guide lines indicate the band maxima at 77 K (blue) and 298 K (red). (d) Temperature dependence of Δ*E*_obs_, together with the evaluated excitonic contribution Δ*E*_exci_ = −2(|*J*_Coul_| + |*J*_CT_|) + *Δ*_CT_ (*Δ*_CT_: site‐energy correction) and the FCO contribution Δ*E*_FCO_ = 2($E_{g}^{2\mathrm{mer}}$ − $E_{g}^{1\mathrm{mer}}$) ($E_{g}^{X\mathrm{mer}}$: DFT‐calculated HOMO–LUMO gap of *X*mer) calculated using crystal‐structure geometries obtained over 90–298 K. The effective coherence length *N*_coh_ was derived using Δ*E*_obs_ = (Δ*E*_FCO_ + Δ*E*_exci_)cos[π/(*N*_coh_ + 1)]. Solid lines are fits/guides to the eye; see [29] and the Supporting Information for details.

We therefore attribute the dominant contribution to Δ*E*_obs_ to band‐like intermolecular orbital interactions [40, 41]. This band‐like contribution is then interpreted within a finite‐coherence orbital framework, in which the highest occupied and lowest unoccupied frontier orbitals are destabilized and stabilized, respectively, through the formation of frontier crystal orbitals (FCOs), as recently introduced [29]. The calculated ideal band‐like FCO contribution at 100 K is Δ*E*_FCO_ = −0.918 eV. This value reproduces the experimental shift well but, as expected, overestimates its magnitude, because the calculation assumes ideal yet physically unrealistic full coherence along the π‐stack. Treating Δ*E*_FCO_ + Δ*E*_exci_ as an upper‐limit red‐shift budget in a hypothetical fully coherent polymeric π‐stack yields an effective coherence length of *N*_coh_ ∼ 2.3 molecules at 100 K within a simple 1D tight‐binding description (Figure 3d; Supporting Information). By scaling Δ*E*_FCO_ with *N*_coh_, an effective coherence‐length‐scaled FCO contribution of $\Delta E_{\mathrm{FCO}}^{\mathrm{eff}}$ = −0.520 eV is obtained. The remaining part of the observed shift, Δ*E*_obs_ − $\Delta E_{\mathrm{FCO}}^{\mathrm{eff}}$, is then assigned to an effective excitonic contribution of $\Delta E_{\mathrm{exci}}^{\mathrm{eff}}$ = −0.241 eV (Table S15, details in Supporting Information). *N*_coh_ is largely temperature‐independent because Δ*E*_FCO_, Δ*E*_exci_, and Δ*E*_obs_ reduce in magnitude upon heating, consistent with the concurrent increase of the π–π distance (Figure 2c and 3d).

In conclusion, we have shown elusive translationally zero‐offset π‐stacks in **CP1**, a CP of an apolar purely hydrocarbon PAH enabled by adamantylethynyl‐functionalization. In **CP1**, four peripheral adamantyl substituents of **1** act as dispersion‐assisted clamps, laterally fixing the pyrene cores and yielding an exceptionally short interplanar separation of 3.297(3) Å at 100 K. The formation of aligned π‐columns of **1** gives rise to channel‐like voids sustained by solvate molecules. Energy decomposition analyses reveal that the adamantyl groups contribute a disproportionately large attractive dispersion relative to their Pauli penalty, rationalizing the stabilization of the tight packing motif. This structural motif translates directly into optoelectronic consequences. Formation of **CP1** from isolated molecules of **1** induces an exceptionally large bathochromic shift (Δ*E*_obs_ = −0.761 eV), substantially exceeding that observed for the slipped‐stacked analogue **CP2** (−0.198) [29]. The large shift of **CP1** cannot be explained by excitonic coupling alone and is instead dominated by band‐like intermolecular orbital interactions described in terms of FCOs, with an effective coherence length of *N*_coh_ ∼2.3 molecules. More broadly, these results demonstrate that dispersion—often treated as a weak and nondirectional interaction—can be deliberately engineered to control solid‐state packing and properties in CPs composed of hydrocarbon π‐systems. We are extending this dispersion‐driven approach to crystalline solid solutions [42, 43, 44], where substitutional “doping” provides an additional handle for tailoring optoelectronic properties of the crystalline solids.

## Author Contributions

**Kazutaka Shoyama**: conceptualization, data curation, formal analysis, visualization, writing – original draft, funding acquisition, writing – review and editing, and investigation.

## Conflicts of Interest

The authors declare no conflicts of interest.

## Supporting information



The authors have cited additional references within the Supporting Information [45, 46, 47, 48, 49, 50, 51, 52, 53, 54, 55, 56, 57, 58, 59, 60].
**Supporting File**: anie74326‐sup‐0001‐SuppMat.pdf.

## Data Availability

The data that support the findings of this study are available in the Supporting Information of this article.
