# Supplementary material for: Dispersion‐Clamped Crystalline Supramolecular Columnar π‐Stacks of Adamantyl‐Substituted Pyrenes
Source: Angew Chem Int Ed Engl. 2026 Aug 22;65(41):e1681939. doi: 10.1002/anie.1681939 (PMC13638111; doi:10.1002/anie.1681939)
Supplement: Supplementary file 1 [file ANIE-65-e1681939-s001.pdf]

Supporting Information

Dispersion-Clamped Crystalline Supramolecular Columnar π-Stacks of Adamantyl-Substituted Pyrenes

Kazutaka Shoyama[a,b,*]

[a] Institut für Organische Chemie, Universität Würzburg, 97074 Würzburg, Germany

[b] Center for Nanosystems Chemistry (CNC), Universität Würzburg, 97074 Würzburg, Germany

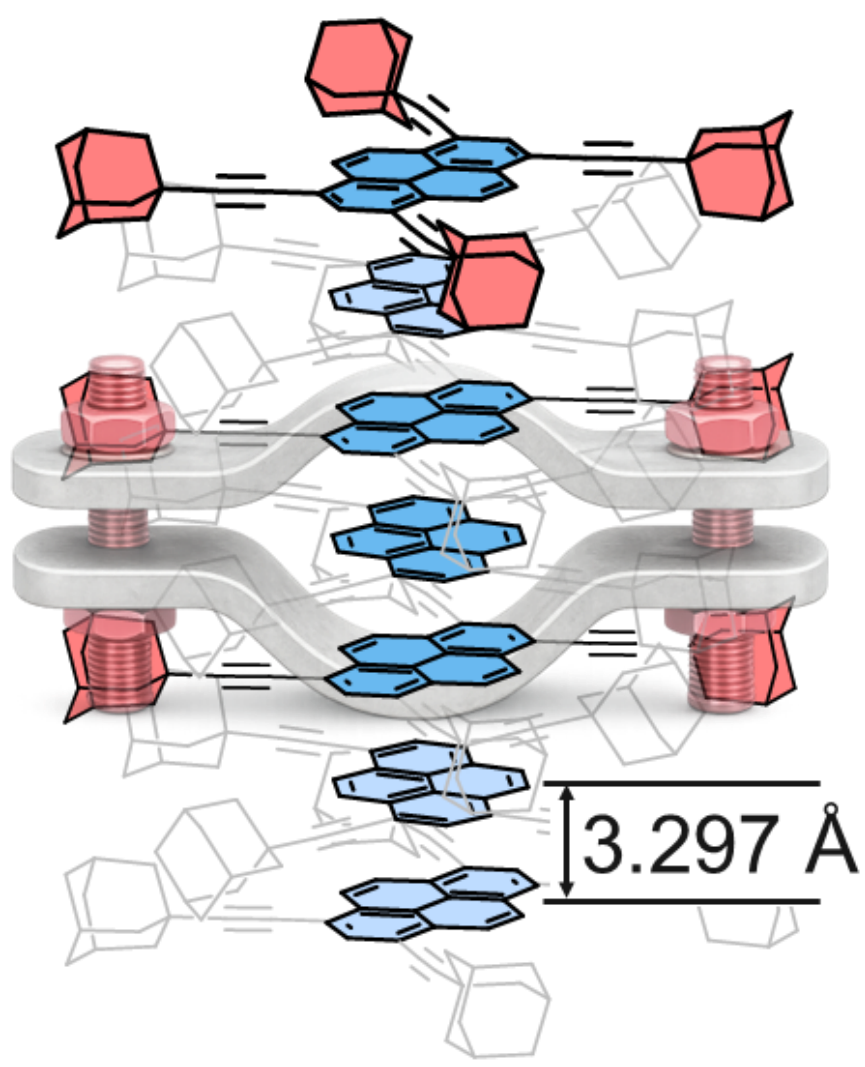

Abstract

Despite their importance in supramolecular chemistry, zero-offset columnar π-stacks of polycyclic aromatic hydrocarbons are rarely observed in crystals because close cofacial packing is penalized by Pauli-repulsion. Here we show a crystalline phase (CP) of adamantyl-substituted pyrene that exhibits one-dimensional, translationally aligned columnar π-stacks enabled by dispersion-assisted adamantyl "clamps". Single-crystal X-ray diffraction reveals an exceptionally short interplanar separation of 3.297(3) Å at 100 K. Energy-decomposition analysis shows that the adamantyl substituents provide larger dispersion stabilization relative to their Pauli penalty, rationalizing the formation of the zero-offset π-columns. The solution-to-crystal color shift is exceptionally large (ΔE_{obs} = −0.761 eV), which exceeds exciton coupling contributions and is dominated by intermolecular orbital interactions captured by a frontier crystal orbital model. To the best of our knowledge, both the translationally zero-offset π-columns and the large crystallization-induced red shift are unprecedented for ambient-pressure CPs of neutral, closed-shell hydrocarbon PAHs.

Contents

Abstract 1
1. General experimental procedures 3
Chemicals and solvents 3
Column chromatography 3
Thin-layer chromatography 3
NMR spectroscopy 3
Mass spectrometry 3
UV/Vis absorption spectroscopy 3
Fluorescence spectroscopy 3
Fluorescence microscopy 4
Quantum chemical calculations 4
2. Synthesis 5
1,3,6,8-Tetrakis[(1-adamantyl)ethynyl]pyrene (**1**) 5
3. Crystal structure analysis 7
Crystal growth 7
X-ray crystallography 9
Responses to the issues raised by the CheckCIF routine 10
Refinement results 12
Intermolecular π–π distance 13
Thermal Expansion Analysis 15
Symmetry elements 16
4. Absolutely Localized Molecular Orbital Energy Decomposition Analysis (ALMO-EDA) 17
5. Spectroscopic analysis 19
Monomer spectra in solution 19
Isolated monomer in PMMA 21
Crystalline samples on wafer 23
Fitting details 27
6. Exciton coupling 33
Coulombic exciton coupling 33
CT-mediated exciton coupling 33
Calculation of charge transfer integrals 34
Sign convention for transfer integrals 35
Combined exciton coupling 35
7. Frontier crystal orbitals (FCO) 38
Overview of frontier molecular orbital interactions in an organic crystal 38
Estimation of the FCO contribution to the solution-to-crystal shift 40
Coherence lengths 44
8. Polarization optical microscopy (POM) 49

1. General experimental procedures

Chemicals and solvents

Chemicals and solvents were purchased from commercial suppliers and used without further purification unless otherwise noted.

Column chromatography

Column chromatography was performed using standard glass columns of different sizes, packed with silica-gel (particle size: 40–63 µm) purchased from Macherey-Nagel as stationary phase.

Thin-layer chromatography

For monitoring of the reaction progress, thin layer chromatography (TLC) was carried out, using TLC plates ALUGRAM®Xtra SIL G/UV254 (layer thickness: 0.2 mm) purchased from Marcherey-Nagel.

NMR spectroscopy

^{1}H and ^{13}C NMR spectra were recorded on Avance III HD 400 or Bruker Avance III HD 600 spectrometers. Chemical shifts *δ* are given in ppm and *J* (coupling constants) in Hz. For all multiplicities, the following abbreviations were used: s = singlet, d = doublet, dd = doublet of doublet, m = multiplet. Residual solvent signals were used for calibration: ^{1}H NMR in CD_2Cl_2 δ = 5.30 ppm.

Mass spectrometry

MALDI-TOF mass spectra were recorded on a MALDI-TOF MS ultrafleXtreme from Bruker Daltonics GmbH in positive-ion mode using *trans*-2-[3-(*tert*-butylphenyl)-2-methyl-2-propenylidene]malo-nonitrile (DCTB) as the matrix.

UV/Vis absorption spectroscopy

UV/Vis absorption spectra were measured with a V-770 spectrophotometer (Jasco) equipped with a JASCO PAC-743R Auto Peltier 6/8-cell changer system for temperature control. Standard quartz glass cuvettes of different path lengths (Hellma) and spectroscopy grade solvents were used.

Fluorescence spectroscopy

Photoluminescence measurements were performed using an Edinburgh Instruments FLS980 spectrometer. NIR emission was detected using a liquid-nitrogen-cooled Hamamatsu R5509-42 NIR photomultiplier tube. Time-resolved measurements employed either an AGILE picosecond pulsed laser source coupled to the excitation monochromator of the spectrometer or pulsed EPL diode lasers for excitation, with fluorescence decays recorded by time-correlated single-photon counting (TCSPC). All spectra were corrected for the wavelength-dependent photomultiplier tube sensitivity and the spectral intensity profile of the excitation source.

Solution measurements were carried out in quartz cuvettes. Solid-state measurements were performed in front-face geometry using ensembles of single crystals immersed in a minimal amount of mineral oil and mounted on quartz substrates.

Variable-temperature photoluminescence measurements of crystal ensembles on Si/SiO_2 wafer were carried out using an Oxford Instruments OptistatDN cryogenic sample holder combined with an Oxford Instruments MercuryiTC temperature controller.

Fluorescence microscopy

Fluorescence microscopy of micrometer-sized single crystals in mineral oil on quartz substrates was recorded with a Zeiss Axio Imager optical polarization microscope equipped with a Hg arc lamp for excitation with UV light.

Quantum chemical calculations

Unless otherwise noted, quantum chemical calculations based on density functional theory (DFT) and time-dependent density functional theory (TD-DFT) were carried out using the Gaussian 16 program package,[S1] and molecular structures and orbitals were visualized with GaussView 6.[S2]

2. Synthesis

1,3,6,8-Tetrakis[(1-adamantyl)ethynyl]pyrene (1)[S3]

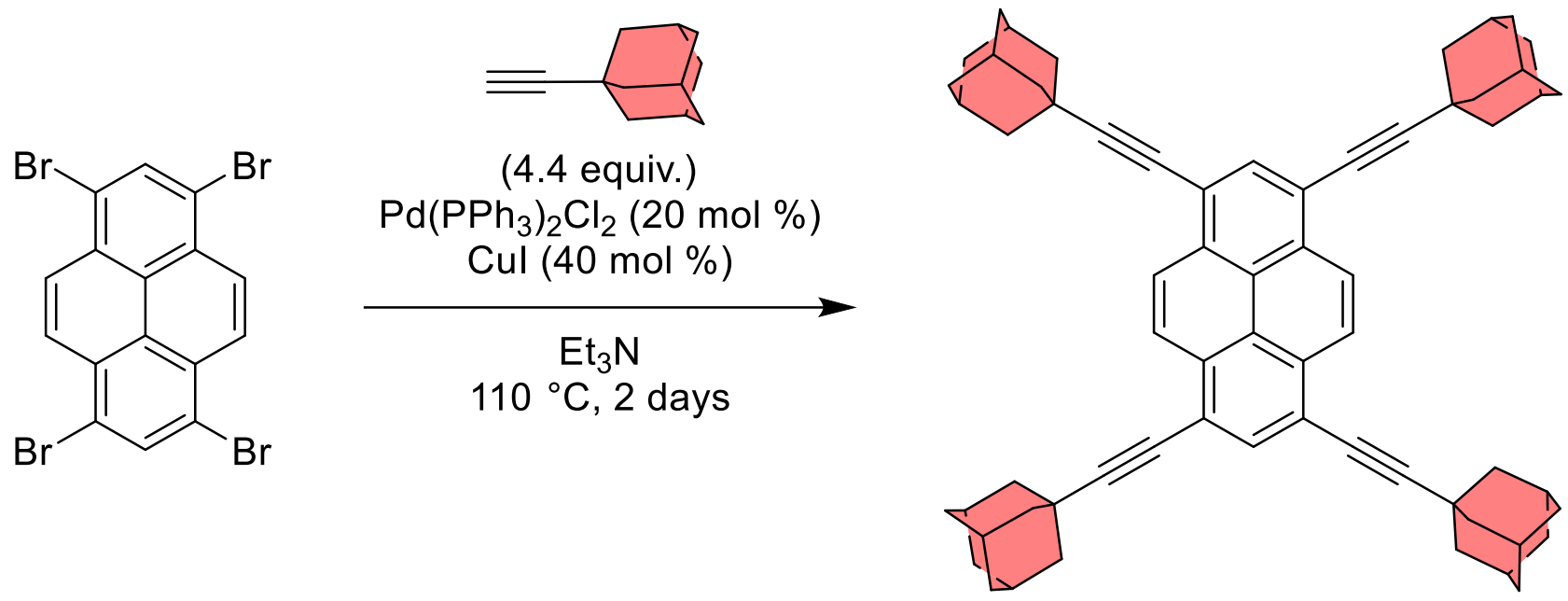

1,3,6,8-Tetrabromopyrene (311 mg, 0.600 mmol), 1-ethynyladamantane (423 mg, 4.40 equiv.,2.64 mmol), CuI (45.7 mg, 40.0 mol %, 0.240 mmol) and $Pd(PPh_3)_2Cl_2$ (84.2 mg, 20.0 mol %, 0.120 mmol) were charged in a Young Schlenk tube under nitrogen atmosphere. Then degassed triethylamine (6 mL) was added and the mixture was stirred for 2 days at 110 °C. After cooling to room temperature, the mixture was diluted with dichloromethane and remaining solids were filtered off via a short pad of silica-gel (eluent: dichloromethane). After removal of solvent the crude product was purified by column chromatography (silica-gel, hexane:dichloromethane = 10:0 to 9:1, only very middle fractions were collected to ensure high purity for spectroscopic measurements) to give **1** as a yellow solid (198 mg, 40%). Note: this compound is not entirely stable in stabilizer-free chloroform, therefore NMR experiments were conducted in dichloromethane. Mp: >300 °C, ^{1}H NMR (400 MHz, CD_2Cl_2, 295 K): δ = 8.51 (s, 4H), 8.09 (s, 2H), 2.14–2.10 (m, 24H), 2.08–2.03 (m, 12H), 1.84–1.74 (m, 24H). HRMS (MALDI-TOF, pos. mode, chloroform/DCTB, 1:3): (m/z) [M]$^+$, calculated for $C_{64}H_{66}$: 834.5165, found: 834.51389.

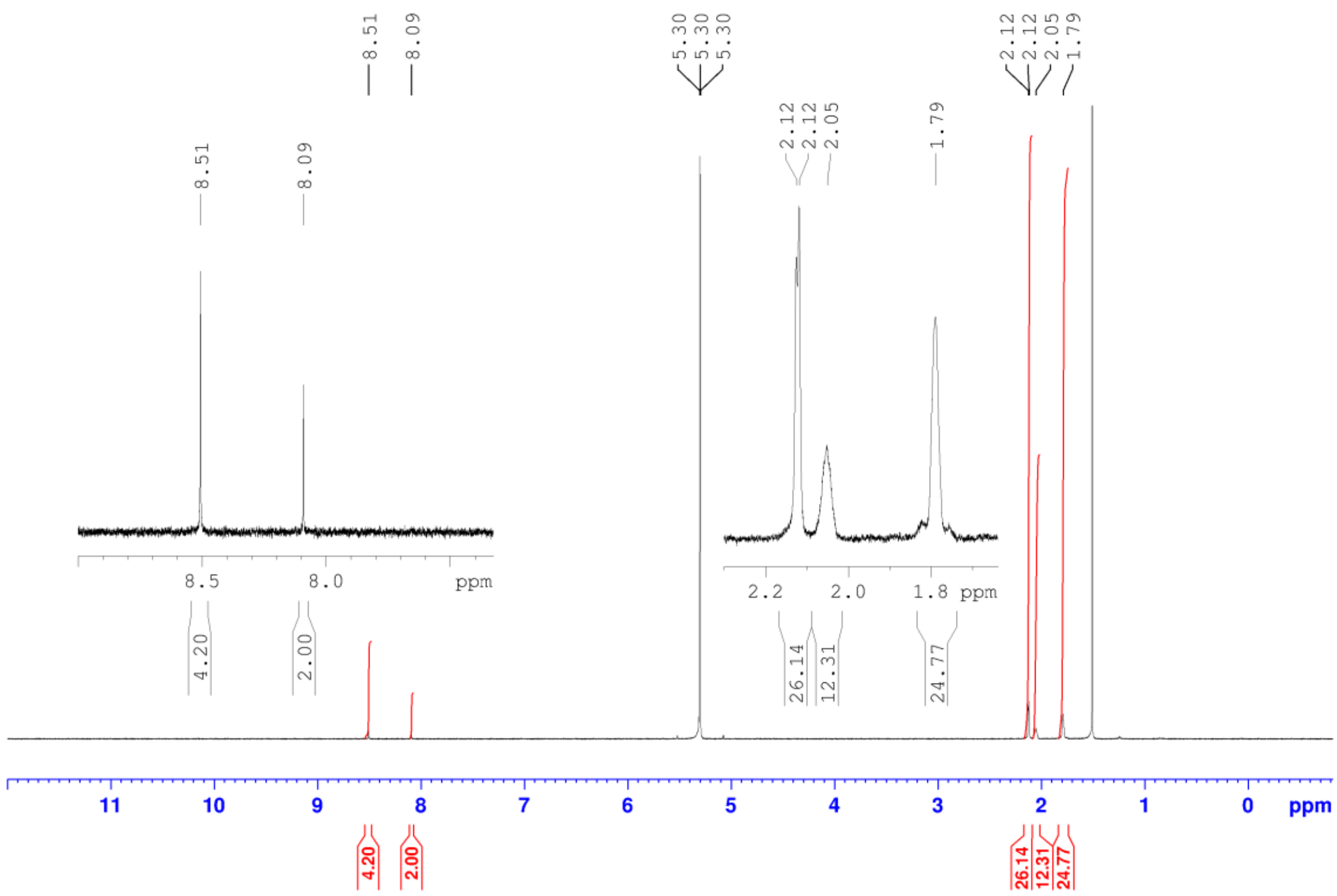

Figure S1. ^{1}H NMR spectrum (400 MHz, 295 K, CD_2Cl_2) of **1**.

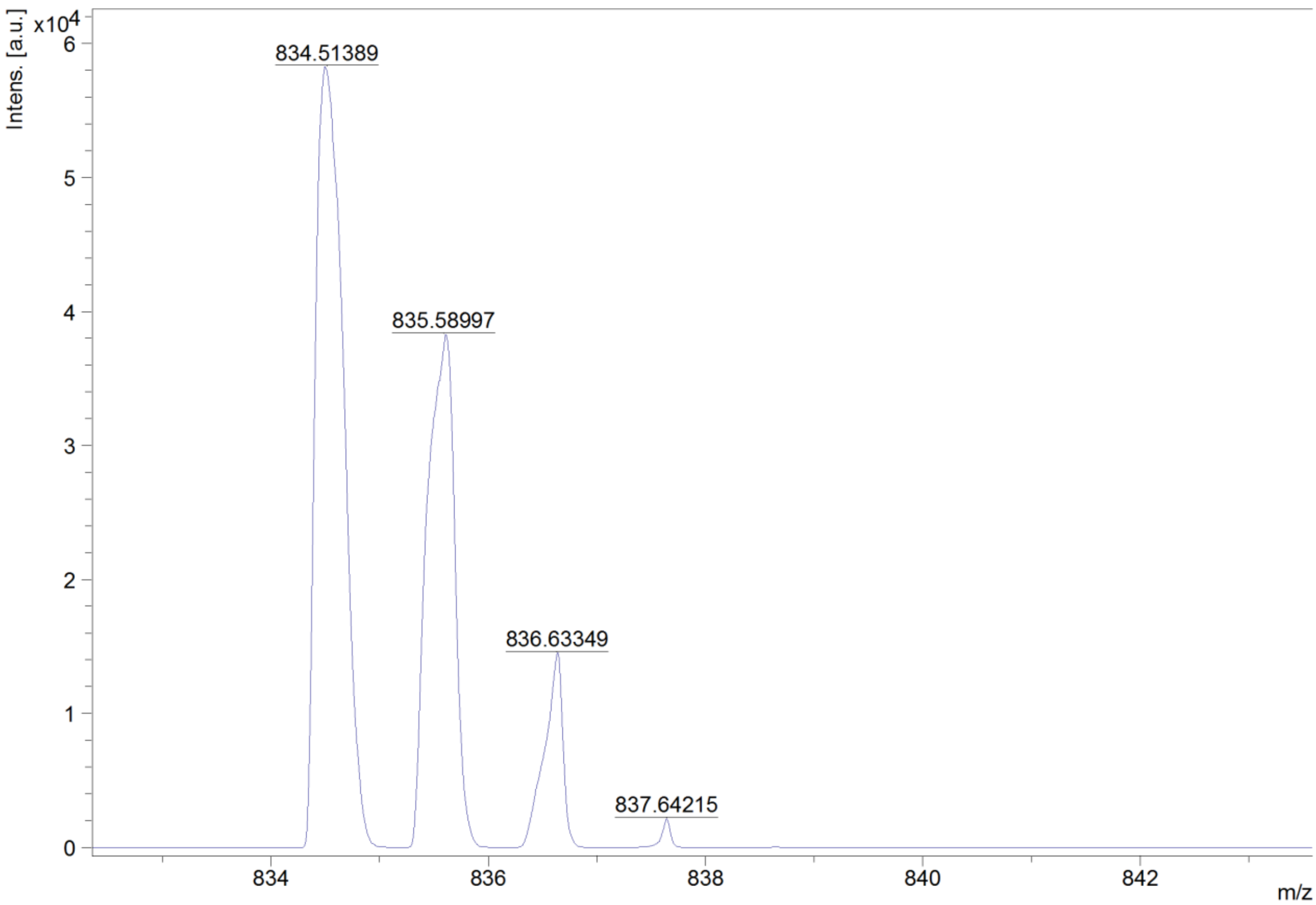

Figure S2. MALDI-TOF mass spectrum of compound **1** in positive-ion mode.

3. Crystal structure analysis

Crystal growth

Crystallization of **1** by slow diffusion of *n*-hexane into a dichloromethane solution of **1** (c(**1**) < 1×10^{-4} M, 293 K) over one week in a vibration-suppressed, temperature-controlled incubator yielded orange, needle-shaped crystals of **CP1** = [**1**·(*n*-hexane)]. At higher concentrations (c(**1**) ≥ 1×10^{-4} M) , the non-solvated crystalline phase **CP2** = [**1**] crystallized as yellow plate-like crystals and was not investigated further in this study.[S3] We further note that the purity of the substrate strongly affects the crystallization behavior of this compound: lower purity leads to substantially higher solubility than that of the pure material, as is commonly expected for impure substances. This increased solubility is assumed to favor formation of the yellow nonsolvated crystalline phase **CP2**, presumably because it results in higher concentrations at the onset of nucleation.

Typical crystallization procedure: A stock solution of **1** in dichloromethane was prepared by dissolving the solid sample in spectroscopic-grade dichloromethane. Complete dissolution was ensured by brief ultrasonication or agitation using a vortex mixer. The resulting solution was passed through a PVDF membrane filter with a 0.45 μm pore size and transferred into a small inner vial. The inner vial was placed inside a larger outer vial containing spectroscopic-grade hexane as the poor solvent. The outer vial was closed with a lid and kept in an incubator at 293 K to allow slow vapor diffusion of hexane into the dichloromethane solution over ca. one week.

Table S1. Relationship between crystallization conditions and the resulting crystalline phase.[a]

Entry	Concentration (M)	Crystal formation	Color	Shape	Crystalline phase
**1**[b]	2.0×10^{-4}	Yes	Yellow	Plate	**CP2**
**2**	1.0×10^{-4}	Yes	Yellow	Plate	**CP2**
**3**	5.0×10^{-5}	Yes	Orange	Needle	**CP1**
**4**	2.5×10^{-5}	Yes	Orange	Needle	**CP1**

[a] Conditions: vapor diffusion using dichloromethane as the good solvent and hexane as the poor solvent, with hexane diffusing into a dichloromethane solution of **1** at 293 K. [b] Compound **1** was not completely dissolved; the concentration exceeded the solubility limit.

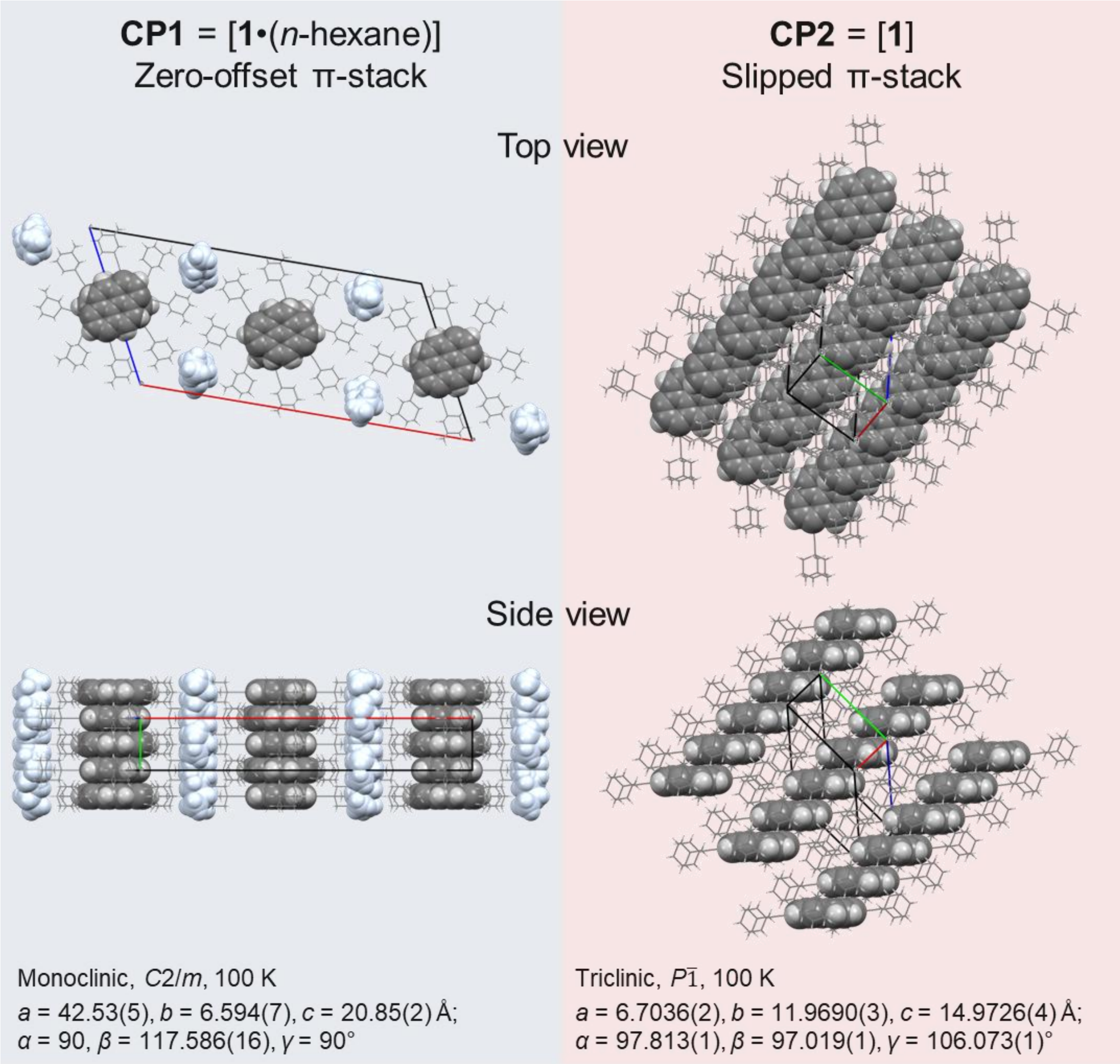

Figure S3. Packing structures of the two crystalline phases of 1. Top and side views of **CP1** = [**1**·(*n*-hexane)] and **CP2** = [**1**][S3] as well as unit cell information.

X-ray crystallography

Single-crystal X-ray diffraction data were collected at the P11 beamline at DESY using a single 360° φ scan. The diffraction data were indexed, integrated, and scaled using the XDS program package.[S4] The structures were solved using SHELXT,[S5] expanded by Fourier techniques, and refined using the SHELXL[S6] software package. Electron-density maps were generated with ShelXle[S7] and inspected throughout the analysis. Hydrogen atoms were placed in idealized positions and included in the calculation of structure factors. All non-hydrogen atoms in the main residue were refined anisotropically. Disordered side chains were modelled with restraints using standard SHELXL commands.

Variable-temperature single-crystal X-ray diffraction measurements were performed at the P11 beamline on the same crystal. Temperature control was achieved using a custom-built interface installed at P11. Diffraction data were collected first at 90 K and then at 100 K, followed by measurements in 20 K increments up to 280 K, and finally at 298 K.

The structure exhibits whole-molecule disorder (Figure S4). For each molecular unit in the asymmetric unit containing two crystallographically independent molecules, two molecular orientations occupying the same crystallographic site were modelled, differing by an approximate rotation of 45° along the principal molecular axis (molecular axis perpendicular to the π-surface). The disorder was treated using standard restraint commands implemented in SHELXL. Owing to the whole-molecule disorder, the entire pyrene core was modelled using DFIX restraints, with distance parameters taken from the crystal structure of **CP2** (ref. S3). The adamantyl side chains were additionally modelled using DFIX and DANG restraints, with restraint parameters taken from ref. S8. Owing to this whole-molecule disorder, the positional parameters of individual atoms are associated with relatively large standard uncertainties, which also led to comparatively large deviations in the DFT-calculated dimer interaction energies (*vide infra*). Nevertheless, the π–π distance could be estimated with good reliability, because each disordered molecule is located on the symmetry plane (Figure S5), such that the definition of the molecular plane is much less affected by the disorder than the individual atomic positions.

Verification of crystallographic data of **CP1** = [**1**·(*n*-hexane)] using the CheckCIF[S9] routine implemented in the PLATON program package[S10] showed several level A and B Alerts over the entire temperature range between 90–298 K. These alerts primarily arise from weak diffraction intensities associated with the large unit cell and whole-molecule disorder, which also lead to elevated *R* values. These Alerts are rationalized below and in the corresponding CIFs.

Responses to the issues raised by the CheckCIF routine

PLAT026

PROBLEM: Ratio Observed / Unique Reflections (too) Low

RESPONSE: The low observed/unique reflection ratio is due to the weak diffraction intensity of the crystal, in particular at higher diffraction angles, combined with severe disorder in the structure. As a result, many unique reflections did not meet the I > 2sigma(I) criterion.

PLAT084

PROBLEM: High wR2 Value (i.e. > 0.25)

RESPONSE: The high wR2 value is a consequence of the pronounced whole-molecule/side-chain disorder, large atomic displacement parameters in parts of the structure, and limited data quality caused by weak high-angle diffraction. Despite this, the connectivity and overall molecular arrangement are unambiguous, and the refinement is considered the best achievable model for the available data

PLAT260

PROBLEM: Large Average Ueq of Residue Including C1_101

RESPONSE: Residue 101 is a severely disordered hexane solvate molecule with partial occupancy. The large average Ueq reflects substantial disorder-related displacement in this part of the structure rather than an assignment error. Appropriate restraints were applied during refinement to maintain a chemically reasonable model.

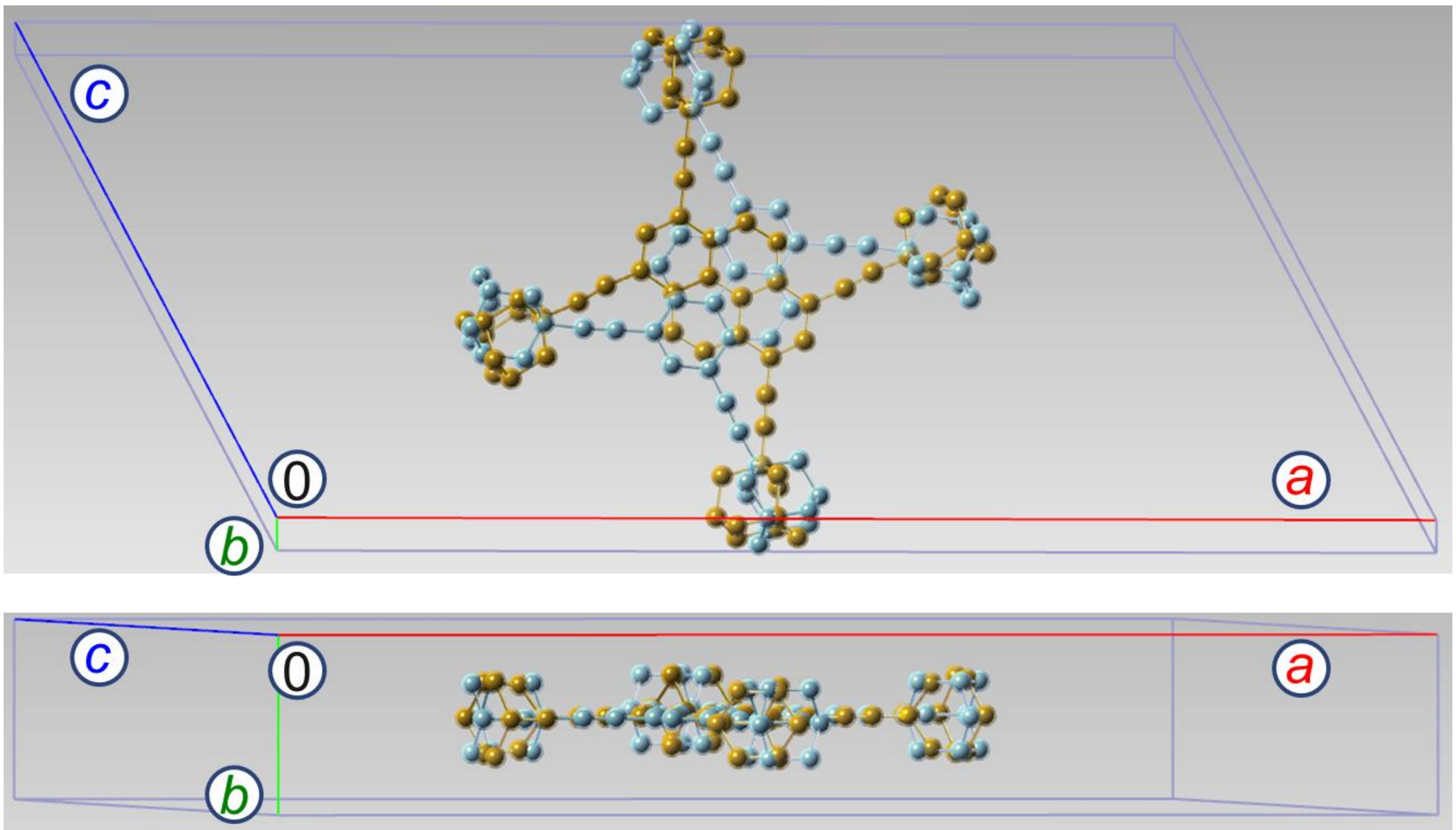

Figure S4. Whole-molecule disorder of **CP1** in the crystal structure, shown as the two alternative molecular orientations within the same crystallographic site. Top: view onto the *a*–*c* plane. Bottom: side view approximately along the π-stacking direction.

Refinement results

Table S2. Refinement parameters for the crystals structure of CP1 at 90–298 K.

Parameter	90 K	100 K	120 K	140 K	160 K	180 K	200 K	220 K	240 K	260 K	280 K	298 K
**CCDC number**	2544930	2544931	2544932	2544933	2544934	2544935	2544936	2544937	2544938	2544939	2544940	2544941
**Empirical formula**	$C_{140}H_{160}$	$C_{140}H_{160}$	$C_{140}H_{160}$	$C_{140}H_{160}$	$C_{140}H_{160}$	$C_{140}H_{160}$	$C_{140}H_{160}$	$C_{140}H_{160}$	$C_{140}H_{160}$	$C_{140}H_{160}$	$C_{140}H_{160}$	$C_{140}H_{160}$
**Formula wt (g/mol^{-1})**	1842.67	1842.67	1842.67	1842.67	1842.67	1842.67	1842.67	1842.67	1842.67	1842.67	1842.67	1842.67
**Temperature (K)**	90(2)	100(2)	120(2)	140(2)	160(2)	180(2)	200(2)	220(2)	240(2)	260(2)	280(2)	298(2)
**Wavelength (Å)**	0.61992	0.61992	0.61992	0.61992	0.61992	0.61992	0.61992	0.61992	0.61992	0.61992	0.61992	0.61992
**Crystal system**	Monoclinic	Monoclinic	Monoclinic	Monoclinic	Monoclinic	Monoclinic	Monoclinic	Monoclinic	Monoclinic	Monoclinic	Monoclinic	Monoclinic
**Space group**	*C*2/*m*	*C*2/*m*	*C*2/*m*	*C*2/*m*	*C*2/*m*	*C*2/*m*	*C*2/*m*	*C*2/*m*	*C*2/*m*	*C*2/*m*	*C*2/*m*	*C*2/*m*
***a* (Å)**	42.52(3)	42.53(5)	42.58(2)	42.659(15)	42.729(18)	42.80(2)	42.849(19)	42.917(13)	42.974(9)	43.040(11)	43.084(10)	43.130(14)
***b* (Å)**	6.591(5)	6.594(7)	6.608(4)	6.624(4)	6.638(4)	6.653(5)	6.665(4)	6.681(4)	6.6960(18)	6.714(2)	6.734(2)	6.751(2)
***c* (Å)**	20.849(18)	20.85(2)	20.858(13)	20.881(7)	20.902(9)	20.922(9)	20.937(9)	20.962(7)	20.976(5)	21.000(5)	21.016(5)	21.032(7)
**α (°)**	90	90	90	90	90	90	90	90	90	90	90	90
**β (°)**	117.575(12)	117.586(16)	117.571(15)	117.556(18)	117.557(10)	117.548(12)	117.549(6)	117.564(6)	117.552(4)	117.537(5)	117.510(7)	117.491(6)
**γ (°)**	90	90	90	90	90	90	90	90	90	90	90	90
**Volume (Å^3)**	5180(7)	5183(10)	5202(5)	5231(4)	5256(4)	5282(5)	5301(5)	5328(4)	5351(2)	5381(3)	5408(2)	5432(3)
Z	2	2	2	2	2	2	2	2	2	2	2	2
**Density (calc) (g/cm^{-3})**	1.182	1.181	1.176	1.170	1.164	1.159	1.154	1.149	1.144	1.137	1.132	1.127
**μ (mm^{-1})**	0.051	0.051	0.051	0.051	0.051	0.050	0.050	0.050	0.050	0.049	0.049	0.049
***F*(000)**	2000	2000	2000	2000	2000	2000	2000	2000	2000	2000	2000	2000
**Crystal size (mm)**	0.02 × 0.003 × 0.003	0.02 × 0.003 × 0.003	0.02 × 0.003 × 0.003	0.02 × 0.003 × 0.003	0.02 × 0.003 × 0.003	0.02 × 0.003 × 0.003	0.02 × 0.003 × 0.003	0.02 × 0.003 × 0.003	0.02 × 0.003 × 0.003	0.02 × 0.003 × 0.003	0.02 × 0.003 × 0.003	0.02 × 0.003 × 0.003
**θ range for data collection (°)**	0.942 to 28.103	0.942 to 28.098	0.941 to 28.067	0.939 to 28.048	0.938 to 28.000	0.936 to 28.103	0.935 to 28.066	0.934 to 28.064	0.932 to 28.022	0.931 to 28.046	0.930 to 28.053	0.928 to 28.019
**Index ranges**	$-62 \le h \le 61$, $-9 \le k \le 9$, $-29 \le l \le 29$	$-62 \le h \le 61$, $-9 \le k \le 9$, $-29 \le l \le 29$	$-62 \le h \le 61$, $-9 \le k \le 9$, $-29 \le l \le 29$	$-62 \le h \le 61$, $-9 \le k \le 9$, $-29 \le l \le 29$	$-62 \le h \le 61$, $-9 \le k \le 9$, $-29 \le l \le 29$	$-62 \le h \le 61$, $-9 \le k \le 9$, $-29 \le l \le 29$	$-62 \le h \le 63$, $-9 \le k \le 9$, $-29 \le l \le 29$	$-63 \le h \le 63$, $-9 \le k \le 9$, $-29 \le l \le 29$	$-64 \le h \le 63$, $-9 \le k \le 9$, $-29 \le l \le 29$	$-64 \le h \le 63$, $-9 \le k \le 10$, $-29 \le l \le 29$	$-64 \le h \le 63$, $-9 \le k \le 10$, $-29 \le l \le 29$	$-64 \le h \le 63$, $-9 \le k \le 10$, $-29 \le l \le 30$
**Reflections collected**	50052	50153	50297	50540	50655	50887	51172	51456	51615	51624	52030	52311
**Unique reflections**	7862	7897	7916	7917	7971	7989	8030	8084	8112	8101	8182	8233
**R_{int}**	0.0823	0.0808	0.0716	0.0617	0.0606	0.0687	0.0703	0.0717	0.0659	0.0644	0.0647	0.0703
**Completeness to θ**	98.6%, 21.8°	98.7%, 21.8°	98.7%, 21.8°	98.7%, 21.8°	98.6%, 21.8°	98.7%, 21.8°	98.5%, 21.8°	98.7%, 21.8°	98.5%, 21.8°	98.5%, 21.8°	98.4%, 21.8°	98.5%, 21.8°
**Absorption correction**	None	None	None	None	None	None	None	None	None	None	None	None
**Refinement method**	Full-matrix LS on F^2	Full-matrix LS on F^2	Full-matrix LS on F^2	Full-matrix LS on F^2	Full-matrix LS on F^2	Full-matrix LS on F^2	Full-matrix LS on F^2	Full-matrix LS on F^2	Full-matrix LS on F^2	Full-matrix LS on F^2	Full-matrix LS on F^2	Full-matrix LS on F^2
**Data / restraints / parameters**	7862 / 2935 / 859	7897 / 2935 / 859	7916 / 2935 / 859	7917 / 2935 / 859	7971 / 2932 / 859	7989 / 2935 / 859	8030 / 2935 / 859	8084 / 2935 / 859	8112 / 2935 / 859	8101 / 2935 / 859	8182 / 2935 / 859	8233 / 2935 / 859
**Goodness-of-fit on F^2**	0.956	0.957	0.950	0.938	0.931	0.908	0.915	0.920	0.944	0.920	0.921	0.931
**R_1 [$I > 2\sigma(I)$]**	0.1019	0.1028	0.0943	0.0861	0.0807	0.0813	0.0852	0.0858	0.0904	0.0896	0.0932	0.0975
**wR_2 [$I > 2\sigma(I)$]**	0.2823	0.2845	0.2657	0.2468	0.2412	0.2367	0.2434	0.2467	0.2558	0.2485	0.2550	0.2698
**R_1 (all data)**	0.2507	0.2459	0.2143	0.1875	0.1740	0.1719	0.1866	0.2020	0.1895	0.1999	0.2190	0.2343
**wR_2 (all data)**	0.4133	0.4076	0.3813	0.3546	0.3425	0.3351	0.3512	0.3668	0.3600	0.3671	0.3845	0.4032
**Extinction coefficient**	n/a	n/a	n/a	n/a	n/a	n/a	n/a	n/a	n/a	n/a	n/a	n/a
**Largest diff. peak and hole (e Å^{-3})**	0.140 and −0.211	0.135 and −0.218	0.130 and −0.195	0.150 and −0.193	0.199 and −0.194	0.165 and −0.188	0.186 and −0.189	0.191 and −0.189	0.220 and −0.208	0.191 and −0.186	0.154 and −0.234	0.167 and −0.198

Intermolecular π–π distance

The intermolecular π–π distance of **CP1** ($d_{\pi-\pi}$) was derived from the lattice parameters obtained from single-crystal X-ray diffraction analysis. Because the π-surface of the pyrene core in **CP1** lies in the *a*–*c* plane of the monoclinic lattice, and two molecules of **1** stack along the *b* axis within one unit cell, half of the unit-cell parameter *b* corresponds exactly to the π–π distance of the directly π-stacked dimer ($d_{\pi-\pi} = b/2$). Weighted linear regression using the experimental standard uncertainties of *b* yielded

$$d_{\pi-\pi}(\mathrm{T}) = 3.25618 + 3.91674 \times 10^{-4}\ T$$

, where $d_{\pi-\pi}$ is expressed in Å and *T* in K.

Table S3. Temperature-dependent crystallographic *b* parameter, derived $d_{\pi-\pi}$, and comparison with the interlayer spacing of graphite.[a]

**Temperature**	***b***	***σ*(*b*)**	**$d_{\pi-\pi}$**	***σ*(π–π)**	**Graphite**	**$d_{\pi-\pi}$ (fit)**
K	Å	Å	Å	Å	Å	Å
**90**	6.591	0.0047	3.296	0.0024	3.341	3.291
**100**	6.594	0.0067	3.297	0.0034	3.342	3.295
**120**	6.608	0.0036	3.304	0.0018	3.343	3.303
**140**	6.624	0.0037	3.312	0.0019	3.345	3.311
**160**	6.638	0.0039	3.319	0.0020	3.347	3.319
**180**	6.653	0.0047	3.327	0.0024	3.349	3.327
**200**	6.665	0.0039	3.333	0.0020	3.351	3.335
**220**	6.681	0.0035	3.341	0.0018	3.353	3.342
**240**	6.696	0.0018	3.348	0.0009	3.355	3.350
**260**	6.714	0.0020	3.357	0.0010	3.357	3.358
**280**	6.734	0.0021	3.367	0.0011	3.359	3.366
**298**	6.751	0.0021	3.376	0.0011	3.360	3.373

[a] *b*: crystallographic *b* axis parameter obtained from variable-temperature single-crystal X-ray diffraction; *σ*(*b*): estimated standard uncertainty of *b*; $d_{\pi-\pi}$: intermolecular π–π distance as *b*/2; *σ*(π–π): corresponding propagated uncertainty, obtained as *σ*(*b*)/2. Graphite: temperature-dependent interlayer distance of graphite calculated from the literature expression $3.332 + 9.52 \times 10^{-5}\ T$ Å, where *T* is in K.[S11] $d_{\pi-\pi}$ (fit): π–π distance calculated from the linear fit to the experimental data, $3.25618 + 3.91674 \times 10^{-4} T$ Å. All distances are given in Å.

As a benchmark for comparison with the thermal expansion of the zero-offset π-stacks in this study, γ-coronene was used as a reference crystalline hydrocarbon PAH. An ideal reference would be an unsubstituted PAH crystal with a perfectly cofacial, zero-offset π–π stack. However, such a structurally comparable reference is not available in the literature. Coronene was therefore selected as a practical benchmark because its crystal structure contains comparatively shallow slipped π–π stacking between disk-like PAH molecules, while retaining a hydrocarbon-only composition. In contrast, many smaller acenes, such as naphthalene, anthracene, tetracene, and pentacene, adopt pronounced herringbone-type packing motifs with more strongly slipped or edge-to-face intermolecular arrangements. These structures were therefore not used as primary references for comparison with the present translationally zero-offset π-stack.

The temperature-dependent unit-cell parameters of γ-coronene reported by Litasov et al.[S12] were used to estimate the thermal expansion along the crystallographic *b*-axis. The *b*-axis parameter is relevant because it corresponds to the short crystallographic direction associated with the π-stacking separation in the γ-coronene crystal. For comparison with the present variable-temperature single-crystal X-ray diffraction data, the analysis was restricted to the temperature range below room temperature. A linear fit of the reported *b*-axis lengths between 185 and 298 K gave

$$\frac{db}{dT} = 1.24 \times 10^{-4}\ \text{Å}\ \text{K}^{-1}$$

. Using b = 4.695 Å at 298 K, this corresponds to a linear thermal expansion coefficient of

$$\alpha_b = \frac{1}{b}\frac{db}{dT} = \frac{1.24 \times 10^{-4}}{4.695} = 2.64 \times 10^{-5}\ \text{K}^{-1} = 26\ \text{ppm}\ \text{K}^{-1}$$

. This value provides a useful reference for the thermal expansion of a pristine hydrocarbon PAH crystal with appreciable π–π stacking. The *b*-axis expansion of γ-coronene below 298 K is modest in absolute terms, corresponding to only ca. 0.012 Å per 100 K. Comparison with this value therefore provides a conservative benchmark for evaluating whether the π-stacking direction in the present zero-offset π-stacking crystal exhibits unusually large thermal expansion.

Thermal Expansion Analysis

The temperature dependence of the unit-cell parameters was evaluated from variable-temperature single-crystal X-ray diffraction data collected between 90 and 298 K. Average linear thermal expansion coefficients along the crystallographic axes were obtained from linear fits of the lattice parameters *a*, *b*, and *c* versus temperature (*T*) according to

$$\alpha_i = \frac{1}{i}\left(\frac{di}{dT}\right)$$

Where *i* = *a*, *b*, or *c*. For the monoclinic unit cell, the volumetric thermal expansion coefficient was determined from

$$\alpha_V = \frac{1}{V}\left(\frac{dV}{dT}\right)$$

with $V = a*b*c*\sin(\beta)$.

To account for the experimental precision of the variable-temperature diffraction data, the fits were performed as weighted linear regressions using the standard uncertainties of the lattice parameters listed in Table S4. The uncertainties of the thermal expansion coefficients were propagated from the corresponding fit uncertainties.

This analysis gave average linear thermal expansion coefficients of 70.7 ± 1.8, 117.6 ± 2.1, and 45.4 ± 1.8 ppm K^{-1} for the *a*, *b*, and *c* axes, respectively, over the temperature range 90–298 K. The corresponding volumetric thermal expansion coefficient was 237.3 ± 3.3 ppm K^{-1}.

Table S4. Parameters used for deriving thermal expansion parameters.[a]

T	*a*	*b*	*c*	*α*	*β*	*γ*	*σ*(*a*)	*σ*(*b*)	*σ*(*c*)	*σ*(*α*)	*σ*(*β*)	*σ*(*γ*)
K	Å	Å	Å	°	°	°	Å	Å	Å	°	°	°
**90**	42.523	6.591	20.849	90	117.575	90	0.032	0.0047	0.018	0	0.012	0
**100**	42.533	6.594	20.851	90	117.586	90	0.046	0.0067	0.023	0	0.016	0
**120**	42.580	6.608	20.858	90	117.571	90	0.024	0.0036	0.013	0	0.015	0
**140**	42.659	6.624	20.881	90	117.556	90	0.015	0.0037	0.0072	0	0.018	0
**160**	42.729	6.638	20.902	90	117.557	90	0.018	0.0039	0.0091	0	0.010	0
**180**	42.797	6.653	20.922	90	117.548	90	0.020	0.0047	0.0092	0	0.012	0
**200**	42.849	6.665	20.937	90	117.549	90	0.019	0.0039	0.0090	0	0.0061	0
**220**	42.917	6.681	20.962	90	117.564	90	0.013	0.0035	0.0065	0	0.0063	0
**240**	42.974	6.696	20.976	90	117.552	90	0.0092	0.0018	0.0045	0	0.0043	0
**260**	43.04	6.714	21.000	90	117.537	90	0.011	0.0020	0.0052	0	0.0055	0
**280**	43.084	6.734	21.016	90	117.510	90	0.010	0.0021	0.0045	0	0.0072	0
**298**	43.13	6.751	21.032	90	117.491	90	0.014	0.0021	0.0066	0	0.0056	0

[a] *T*: temperature (K). *a*, *b*, *c*, *α*, β, γ: unit-cell parameters; *σ*(*a*), *σ*(*b*), *σ*(*c*), *σ*(*α*), *σ*(*β*), *σ*(*γ*): experimental standard uncertainties of the corresponding unit-cell parameters in parentheses. *α* and *γ* are fixed at 90° for the monoclinic crystal system, and their standard uncertainties are therefore given as 0.

Symmetry elements

The crystallographic symmetry elements of the monoclinic unit cell further illustrate the perfectly zero-offset π-stacks of **1** in **CP1** (Figure S5). In this lattice, the *b* axis is exactly orthogonal to the *a*–*c* plane. The pyrene π-surface lies exactly on a crystallographic mirror plane, while the center of each molecule is located on an inversion center. At the same time, the zero-offset π-stack is oriented exactly along the crystallographic two-fold axis, so that the stacking direction is strictly parallel to the normal of the π-surface. As a result, the molecules form an exactly aligned columnar stack with perfect translational registry and no lateral slip between neighboring π-cores.

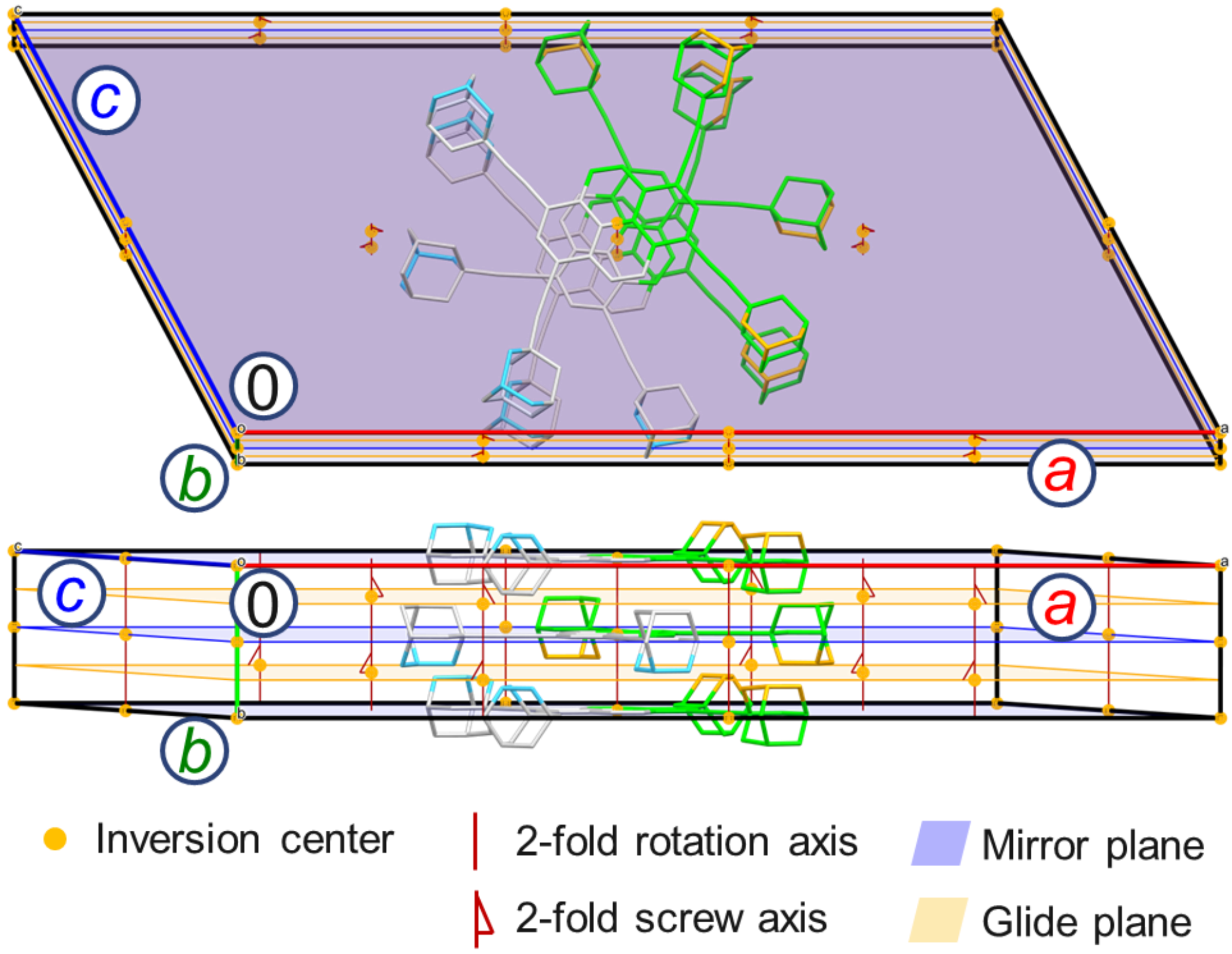

Figure S5. Crystallographic symmetry elements in the unit cell of **CP1** and their relation to the zero-offset π-stacking motif. Top: view onto the *a*–*c* plane showing the unit-cell symmetry elements and the molecular arrangement. Bottom: side view highlighting the perfectly columnar π-stack. Yellow dots: inversion centers; red lines: two-fold rotation axes; red arrows: two-fold screw axes; blue planes: mirror planes; orange planes: glide planes.

4. Absolutely Localized Molecular Orbital Energy Decomposition Analysis (ALMO-EDA).

Energy decomposition analyses were carried out using the ALMO-EDA scheme[S13] as implemented in Q-Chem 5.1.[S14] Calculations were performed on molecular dimers extracted from the crystal structures without further geometry optimization at the B3LYP/6-311G(d) level of theory. In this approach, the total interaction energy (E_{total}) between two fragments is partitioned into physically meaningful contributions according to

$$E_{\mathrm{total}} = E_{\mathrm{elec}} + E_{\mathrm{Pauli}} + E_{\mathrm{pol}} + E_{\mathrm{CT}} + E_{\mathrm{disp}}$$

where E_{elec}, E_{Pauli}, E_{pol}, E_{CT}, and E_{disp} denote the electrostatic, Pauli repulsion, polarization, charge-transfer, and dispersion contributions, respectively. The electrostatic term describes the classical Coulombic interaction between the unrelaxed fragment charge distributions, whereas the Pauli term accounts for the destabilizing interaction arising from antisymmetrization of the fragment wave functions. The polarization term describes stabilization arising from intrafragment orbital relaxation in the field of the partner fragment under the constraint of zero interfragment charge transfer, whereas the charge-transfer term reflects stabilization associated with interfragment electron delocalization.

For the analysis of intermolecular interactions in the crystal, each molecule was treated as an individual fragment. The resulting energy components were used to assess the relative importance of electrostatic, Pauli repulsion, dispersion, polarization, and charge-transfer contributions to the intermolecular interaction in the observed packing arrangement.

The contributions of the central tetraethynylpyrene core and the four 1-adamantyl substituents were also evaluated individually. For this purpose, bonds disconnected for fragment generation were capped with hydrogen atoms in order to preserve closed-shell electronic structures and to obtain chemically meaningful results.

The effect of the rotational offset between adjacent pyrene cores was evaluated by comparing the experimental staggered bilayer core-only model described above (**core (Exp.)** model) with a hypothetical rotationally zero-offset core-only model (**core (ZeroRot)** model) using ALMO-EDA (Table S5). The **core (ZeroRot)** model was constructed from the core-only monomer of the **core (Exp.)** model by copying the monomer and placing the copy 3.297 Å above the first molecule, corresponding to the experimental π–π distance at 100 K. Because the pyrene core lies exactly in the crystallographic *a*–*c* plane, all Cartesian *y* coordinates of the atoms of the core-only monomer were set to 0. The second molecule in the **core (ZeroRot)** model was therefore generated simply by translating the copied molecule by 3.297 Å along the stacking direction, i.e., by adding 3.297 to the Cartesian *y* coordinates.

The **core (ZeroRot)** model gives, as expected, a larger Pauli repulsion term (E_{Pauli} = +363.9 kJ mol^{-1}) than the **core (Exp.)** model (E_{Pauli} = +221.0 kJ mol^{-1}). It also gives stronger attractive electrostatic and dispersion contributions (E_{elec} and E_{disp}) than the **core (Exp.)** model, consistent with its larger direct cofacial overlap. However, the increase in E_{Pauli} surpasses the increases in the absolute values of E_{elec} and E_{disp}. As a result, the total interaction energy of the **core (ZeroRot)** model (E_{total} = −28.9 kJ mol^{-1}) is 63.5 kJ mol^{-1} less stabilizing than that of the **core (Exp.)** model (E_{total} = −92.4 kJ mol^{-1}). These results indicate that the ca. 45° rotational offset also contributes to stabilizing the cofacial pyrene-core contact within the translationally zero-offset π-stack.

Table S5. Energy decomposition analysis of the stacking interaction for experimental staggered bilayer core-only (Exp.) and hypothetical rotationally zero-offset core-only (ZeroRot) models.[a]

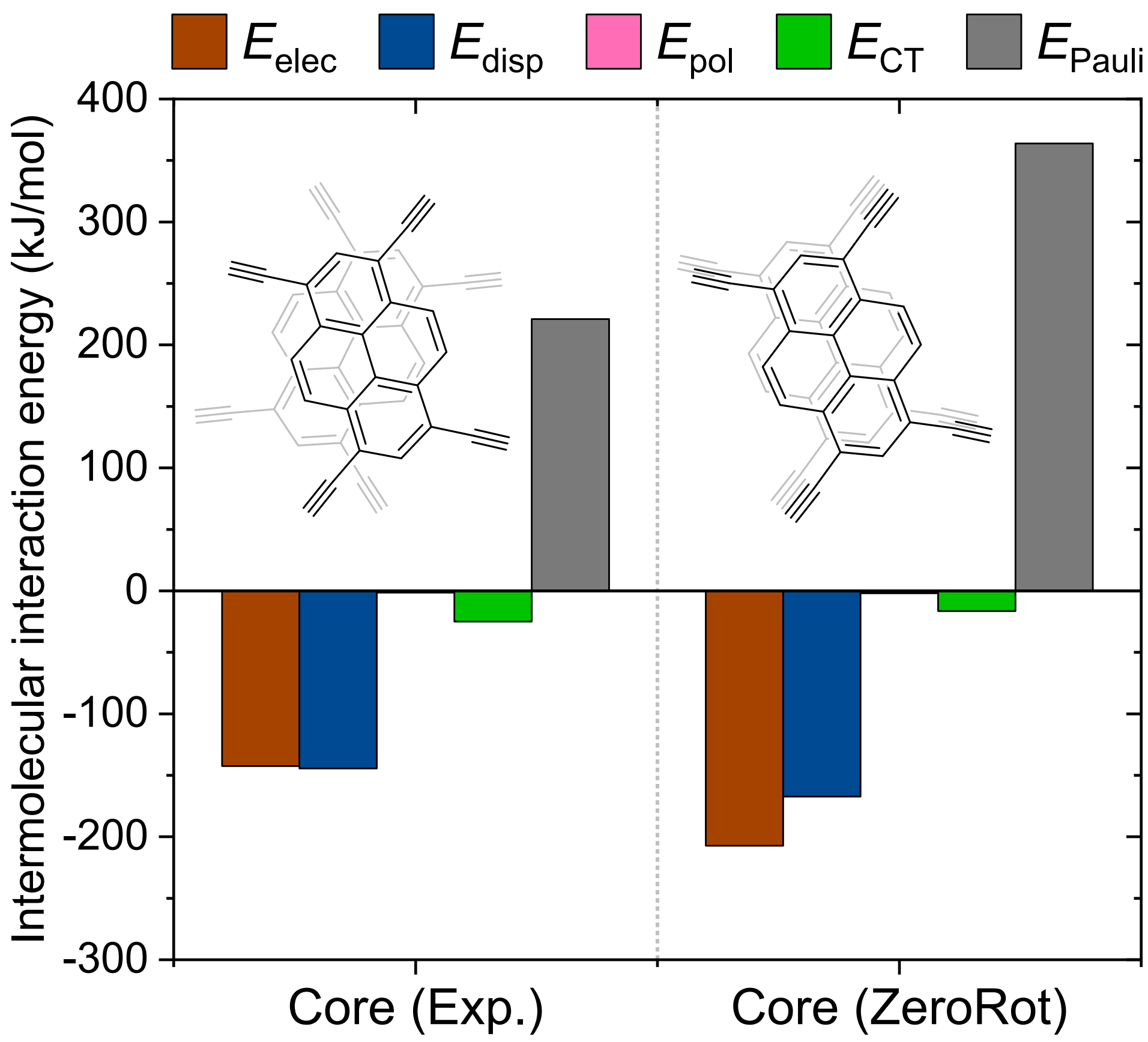

	E_{elec}	E_{disp}	E_{pol}	E_{CT}	E_{Pauli}	E_{total}
Core (Exp.)	−142.5	−144.4	−1.4	−25.0	+221.0	−92.4
Core (ZeroRot)	−207.2	−167.3	−1.9	−16.4	+363.9	−28.9

[a] Energy decomposition analysis was performed using the ALMO-EDA at the B3LYP/6-311G(d) level of theory. **Core (Exp.)**: core-only bilayer extracted from the experimental crystal structure of **CP1**. **Core (ZeroRot)**: hypothetical rotationally zero-offset core-only bilayer constructed from the experimental core geometry at the experimental interplanar distance. E_{elec}: permanent electrostatics, E_{disp}: dispersion, E_{pol}: polarization, E_{CT}: charge transfer, and E_{Pauli}: Pauli repulsion, $E_{total} = E_{elec} + E_{disp} + E_{pol} + E_{CT} + E_{Pauli}$. Energies are reported in kJ mol^{-1}; negative values are stabilizing.

5. Spectroscopic analysis

Monomer spectra in solution

Solution absorption spectra were recorded in CH_2Cl_2 at c(**1**) = 1 × 10^{-5} M and 295 K, whereas photoluminescence and excitation spectra were measured at c(**1**) = 1 × 10^{-7} M and 295 K to minimize aggregation and reabsorption effects. Low-temperature measurements at 77 K were also carried out with a CH_2Cl_2 solution of c(**1**) = 1 × 10^{-7} M to reduce the likelihood of aggregation in the frozen matrix. The sample solution was transferred into an NMR tube, flash-frozen in liquid nitrogen, and measured at 77 K using a cryogenic apparatus. Together with the PMMA data, these solution-state spectra were used to approximate the absorption spectrum of the monomeric species at 100 K.

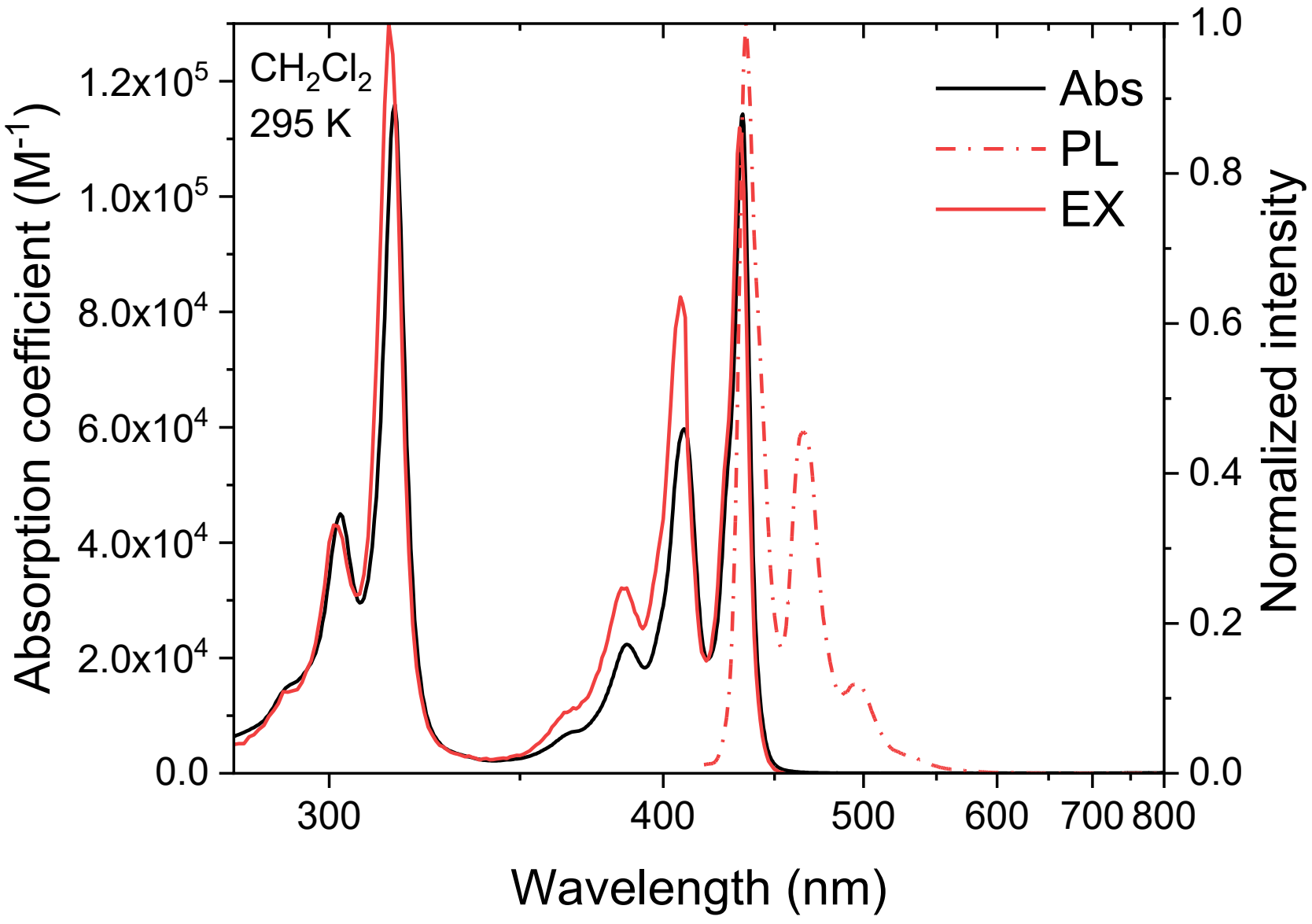

Figure S6. UV–vis absorption (Abs), photoluminescence (PL), and excitation (EX) spectra of **1** in CH_2Cl_2 at 295 K (Abs, black solid line, c = 1.0 × 10^{-5} M, $\lambda_{abs,max}$ = 434 nm; PL, red dashed line, c = 1.0 × 10^{-7} M, excited at λ_{ex} = 407 nm, $\lambda_{em,max}$ = 436 nm; EX, red solid line, c = 1.0 × 10^{-7} M, monitored at λ_{em} = 465 nm, $\lambda_{ex,max}$ = 433 nm).

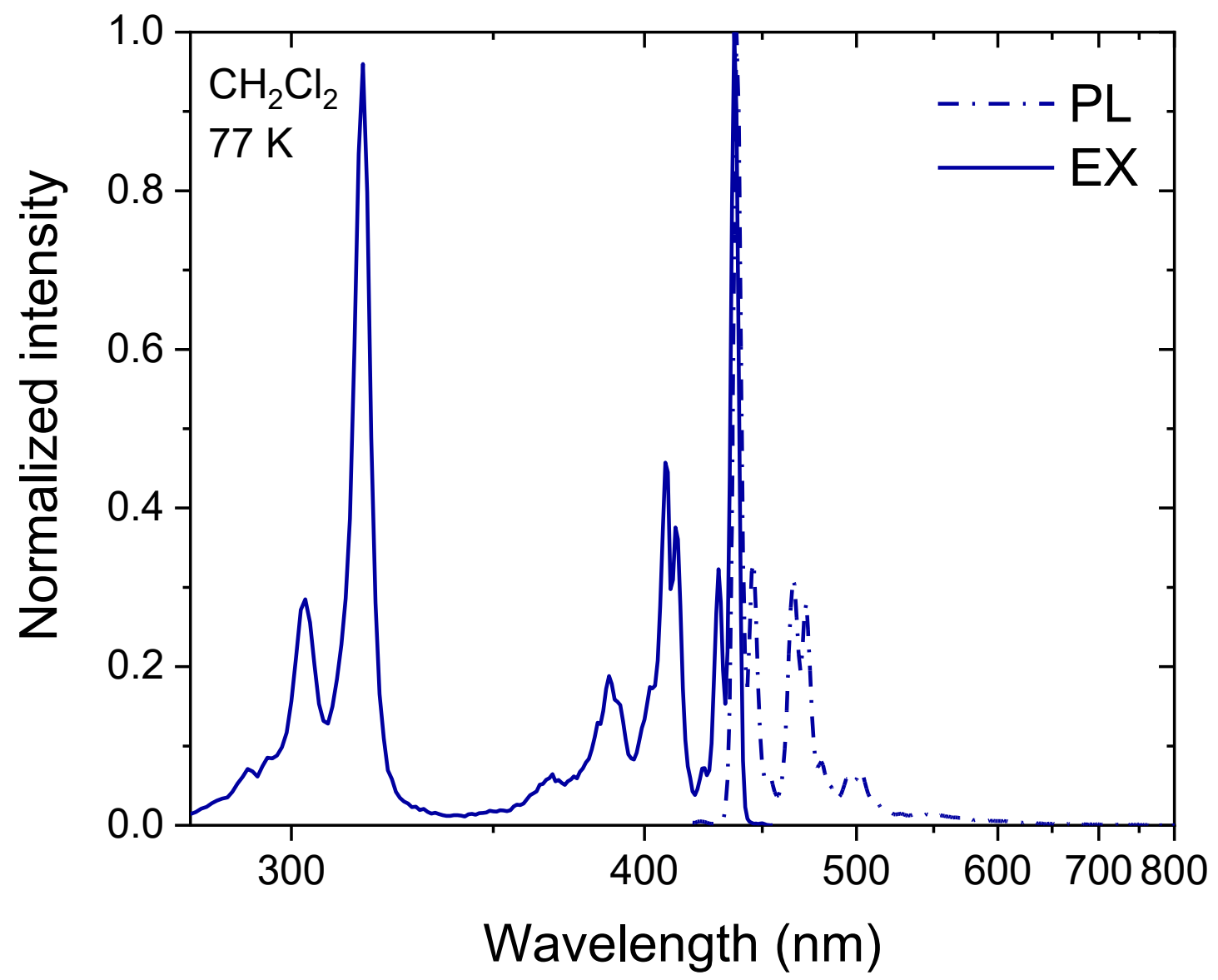

Figure S7. Photoluminescence (PL), and excitation (EX) spectra of **1** in CH_2Cl_2 at 77 K (PL, blue dashed line, c = 1.0 × 10^{-7} M, excited at λ_{ex} = 409 nm, λ_{max} = 438 nm; EX, blue solid line, c = 1.0 × 10^{-7} M, monitored at λ_{em} = 465 nm, λ_{max} = 437 nm).

Isolated monomer in PMMA

PMMA thin films doped with **1** were prepared from a CH_2Cl_2 solution containing 100 mg of PMMA (Mw = 350k) and **1** at 0.01 wt % relative to PMMA in a total volume of 1.0 mL. The homogeneous solution was drop-cast onto a quartz glass substrate and allowed to dry prior to spectroscopic measurements.

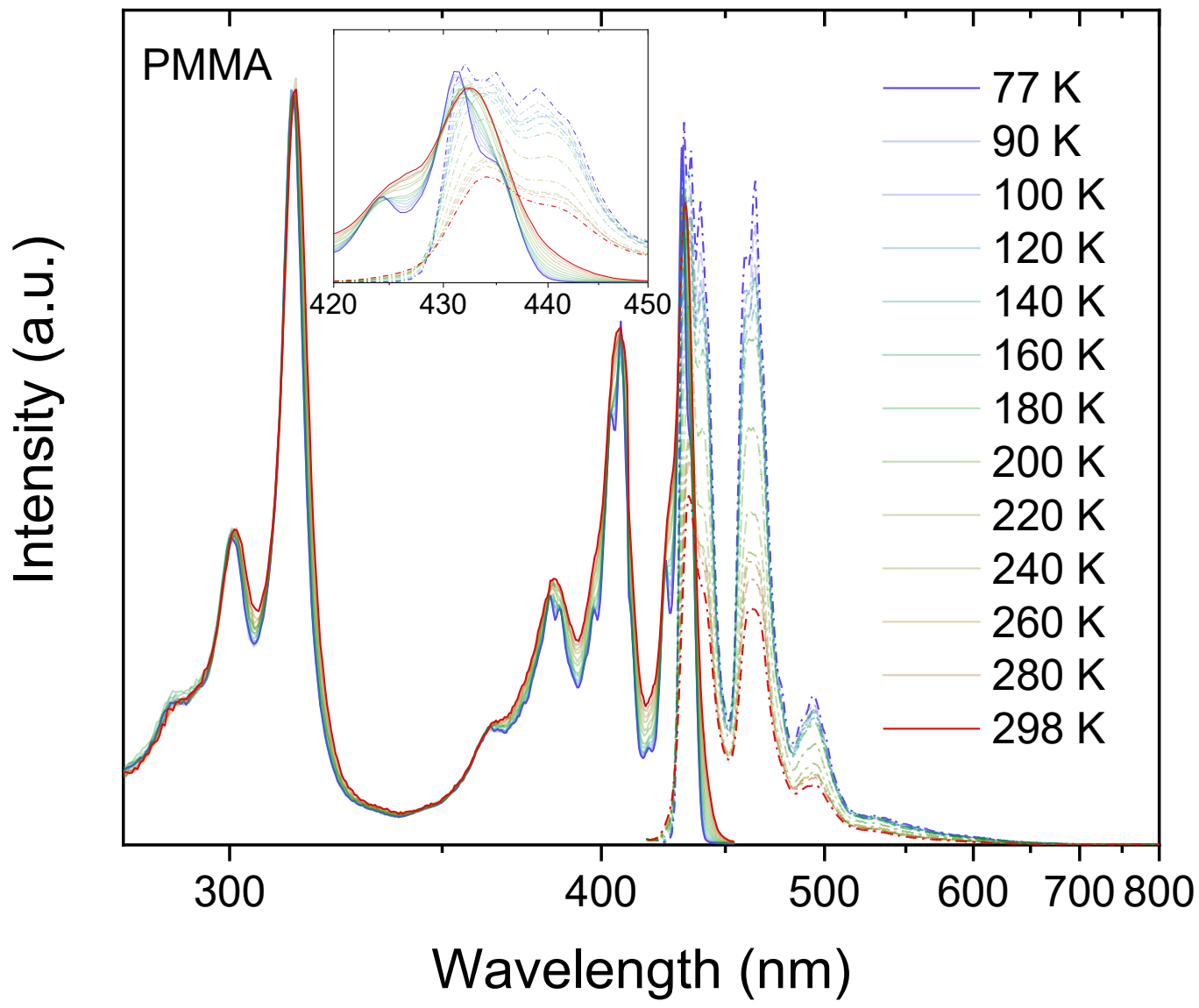

Figure S8. Variable-temperature excitation (EX) and photoluminescence (PL) spectra of **1** in a PMMA thin film on a quartz glass substrate, measured from 77 to 298 K (EX, solid lines, λ_{em} = 464 nm; PL, dashed lines, λ_{ex} = 407 nm). Inset: magnified region between 420 and 450 nm.

Table S6. Peak position data for variable-temperature excitation and photoluminescence spectra of 1 in a PMMA thin film on a quartz glass substrate.

**Temperature** K	**Excitation** nm	**Emission** nm
**77**	431	432
**90**	431	432
**100**	431.5	432
**120**	431.5	432
**140**	431.5	433
**160**	431.5	434
**180**	432	434
**200**	432	434
**220**	432	434
**240**	432.5	434
**260**	432.5	434
**280**	432.5	434
**298**	432.5	434

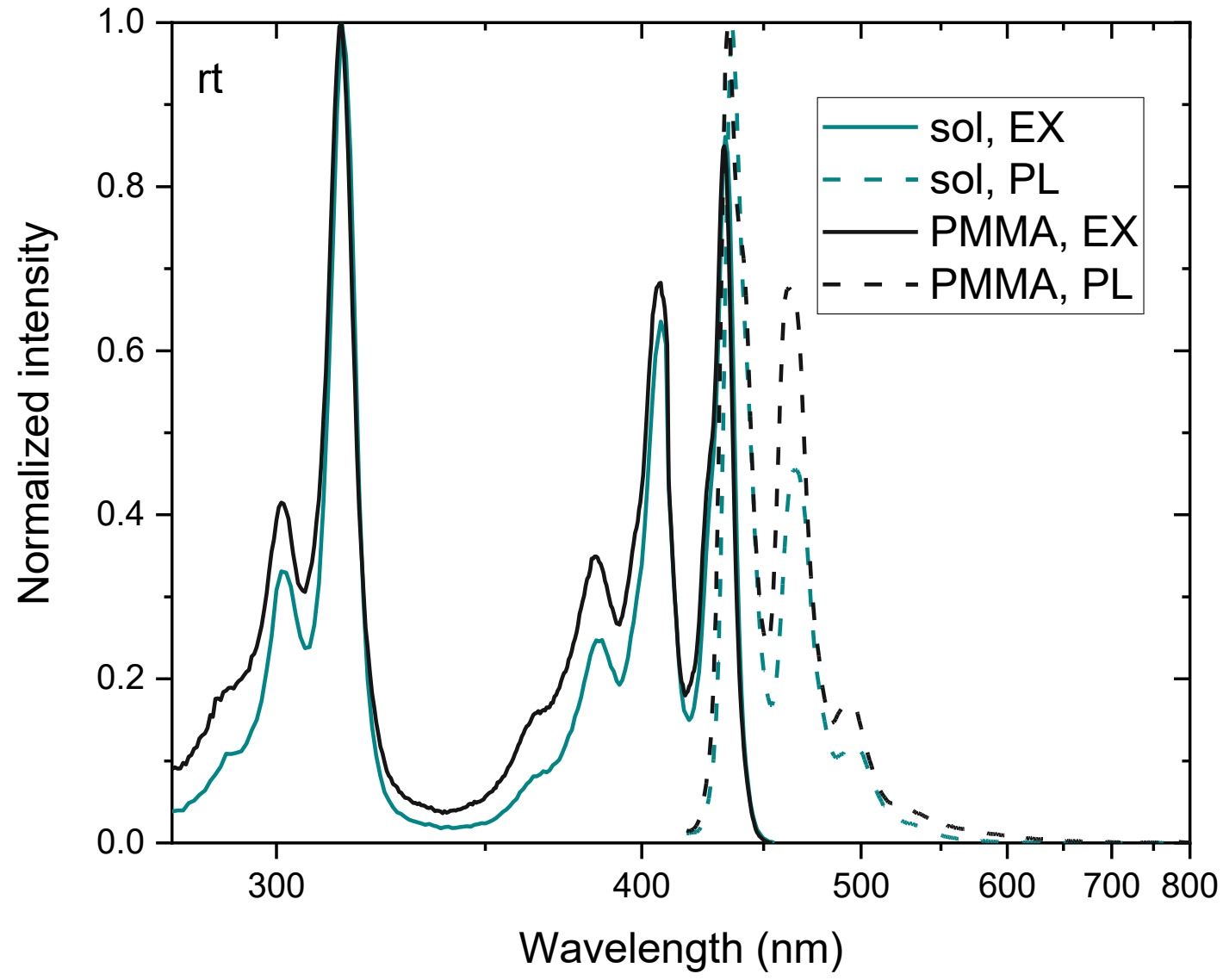

Figure S9. Comparison of the normalized excitation (EX) and photoluminescence (PL) spectra of **1** in CH_2Cl_2 solution and in a PMMA thin film at room temperature (solution EX, cyan solid line, λ_{em} = 465 nm, 295 K; solution PL, cyan dashed line, λ_{ex} = 407 nm, 295 K; PMMA EX, black solid line, λ_{em} = 464 nm, 298 K; PMMA PL, black dashed line, λ_{ex} = 407 nm, 298 K).

The absorption and excitation spectra of **1** in CH_2Cl_2 at 295 K show virtually the same maximum, with $\lambda_{abs,max}$ = 434 nm and $\lambda_{ex,max}$ = 433 nm. A similarly coincident excitation maximum is observed for the PMMA-embedded sample at 298 K ($\lambda_{ex,max}$ = 432.5 nm), and only a very small change is found upon cooling to 77 K ($\lambda_{ex,max}$ = 431 nm). This slight shift is likely not indicative of a substantial change in the electronic transition energy, but rather reflects the enhanced resolution of vibronic structure at lower temperature (see inset in Figure S8). These observations indicate that the excitation spectrum provides a reliable estimate of the absorption spectrum in the present case. They further show that neither transfer from CH_2Cl_2 solution to PMMA nor cooling from 298 to 77 K leads to a significant change in the peak position of the excitation spectrum.

Crystalline samples on wafer

Wafer samples were prepared by transferring several individual crystals of **CP1** onto an Si/SiO_2 wafer using standard crystallographic tools. The crystals had been grown under crystallization conditions analogous to those used for single-crystal X-ray diffraction measurements, and several optically good crystals were selected for transfer onto the wafer. The wafer sample was mounted in the cryostat of the fluorescence spectrometer, purged with argon, and kept under an argon atmosphere throughout the measurement. Spectra were recorded in front-face geometry, with photoluminescence measured at λ_{ex} = 500 nm and excitation spectra recorded by monitoring the emission at λ_{em} = 621–642 nm. After cooling to 77 K by liquid-nitrogen cooling, variable-temperature measurements were carried out upon stepwise heating at 77, 90, and 100 K, followed by 20 K intervals up to 280 K, and a final measurement at 298 K.

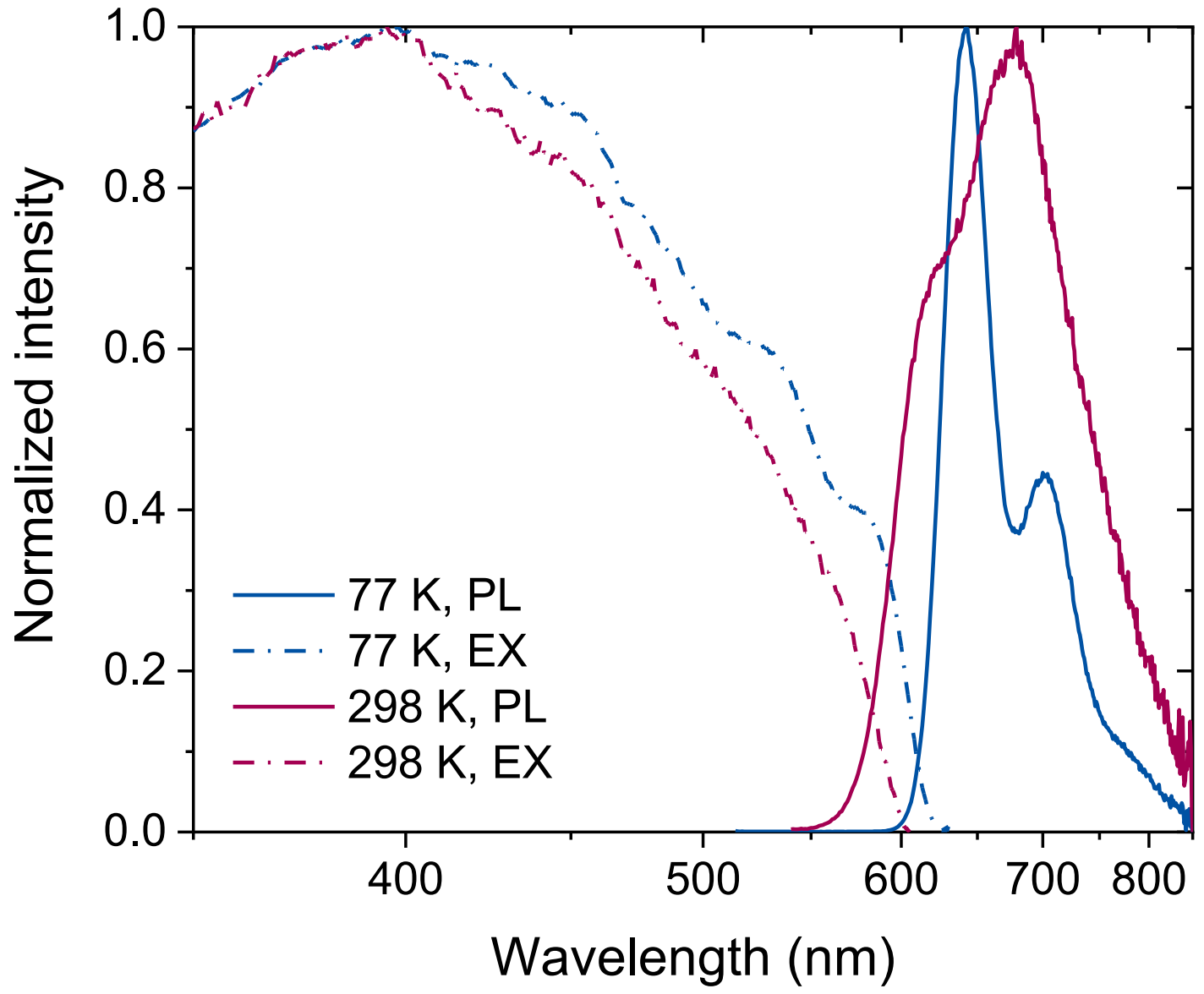

Figure S10. Comparison of the normalized photoluminescence (PL, solid lines) and excitation (EX, dashed lines) spectra of **CP1** recorded at 77 and 298 K. photoluminescence spectra were recorded with λ_{ex} = 500 nm at both temperatures, and excitation spectra were monitored at λ_{em} = 642 nm (77 K) and 621 nm (298 K).

Photoluminescence measurements at 77 K using an NIR detector revealed no detectable emission from **CP1** at wavelengths above 800 nm, indicating the absence of detectable NIR phosphorescence under these conditions (Figure S11). A previously reported series of multi-alkynylated pyrenes (ref. S15) showed that increasing the number of alkynyl substituents resulted in low intersystem crossing efficiencies. A similar effect may operate in compound **1** and contribute to the suppression of phosphorescence from **CP1**.

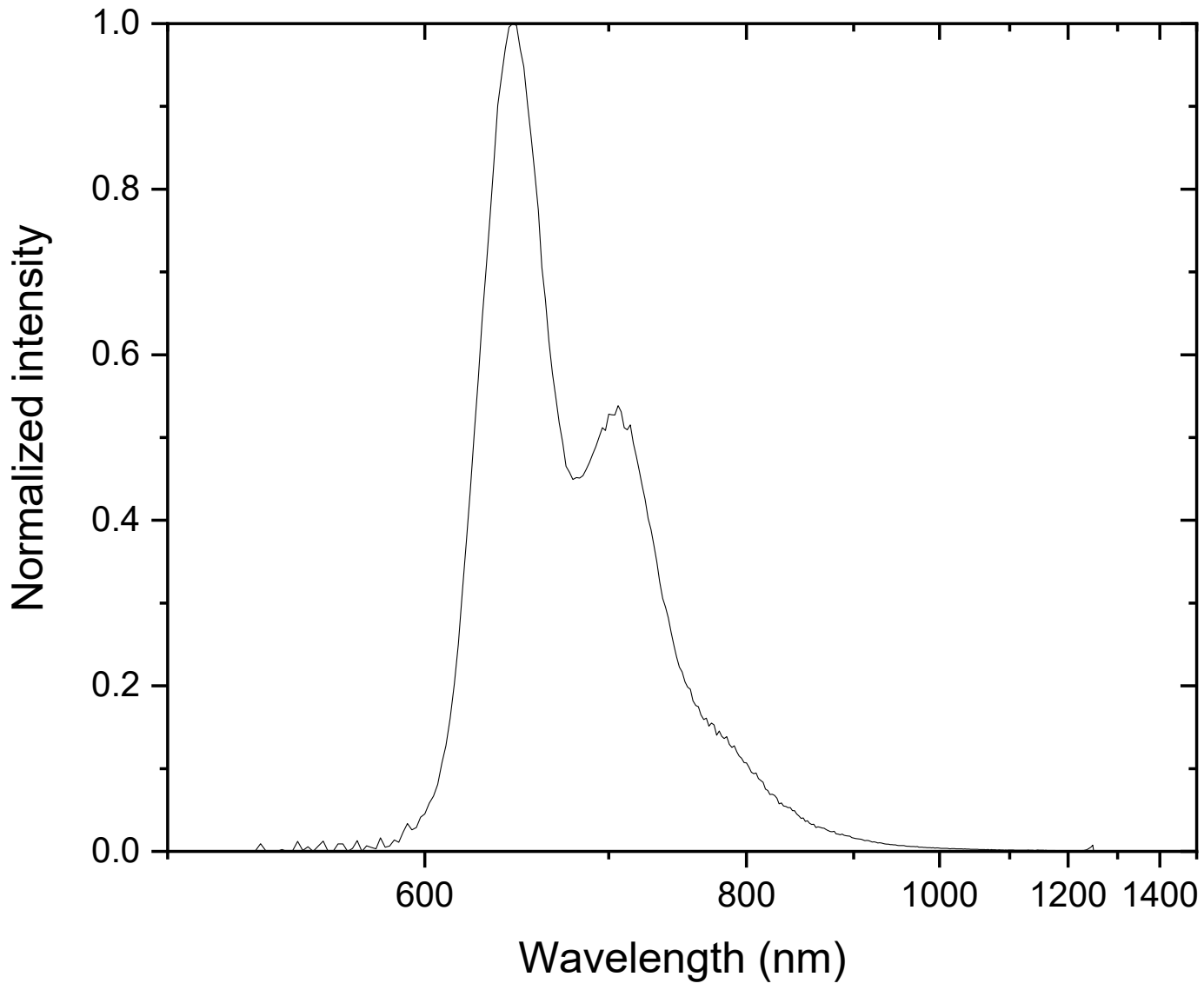

Figure S11. Photoluminescence spectrum of **CP1** recorded at 77 K using an NIR detector. λ_{ex} = 500 nm.

To verify that the excitation spectrum of the crystal ensemble on an Si/SiO_2 wafer can be used as a practical proxy for the crystal absorption spectrum, diffuse-reflectance data recorded at room temperature were additionally analyzed by the Kubelka–Munk method (Figure S12). In this approach, the reflectance R of an optically thick scattering sample is converted into the Kubelka–Munk function, $F(R) = (1 - R)^2/(2R)$, which is commonly used as an approximate quantity proportional to the absorption coefficient. The excitation spectrum was used in the present study because it could be recorded conveniently for wafer samples over a wide temperature range, whereas the room-temperature Kubelka–Munk spectrum served here as an independent reference for the solid-state absorption profile. The broadly similar spectral shapes of the room-temperature Kubelka–Munk and excitation spectra support that the ensemble excitation spectrum provides a reasonable approximation of the solid-state absorption behavior of **CP1** and can therefore be used to follow its temperature dependence.

Absorption profiles of the microcrystalline samples were obtained from diffuse-reflectance measurements of $BaSO_4$-triturated samples using a PerkinElmer Lambda 950 UV/vis/NIR spectrometer equipped with an integrating sphere. The measured reflectance spectra were converted into Kubelka–Munk spectra according to $F(R) = (1 - R)^2/(2R)$, where R is the diffuse reflectance. The resulting $F(R)$ values were used as an approximate measure of the solid-state absorption profile.

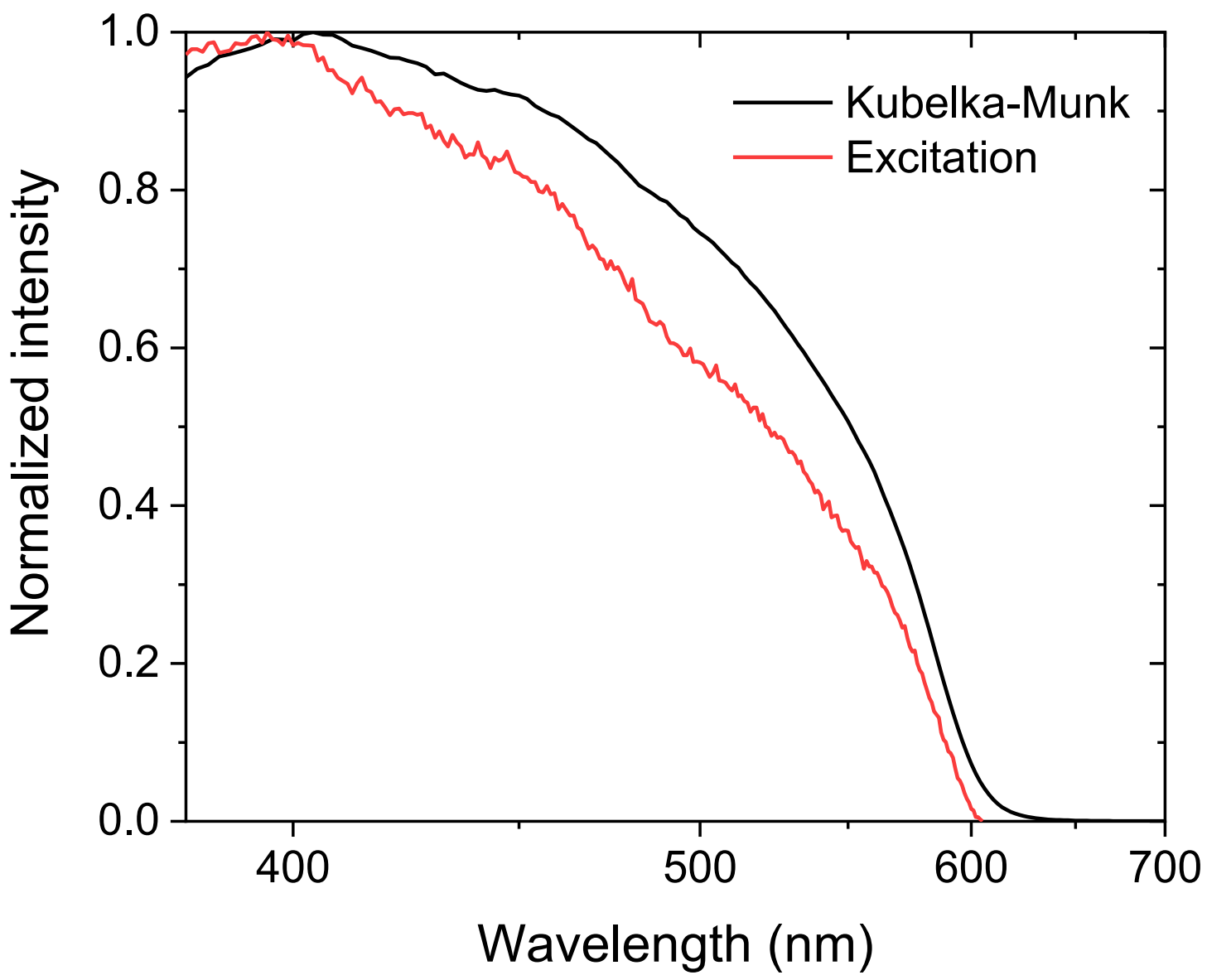

Figure S12. Comparison of the room-temperature Kubelka–Munk spectrum derived from diffuse-reflectance measurements and the room-temperature excitation spectrum of a wafer sample containing an ensemble of **CP1**. For excitation spectrum λ_{em} = 642 nm.

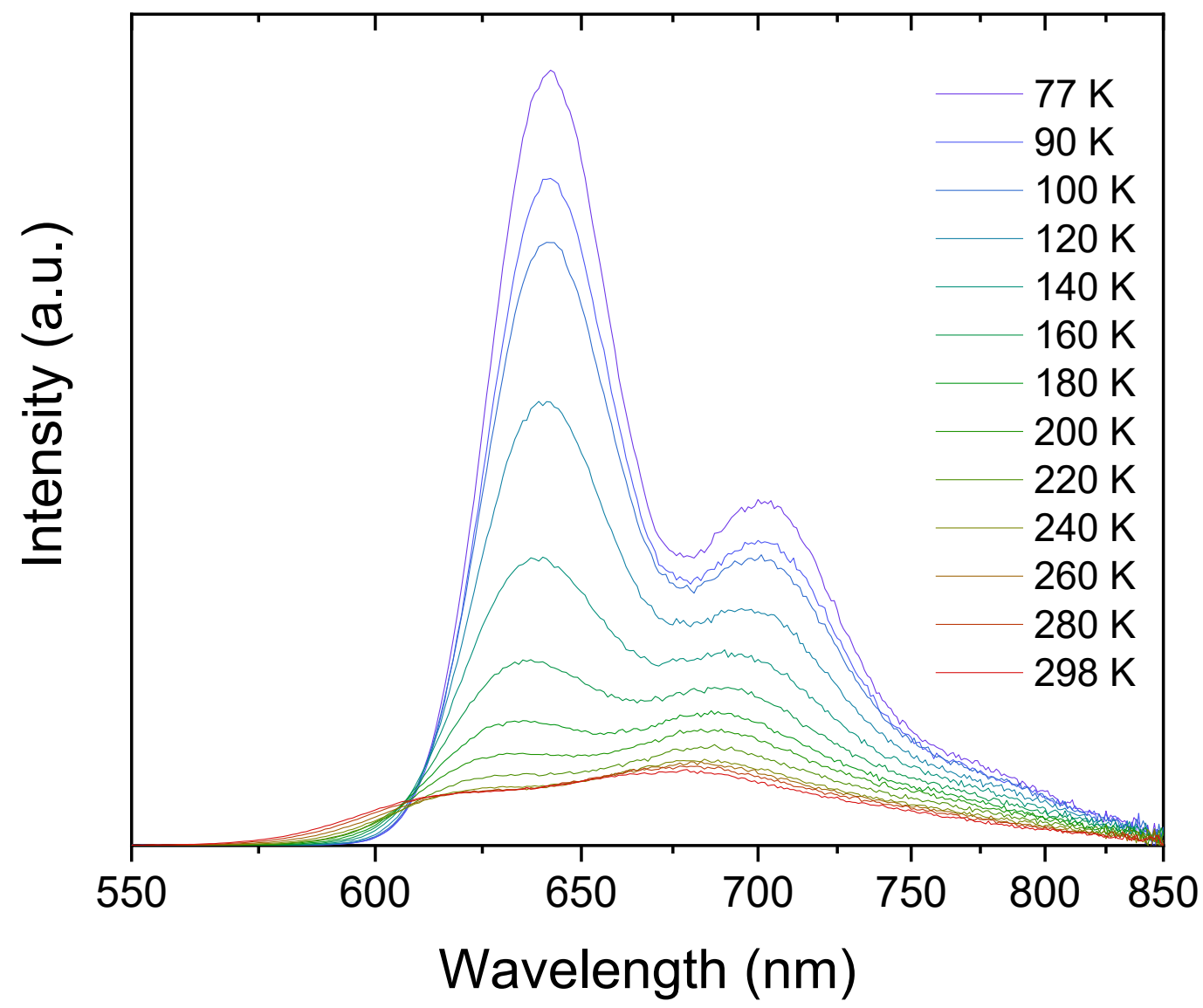

Figure S13. Variable-temperature photoluminescence spectra of an ensemble wafer sample of **CP1**, measured in front-face geometry from 77 to 298 K at λ_{ex} = 500 nm.

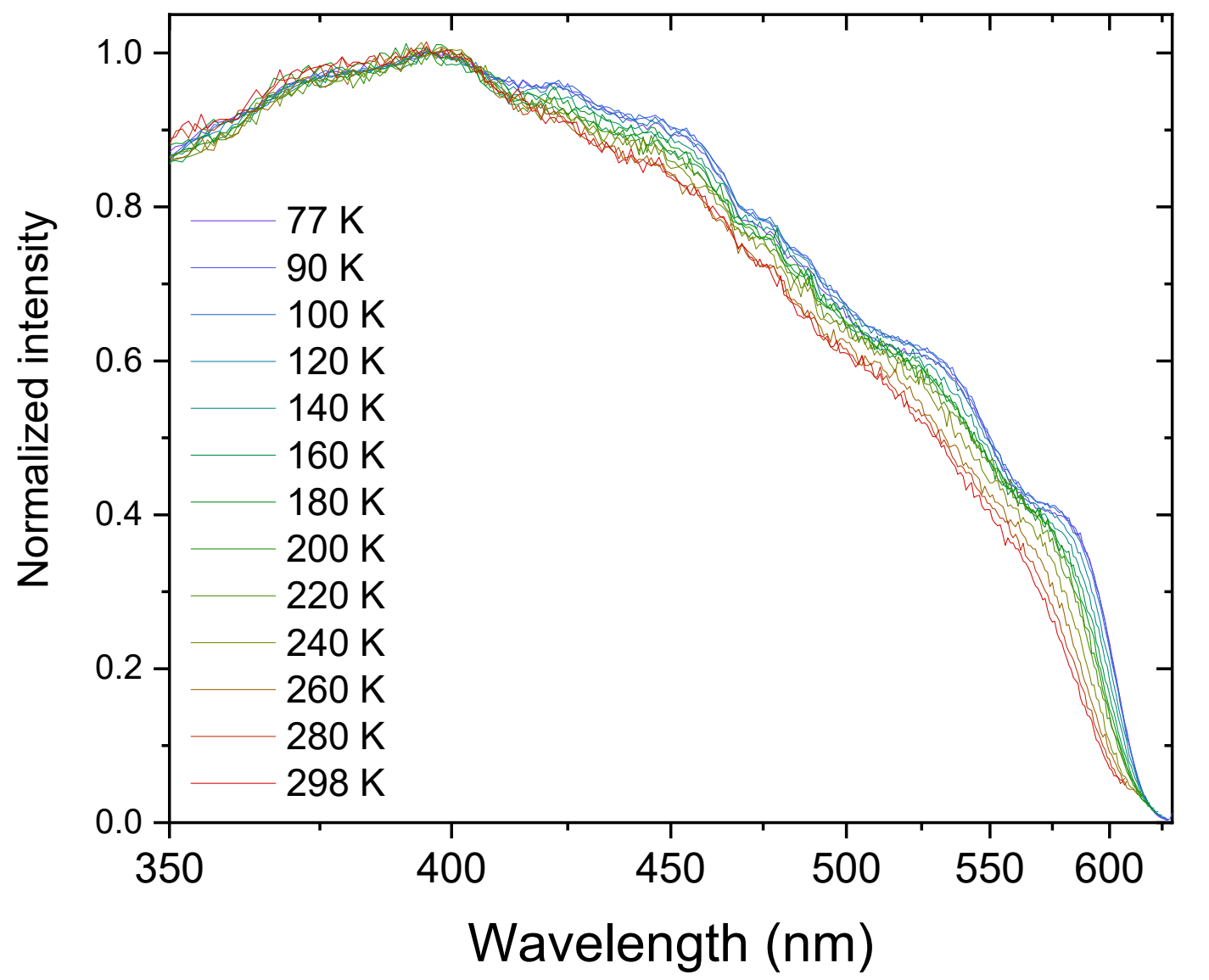

Figure S14. Variable-temperature excitation spectra of a wafer sample containing an ensemble of **CP1**, measured in front-face geometry from 77 to 298 K by monitoring the emission at λ_{em} = 621–642 nm (at each temperature, the excitation spectrum was recorded at the emission maximum of the first photoluminescence peak).

Fitting details

Emission and excitation spectra recorded in the range 77–298 K were analyzed by nonlinear least-squares fitting using the Nonlinear Curve Fit routine implemented in OriginPro (OriginLab Corporation) with the Levenberg–Marquardt algorithm. In all cases, the wavelength axis (λ, nm) was converted to wavenumber according to $\tilde{\nu} = 10^7/\lambda$, with $\tilde{\nu}$ given in cm^{-1}, in order to use a linear energy scale, since wavelength is not proportional to energy whereas wavenumber is. Parameter uncertainties were taken as standard errors derived from the covariance matrix of the fit.

For the temperature-dependent emission spectra (514–850 nm), the spectral profiles were modeled as the sum of three Gaussian bands in the wavenumber domain and a quadratic baseline according to

$$f(\tilde{\nu}) = A_1 \exp\left[-\frac{(\tilde{\nu}-m_1)^2}{2s_1^2}\right] + A_2 \exp\left[-\frac{(\tilde{\nu}-m_2)^2}{2s_2^2}\right] + A_3 \exp\left[-\frac{(\tilde{\nu}-m_3)^2}{2s_3^2}\right] + a\tilde{\nu}^2 + b\tilde{\nu} + c \qquad \text{eq. 1}$$

where A_i, m_i, and s_i denote the amplitude, band center, and standard deviation of the *i*-th Gaussian component, respectively, and *a*, *b*, and *c* describe the quadratic baseline. For the fitting analysis, the baseline was expressed in terms of the centered coordinate $u = \tilde{\nu} - 18000$ instead of $\tilde{\nu}$ in order to improve numerical stability and reduce parameter correlation in the polynomial baseline expression. Here, 18000 cm^{-1} corresponds to 556 nm and lies near the middle of the fitted spectral range. All emission spectra were fitted simultaneously using the Global Fit mode in OriginPro, but no parameter was treated as shared across the temperature series; thus, all parameters were refined independently for each dataset, i.e., for each temperature. Initial values were estimated from the experimental spectra, and the optimized parameters from one dataset were used as initial values for the subsequent dataset to facilitate convergence.

For the temperature-dependent excitation spectra (479–627 nm), the spectral profiles were modeled as the sum of two Gaussian bands and a quadratic baseline according to

$$f(\tilde{\nu}) = A_1 \exp\left[-\frac{(\tilde{\nu}-m_1)^2}{2s_1^2}\right] + A_2 \exp\left[-\frac{(\tilde{\nu}-m_2)^2}{2s_2^2}\right] + a\tilde{\nu}^2 + b\tilde{\nu} + c \qquad \text{eq. 2}$$

The parameter definitions are analogous to those used for the emission fitting. Because the second Gaussian component was weak and could not always be refined robustly for individual spectra, its amplitude A_2 was treated as a shared parameter across all datasets. In addition, the quadratic baseline coefficient *a* was also treated as a shared parameter, whereas all remaining parameters were refined independently for each dataset.

For the excitation spectra, the peaks were clearly recognizable at lower temperatures, whereas at higher temperatures the spectral features became progressively broader and less distinct, rendering direct peak assignment increasingly ambiguous. Therefore, the fitting procedure described above was necessary to assign the peak positions reliably.

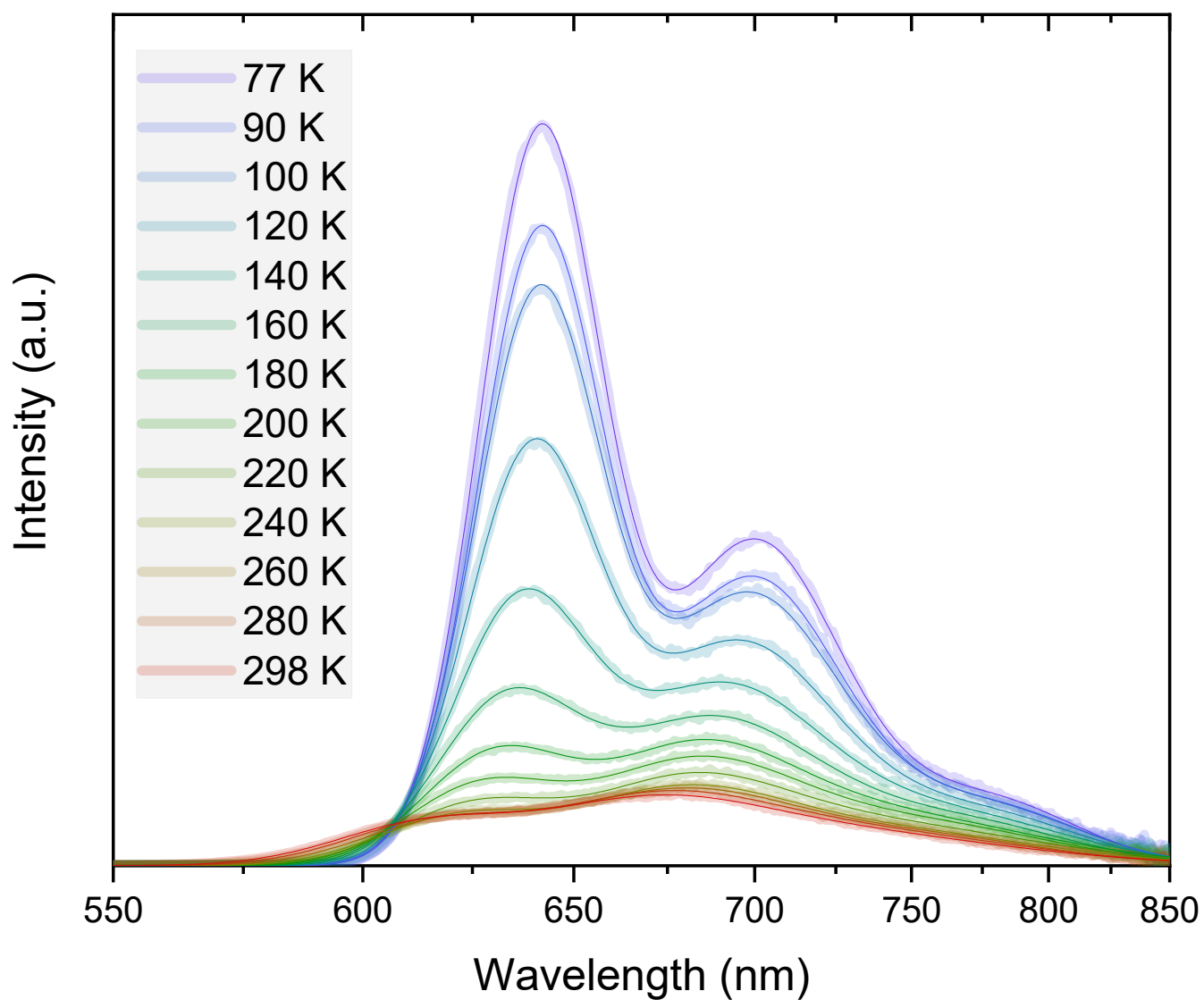

Figure S15. Temperature-dependent emission spectra of an ensemble of **CP1** recorded between 77 and 298 K. The experimentally measured spectra are displayed as thick semi-transparent lines, whereas the corresponding fitted curves are shown as thin solid lines.

Table S7. Fitted parameters for the emission spectra of the ensemble of CP1 between 77 K and 298 K (Figure S15) using eq. 1.[a]

**Temperature**	**0–0 (m_1)**	**0–1 (m_2)**	**Ratio of peaks $I_{0\text{-}0}/I_{0\text{-}1}$ (A_1/A_2)**
K	nm	nm	-
**77**	641.5	699.9	1.57
**90**	641.4	699.2	1.50
**100**	640.9	698.1	1.44
**120**	639.3	695.3	1.23
**140**	636.3	689.8	1.00
**160**	632.5	687.0	0.67
**180**	629.7	681.5	0.83
**200**	626.1	681.7	0.52
**220**	624.8	678.9	0.77
**240**	620.5	675.9	0.52
**260**	617.0	677.2	0.36
**280**	614.6	670.5	0.51
**298**	613.0	667.9	0.75

[a] 0–0: peak position of the first photoluminescence peak (highest energy peak), 0–1: peak position of the second photoluminescence peak (second highest energy peak), $I_{0\text{–}0}/I_{0\text{–}1}$: ratio of the intensity of the first photoluminescence peak ($I_{0\text{–}0}$) and second photoluminescence peak ($I_{0\text{–}1}$).

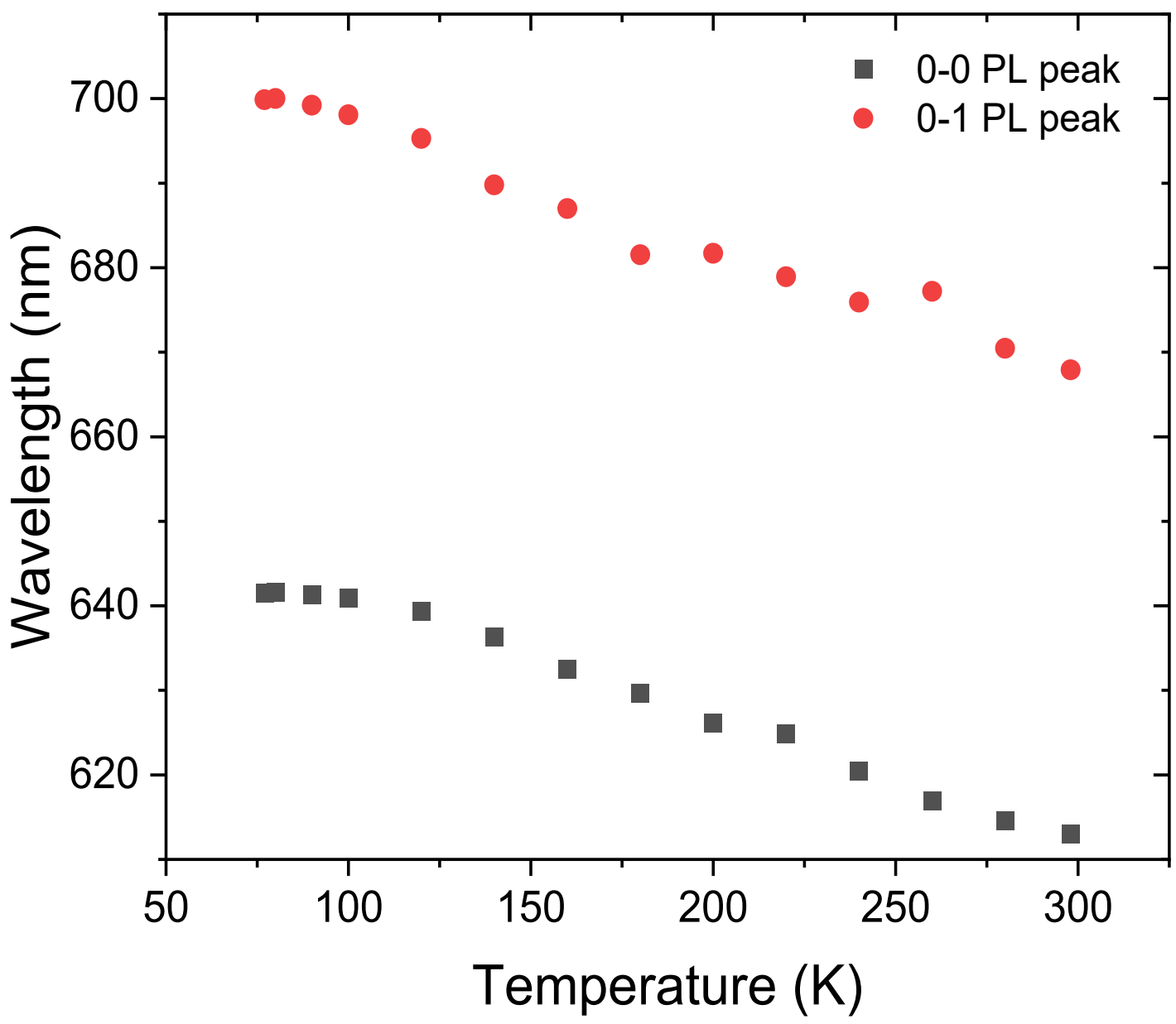

Figure S16. Temperature dependence of the photoluminescence (PL) peak wavelengths assigned to the 0–0 and 0–1 vibronic emission bands fitted according to eq. 1.

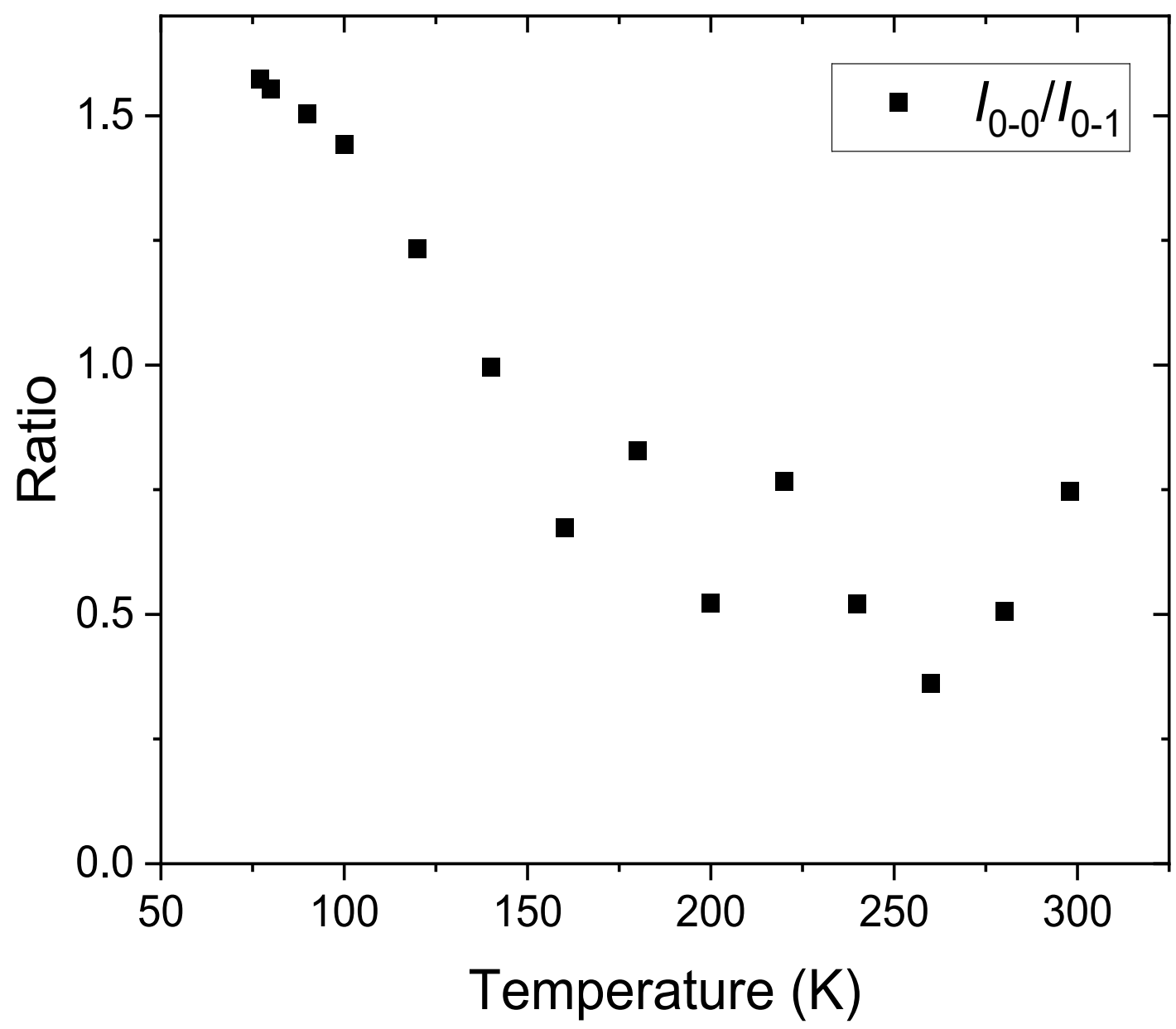

Figure S17. Temperature dependence of the intensity ratio between the 0–0 and 0–1 photoluminescence emission bands, I_{0-0}/I_{0-1}.

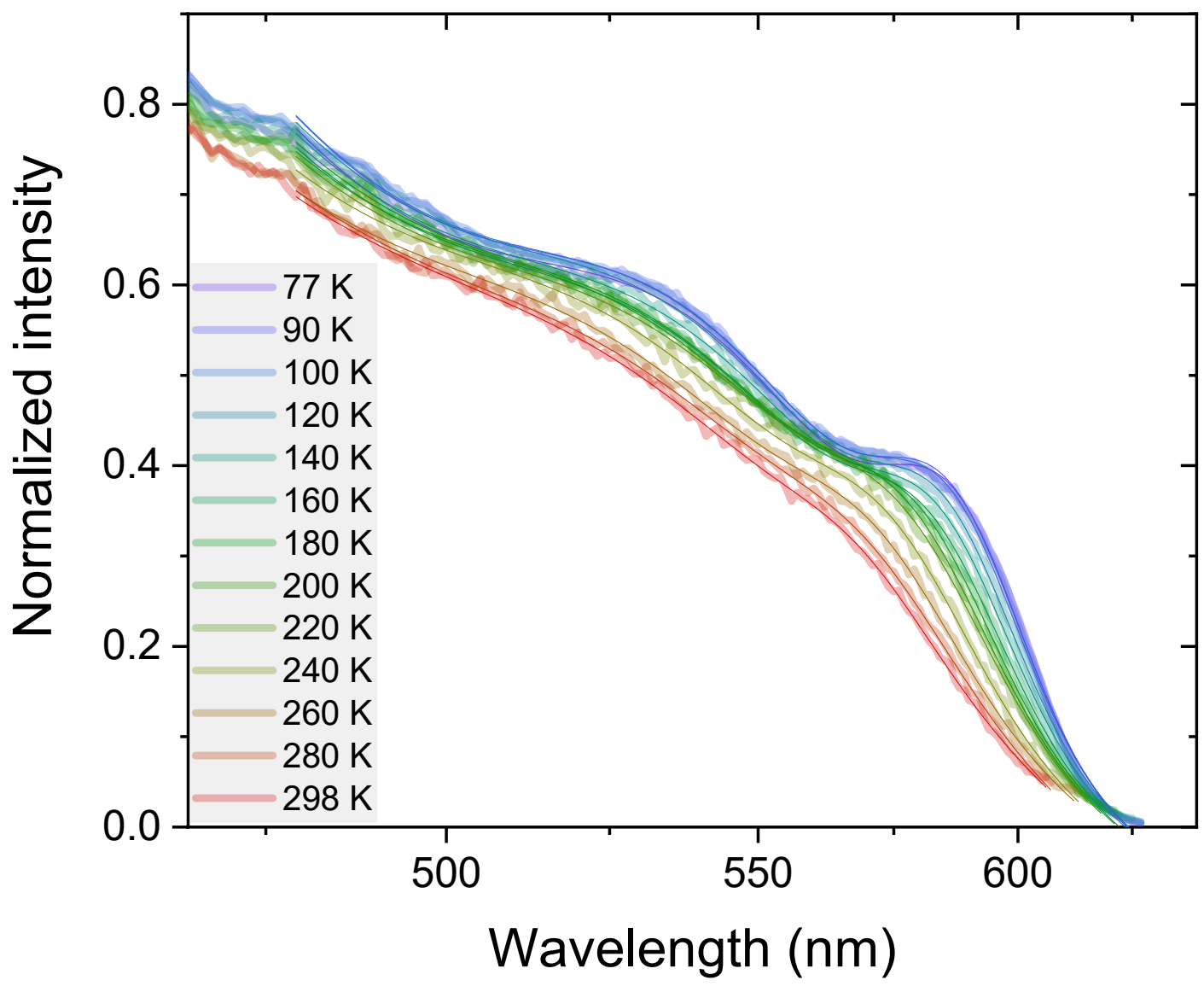

Figure S18. Temperature-dependent excitation spectra of an ensemble of **CP1** recorded between 77 and 298 K. The experimentally measured spectra are displayed as thick semi-transparent lines, whereas the corresponding fitted curves are shown as thin solid lines.

Table S8. Fitted parameters for the excitation spectra of the ensemble of CP1 between 77 K and 298 K (Figure S18) using eq. 2.[a]

**Temperature**	**A_{0-0} (m_1)**	**A_{0-1} (m_2)**
K	nm	nm
**77**	587.8	542.4
**90**	587.4	542.1
**100**	587.0	542.2
**120**	586.0	541.9
**140**	585.0	542.1
**160**	583.2	541.2
**180**	581.4	541.1
**200**	579.6	539.2
**220**	578.9	539.9
**240**	575.9	536.8
**260**	573.1	535.2
**280**	571.7	537.2
**298**	569.9	535.7

[a] A_{0-0}: fitted peak position of the first excitation peak, used here as estimated first absorption peak. A_{0-1}: the corresponding value for the second excitation peak.

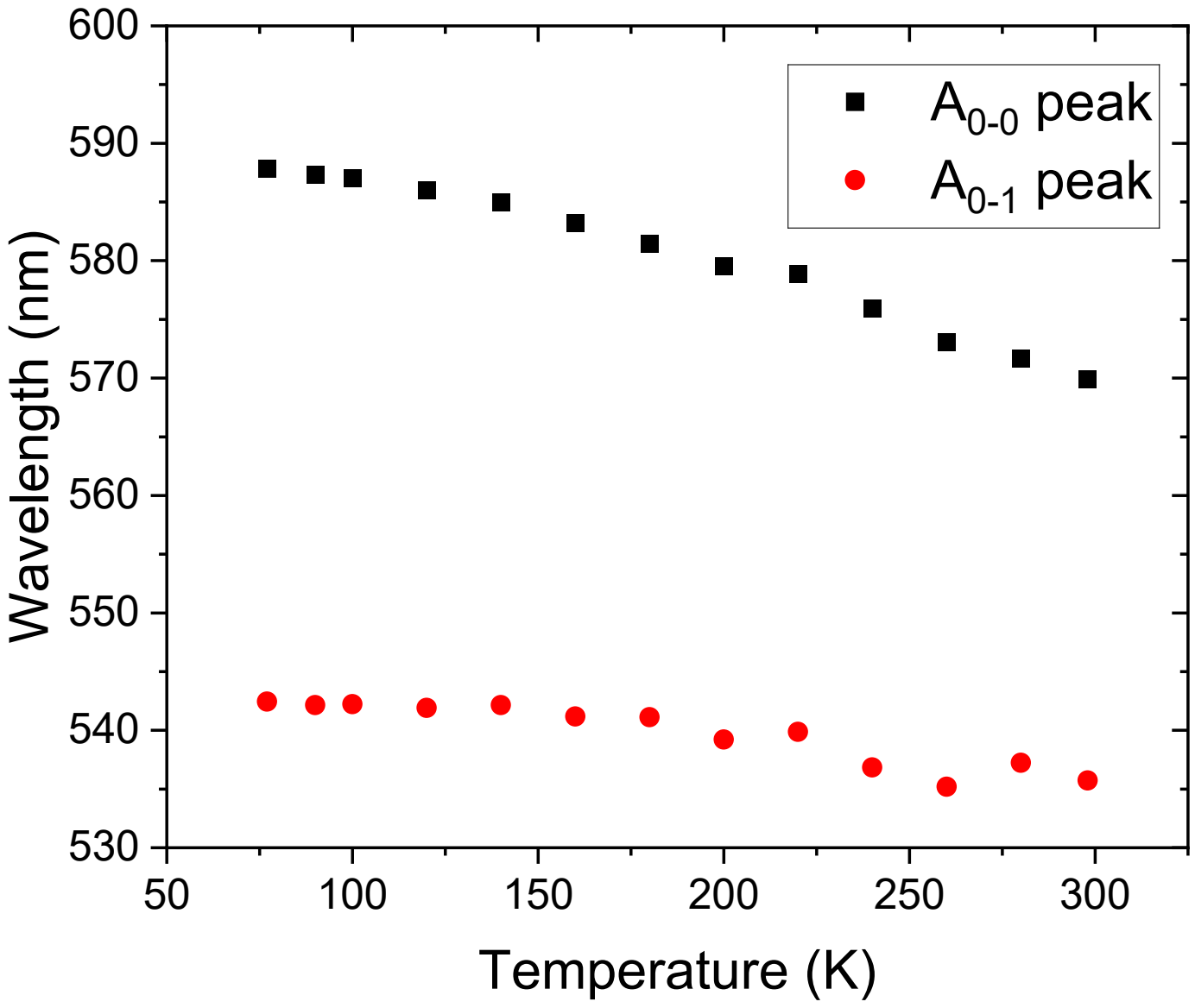

Figure S19. Temperature dependence of the excitation (EX) peak wavelengths assigned to the A_{0-0} and A_{0-1} vibronic bands fitted according to eq. 2.

Table S9. Temperature dependence of the estimated solution-to-crystal shifts (ΔE_{obs}) and corresponding photoluminescence shifts (ΔE_{PL}).[a]

T K	$E_{A_{0-0,crystal}}$ eV	$E_{A_{0-0,monomer}}$ eV	ΔE_{obs} eV	$E_{0-0_{crystal}}$ eV	$E_{0-0_{monomer}}$ eV	ΔE_{PL} eV
**77**	2.109	2.877	−0.768	1.933	2.870	−0.937
**90**	2.111	2.877	−0.766	1.933	2.870	−0.937
**100**	2.112	2.874	−0.761	1.935	2.870	−0.936
**120**	2.116	2.874	−0.758	1.940	2.870	−0.931
**140**	2.120	2.874	−0.754	1.949	2.864	−0.915
**160**	2.126	2.874	−0.747	1.960	2.857	−0.897
**180**	2.133	2.870	−0.738	1.969	2.857	−0.888
**200**	2.140	2.870	−0.731	1.980	2.857	−0.877
**220**	2.142	2.870	−0.728	1.985	2.857	−0.873
**240**	2.153	2.867	−0.714	1.999	2.857	−0.859
**260**	2.164	2.867	−0.703	2.010	2.857	−0.847
**280**	2.169	2.867	−0.698	2.018	2.857	−0.840
**298**	2.176	2.867	−0.691	2.023	2.857	−0.834

[a] T: temperature (K). $E_{A_{0-0,crystal}}$: estimated energy of the 0–0 absorption peak of **CP1** from the lowest energy excitation band of an ensemble of **CP1**; $E_{A_{0-0,monomer}}$: estimated energy of the 0–0 absorption peak of isolated molecules of **1** from the lowest energy excitation band of **1** embedded in a PMMA matrix on quartz glass substrate; ΔE_{obs}: solution-to-crystal shift defined as $E_{A_{0-0,crystal}} - E_{A_{0-0,monomer}}$; $E_{0-0_{crystal}}$: energy of the 0–0 photoluminescence peak of **CP1** measured on an ensemble of **CP1**; $E_{0-0_{monomer}}$: energy of the 0–0 photoluminescence peak of isolated molecules of **1** measured on **1** embedded in a PMMA matrix on quartz glass substrate; ΔE_{PL}: solution-to-crystal photoluminescence shift defined as $E_{0-0_{crystal}} - E_{0-0_{monomer}}$. All energies are given in eV and temperatures in K.

6. Exciton coupling

The excitonic contribution was evaluated by considering the two most fundamental components of intermolecular exciton coupling, namely Coulombic coupling and charge-transfer-(CT)-mediated coupling. These terms were first determined for a molecular dimer and subsequently extended to an infinite one-dimensional stack within a tight-binding framework in order to approximate the polymeric π-stacks of **1** investigated in this study. The values discussed in this section are summarized in Table S10.

Coulombic exciton coupling

Coulombic (long-range) Frenkel exciton couplings, J_{Coul}, between neighboring molecules were evaluated with the transition charges from electrostatic potentials (TrESP) method.[S16] First, the monomer $S_0 \rightarrow S_1$ transition density (or transition charges) was obtained at the B3LYP/def2svp level on the crystal-derived monomer geometry. Transition charges were then fitted to reproduce the electrostatic potential of the transition density and subsequently used to compute the intermolecular Coulomb interaction energy for each dimer in the crystal geometry:

$$J_{\mathrm{Coul}} = \sum_{A \in 1} \sum_{B \in 2} \frac{q_A^{\mathrm{tr}} q_B^{\mathrm{tr}}}{4\pi\varepsilon_0 R_{AB}}$$

, where q_A^{tr} and q_B^{tr} are the fitted transition charges and R_{AB} are interatomic distances between atoms A and B on different monomers. TrESP fitting and coupling evaluations were carried out with Multiwfn.[S17]

The sign of the Coulombic coupling in this system was examined with particular care because the overall sign of a separately calculated monomer transition density is known to depend on the arbitrary phase chosen in the excited-state calculation.[S18] The present symmetry discussion refers only to the π-core, because the adamantyl substituents make only a negligible contribution to the $S_0 \rightarrow S_1$ transition density and are additionally disordered in the crystal structure along the π-stacking direction. On this basis, each monomer in the π-stacked dimer was treated as having D_{2h} symmetry. By contrast, the fitted transition-charge pattern for the $S_0 \rightarrow S_1$ transition does not retain this high symmetry and is numerically asymmetric; however, because the charges are fitted onto atoms that lie exactly in one plane, the remaining formal geometrical symmetry is C_s, with the mirror plane coinciding with the π-surface. In the crystal structure, two neighboring molecules form a strictly parallel π-stack oriented exactly along the crystallographic two-fold axis, and the center of each monomer lies on an inversion center (Figure S5). As a result, a separately calculated monomer can formally yield two possible transition-charge patterns with opposite overall dipolar directions, depending on how the molecular axis is defined in the calculation, although both correspond to the same physical transition. This can in turn lead to a TrESP-derived J_{Coul} value with the opposite sign for a given dimer. Therefore, the physically meaningful sign of the Coulombic coupling was assigned from the relative crystal geometry, i.e., as the H-type sign expected for the strictly parallel arrangement.

The resulting Coulombic couplings correspond to vacuum values. In molecular crystals, dielectric screening can modify these values, with deviations from the vacuum limit reported to range from −20% to +10%, depending on the type of intermolecular displacement, even for an optical dielectric constant of $\varepsilon = 2$.[S16] In the present study, this effect was disregarded. In addition, transition dipole moments calculated at the TD-DFT/B3LYP level are known to be overestimated by a factor of approximately 1.3. Because Coulombic coupling scales with the product of the transition dipole moments of the two interacting monomers, the calculated J_{Coul} values were uniformly rescaled by a factor of $1/(1.3)^2$.[S16] All J_{Coul} values reported in this work refer to these rescaled quantities.

CT-mediated exciton coupling

The CT-mediated exciton coupling (J_{CT}) and site-energy correction (Δ_{CT}) were evaluated within the framework of second-order perturbation theory, treating the intermolecular charge-transfer (CT) states

as virtual excited configurations that mix with the local Frenkel excitation.[S19] This treatment is valid under the condition that the electronic coupling matrix elements, given by the electron and hole transfer integrals t_e and t_h (vide infra), are small compared to the energy separation between the Frenkel and CT states, i.e. $|t_e|,|t_h| << |E_{CT} - E_{S1}|$.

The energy of the bright local excitation, E_{S1}, was determined by TD-DFT at the B3LYP/def2svp level on the crystal-derived monomer geometry. The CT-state energy, E_{CT}, was estimated using constrained DFT (cDFT) at the ωB97XD/def2svp level of theory on the corresponding dimer geometry using QChem program package.[S14] In this procedure, the energy of the neutral dimer $M^{(0)}M^{(0)}$ was first calculated without charge constraints, followed by a calculation of the charge-separated configuration $M^{+}M^{-}$, in which one molecule carries a positive charge and the other a negative charge. The resulting energy difference defines the diabatic CT-state energy relevant for Frenkel–CT mixing.

Within second-order perturbation theory, the CT-mediated exciton coupling between two equivalent Frenkel excitations is given by

$$J_{\mathrm{CT}} = -2\frac{t_{\mathrm{e}}\, t_{\mathrm{h}}}{E_{\mathrm{CT}} - E_{\mathrm{S}_1}}$$

. The factor of 2 originates from two symmetry-equivalent virtual CT pathways contributing to exciton coupling, namely the $t_e \rightarrow t_h$ and $t_h \rightarrow t_e$ processes (electron transfer followed by hole transfer and vice versa). The overall minus sign follows the sign convention used by Spano[S19] and ensures consistency between the signs of transfer integrals, band dispersion, and exciton coupling.

In addition to exciton coupling, Frenkel–CT mixing gives rise to a second-order site-energy correction to the local excitation energy. In the polymeric (one-dimensional stack) limit, where each molecule interacts with two equivalent nearest neighbors (left and right), this correction is given by

$$\varDelta_{\mathrm{CT}} = -2\frac{t_{\mathrm{e}}^2 + t_{\mathrm{h}}^2}{E_{\mathrm{CT}} - E_{\mathrm{S}_1}}$$

. Here, the factor of 2 reflects contributions from virtual CT recombination processes involving both neighboring molecules. In contrast, for an isolated dimer only one neighbor contributes, and the corresponding site-energy correction would lack this factor.

The total CT-mediated contribution to the optical energy shift (ΔE_{CT}) is then given by

$$\Delta E_{\mathrm{CT}} = J_{\mathrm{CT}} + \varDelta_{\mathrm{CT}}$$

Thus, the CT-mediated shift of the optical transition energy depends on both the excitonic splitting term, J_{CT}, and the site-energy correction, $\varDelta_{CT}$, and is therefore sensitive to both the magnitudes and the relative signs of the electron and hole transfer integrals.

Calculation of charge transfer integrals

Charge-transfer (CT) integrals for hole and electron transport, t_h and t_e, were calculated using density functional theory as implemented in the ADF (Amsterdam Density Functional) software package.[S20] All calculations employed the PW91 exchange–correlation functional together with a TZP (triple-ζ plus polarization) basis set. Dimer geometries were constructed directly from the experimental crystal structure, and no geometry optimization was performed in order to preserve the solid-state packing arrangement.

The electronic couplings were evaluated using the fragment-orbital (dimer projection) approach. Monomer frontier orbitals (HOMO for hole transport and LUMO for electron transport) were first calculated for the isolated monomers in the geometries they adopt within the dimer. These orbitals were subsequently used as a localized basis to construct the dimer Hamiltonian and overlap matrices. Effective transfer integrals (t_x, where x = e for electron transfer or h for hole transfer) were obtained from

the off-diagonal Hamiltonian and overlap elements, corrected for non-orthogonality by Löwdin orthogonalization, according to

$$t_x = \frac{H_{12} - S_{12}(H_{11} + H_{22})/2}{1 - S_{12}^2}$$

, where H_{ij} and S_{ij} denote the Hamiltonian and overlap matrix elements between fragment orbitals *i* and *j*. The signs of t_e and t_h were assigned following the convention used by Gisslén and Scholz[S21] as described below

Sign convention for transfer integrals

The signs of the transfer integrals were assigned within the dimer frontier-orbital picture following the convention of Gisslén and Scholz.[S21] Here, the off-diagonal coupling elements of the dimer Hamiltonian, t_L and t_H (transfer parameters), were used to define the signs of the transfer integrals. When the dimer LUMO orbital corresponds to the in-phase combination of the two monomer orbitals, that is, the same orbital phase on both monomers, the corresponding transfer parameter is negative ($t_L < 0$). When the orbital phases on the two monomers are opposite, that is, out-of-phase, the corresponding transfer parameter is positive ($t_L > 0$). For the dimer HOMO orbital, the opposite applies, that is, for an in-phase combination $t_H > 0$, whereas for an out-of-phase combination $t_H < 0$. The sign of the electron transfer integral (t_e) was taken directly from the LUMO transfer parameter, that is, $t_e = t_L$, whereas the hole transfer integral (t_h) was defined with the opposite sign relative to the HOMO transfer parameter, $t_h = -t_H$, because hole transfer describes the motion of an electron vacancy rather than that of an electron itself.

With this convention, the character of the CT-mediated excitonic coupling can be assigned from the relationship between the HOMO–HOMO and LUMO–LUMO phase patterns across the dimer, that is, whether the two orbital pairs show the same or opposite phase relationships. If the HOMO–HOMO phases are opposite whereas the LUMO–LUMO phases are the same, the two orbital pairs show opposite phase relationships. Under the convention described above, this gives $t_H < 0$ and thus $t_h > 0$, while $t_L < 0$ and thus $t_e < 0$, so that t_h and t_e have opposite signs, consistent with H-type CT-mediated coupling. This case applies to the current system and is illustrated visually in a later section (Figure S21). By contrast, if the HOMO–HOMO and LUMO–LUMO phase relationships are the same, that is, either both in phase or both out of phase, this leads to the same signs of t_h and t_e, and thus to J-type CT-mediated coupling.

This sign definition is convention-based and should not be interpreted as an absolute sign assignment independent of orbital phase choice. As emphasized by Hestand and Spano,[S19] a different but internally consistent phase convention can flip the individual signs of t_e and t_h without changing the underlying photophysics. What is physically meaningful is the consistent relative phase relationship within a given system.

Combined exciton coupling[S3]

Following the treatment of Spano and co-workers,[S19] the overall excitonic contribution in the one-dimensional stack was described within an effective Frenkel Hamiltonian, in which the nearest-neighbor intermolecular coupling is written as the sum of the Coulombic and CT-mediated terms. In addition, virtual Frenkel-CT mixing gives rise to a site-energy correction, Δ_{CT}, which is negative when $E_{CT} > E_{S1}$ and therefore produces a uniform red shift of all excitonic states. For an infinite one-dimensional stack with nearest-neighbor interactions, the exciton dispersion is thus written as

$$E(k) = E_{S_1} + \Delta_{\mathrm{CT}} + 2(J_{\mathrm{Coul}} + J_{\mathrm{CT}})\cos(k)$$

Consequently, the crystallization-induced shift of the optically allowed band-edge state is determined by the combined effects of the Coulombic coupling, the CT-mediated coupling, and the CT-induced site-

energy correction. In the notation used here, the corresponding excitonic contribution, with the formal signs of all terms retained, is defined as

$$\Delta E_{\mathrm{exci}}^{\mathrm{formal}} = \Delta_{\mathrm{CT}} + 2(J_{\mathrm{Coul}} + J_{\mathrm{CT}})$$

We refer to this as the "formal" value to distinguish it from the maximal possible red shift discussed below. Its sign and magnitude are governed by the balance between the long-range Coulombic interaction and the short-range CT-mediated contributions. For comparison, we also evaluated a quantity defined as

$$\Delta E_{\mathrm{exci}} = \Delta_{\mathrm{CT}} - 2(|J_{\mathrm{Coul}}| + |J_{\mathrm{CT}}|)$$

in which both J_{Coul} and J_{CT} are treated as negative irrespective of their formal sign, that is, as if both contributions acted in a red-shifting manner.[S3] This quantity is used here as an estimate of the maximal red shift that may become spectroscopically observable when the formally lowest-energy transition is indirect or symmetry-forbidden, but acquires partial intensity in the solid state through vibronic coupling, static disorder, phonon-assisted relaxation, or other symmetry-breaking effects.

The calculated $\Delta E_{\mathrm{exci}}^{\mathrm{formal}}$ values (Table S10) are slightly positive over the entire temperature range and therefore do not account for the experimentally observed bathochromic shifts, which are negative. Thus, within the formal sign convention of the combined excitonic model, exciton coupling does not explain the pronounced red-shift observed for the polymeric π-stacks of **1** in **CP1**. Even when ΔE_{exci}, which represents an upper-bound red-shift estimate obtained by treating all excitonic contributions as red-shifting, is considered the resulting values remain far smaller in magnitude than the experimentally observed shifts and do not approach the values below −0.7 eV. These results indicate that excitonic effects alone are insufficient to explain the optical red shift in the crystal and that additional contributions, most notably band-like frontier crystal orbital (FCO) interactions, must dominate the observed solution-to-crystal shift.

Table S10. Temperature-dependent excitonic coupling parameters for the polymeric π-stacks of 1 in CP1 using geometries obtained from variable temperature single crystal X-ray diffraction analysis.[a]

T	J_{Coul}	E_{CT}	E_{S1}	$E_{CT}-E_{S1}$	t_h	t_e	Δ_{CT}	J_{CT}	ΔE_{exci}^{formal}	ΔE_{exci}
K	eV	eV	eV	eV	eV	eV	eV	eV	eV	eV
**90**	+0.048	3.805	2.889	0.916	+0.163	−0.231	−0.174	+0.082	+0.086	−0.435
**100**	+0.044	3.777	2.883	0.894	+0.157	−0.232	−0.175	+0.081	+0.075	−0.426
**120**	+0.040	3.794	2.874	0.919	+0.148	−0.227	−0.159	+0.073	+0.067	−0.386
**140**	+0.042	3.800	2.891	0.909	+0.146	−0.226	−0.159	+0.072	+0.070	−0.387
**160**	+0.040	3.805	2.869	0.936	+0.142	−0.222	−0.149	+0.067	+0.067	−0.365
**180**	+0.041	3.783	2.844	0.938	+0.140	−0.220	−0.145	+0.066	+0.068	−0.358
**200**	+0.038	3.845	2.891	0.954	+0.139	−0.220	−0.142	+0.064	+0.063	−0.347
**220**	+0.036	3.891	2.904	0.987	+0.133	−0.216	−0.131	+0.058	+0.058	−0.319
**240**	+0.036	3.880	2.891	0.989	+0.132	−0.214	−0.128	+0.057	+0.058	−0.315
**260**	+0.034	3.865	2.878	0.988	+0.128	−0.207	−0.119	+0.053	+0.055	−0.294
**280**	+0.036	3.913	2.927	0.986	+0.124	−0.204	−0.116	+0.051	+0.059	−0.290
**298**	+0.029	3.982	2.951	1.031	+0.116	−0.199	−0.103	+0.045	+0.044	−0.250

[a] T: temperature (K). J_{Coul}: Long-range Coulombic exciton coupling calculated using the TrESP method at the B3LYP/def2svp level of theory and uniformly rescaled by a factor of $1/(1.3)^2$ to account for the overestimation by DFT calculations.[S16] E_{CT}: Charge-transfer state energy estimated as the energy difference between the neutral dimer $M^{(0)}M^{(0)}$ and the charge-separated dimer M^+M^-, calculated by constrained DFT at the ωB97XD/def2svp level of theory. E_{S1}: Energy of the lowest bright singlet excited state of the monomer calculated by TD-DFT at the B3LYP/def2svp level of theory. $E_{CT} - E_{S1}$: Estimated CT state energy level against the local Frenkel excitation. Δ_{CT}: Site-energy correction arising from Frenkel–CT mixing in the polymeric one-dimensional limit, calculated as $\Delta_{CT} = -2(t_e^2 + t_h^2)/(E_{CT} - E_{S1})$. J_{CT}: CT-mediated exciton coupling calculated as $J_{CT} = -2t_e t_h/(E_{CT} - E_{S1})$, where the prefactor 2 arises from two symmetry-equivalent electron–hole transfer pathways. t_e, t_h: Electron and hole transfer integrals between neighboring molecules calculated at the PW91/TZP level of theory using crystal-derived dimer geometries. Signs are assigned according to conventions used by Scholz et al.[S21] ΔE_{exci}^{formal}: Overall CT-mediated and Coulombic excitonic contribution including the site-energy correction Δ_{CT}, with signs preserved (allowed transitions), defined as $\Delta E_{exci}^{formal} = \Delta_{CT} + 2(J_{CT} + J_{Coul})$. ΔE_{exci}: Upper red-shift limit of CT-mediated and Coulombic excitonic interactions, including the site-energy correction Δ_{CT}, defined as $\Delta E_{exci} = \Delta_{CT} - 2(|J_{CT}|+|J_{Coul}|)$.

7. Frontier crystal orbitals (FCO)[S3]

Overview of frontier molecular orbital interactions in an organic crystal

Molecular crystals of PAHs exhibit intermolecular orbital interactions that may extend over multiple molecules within a π-stack. In an ideal band-structure framework, such as that discussed for crystalline solids by Hoffmann,[S22] these interactions are generally described under the assumption of perfect translational periodicity and full coherence throughout the crystal. This limiting picture is useful for defining the maximum band-like effect of intermolecular orbital overlap, but it does not fully reflect molecular crystals composed of neutral, apolar organic molecules such as PAHs, where structural disorder, thermal motion, and weak intermolecular interactions limit the spatial extent of coherent orbital interactions. We therefore use the term frontier crystal orbitals (FCOs) to describe frontier-orbital interactions that are band-like in origin but finite in spatial coherence.[S3]

In the following analysis, the ideal fully coherent FCO contribution (ΔE_{FCO}) is first evaluated. In this limiting case, the FCO contribution is equivalent to the ideal band-structure contribution (ΔE_{band}), that is, $\Delta E_{\mathrm{FCO}} = \Delta E_{\mathrm{band}}$. The effective coherence length N_{coh} is then estimated by assuming that the experimentally observed solution-to-crystal shift is dominated by the FCO contribution (ΔE_{FCO}) together with the excitonic contribution (ΔE_{exci}). This approximation is appropriate for the present rigid, apolar **1**/**CP1** system, for which other effects, such as strong dielectric stabilization or large polarity-induced solvatochromic contributions, are not expected to be dominant. The same effective coherence length is applied to both the FCO and excitonic contributions because both terms depend on intermolecular orbital overlap within the π-stack and are therefore expected to operate over a similar coherence scale. This procedure allows the ideal fully coherent FCO and excitonic contributions to be converted into effective, coherence-length-scaled contributions.

This band-like but spatially limited FCO contribution was evaluated using a one-dimensional tight-binding model, taking advantage of the one-dimensional π-stacking character of **CP1**. As illustrated in in Figure S20, overlap between neighboring HOMOs and LUMOs gives rise to dispersive valence- and conduction-band-like states, here referred to as FCOs. Within a nearest-neighbor tight-binding framework, the dispersion of these states is governed by the intermolecular orbital interactions, which may be quantified, among other ways, by the transfer integrals t_h and t_e. The signs of these integrals, which arise from the phases of the molecular orbitals, together with their magnitudes, determine the curvature of the HOMO- and LUMO-derived bands and thereby the location of the band extrema in *k*-space. When t_e and t_h, defined according to the above sign convention, have the same sign, the system corresponds to a J-type orbital interaction, whereas opposite signs correspond to an H-type orbital interaction. In both cases, the band-edge gap of the periodic stack is given by the energetic separation between the upper edge of the HOMO-derived FCO band and the lower edge of the LUMO-derived FCO band, and may therefore differ substantially from the monomeric HOMO-LUMO gap. The resulting FCO contribution to the solution-to-crystal shift is accordingly governed by the extent to which intermolecular orbital interactions modify the frontier-orbital gap in the crystal, which in the present case leads to gap narrowing.

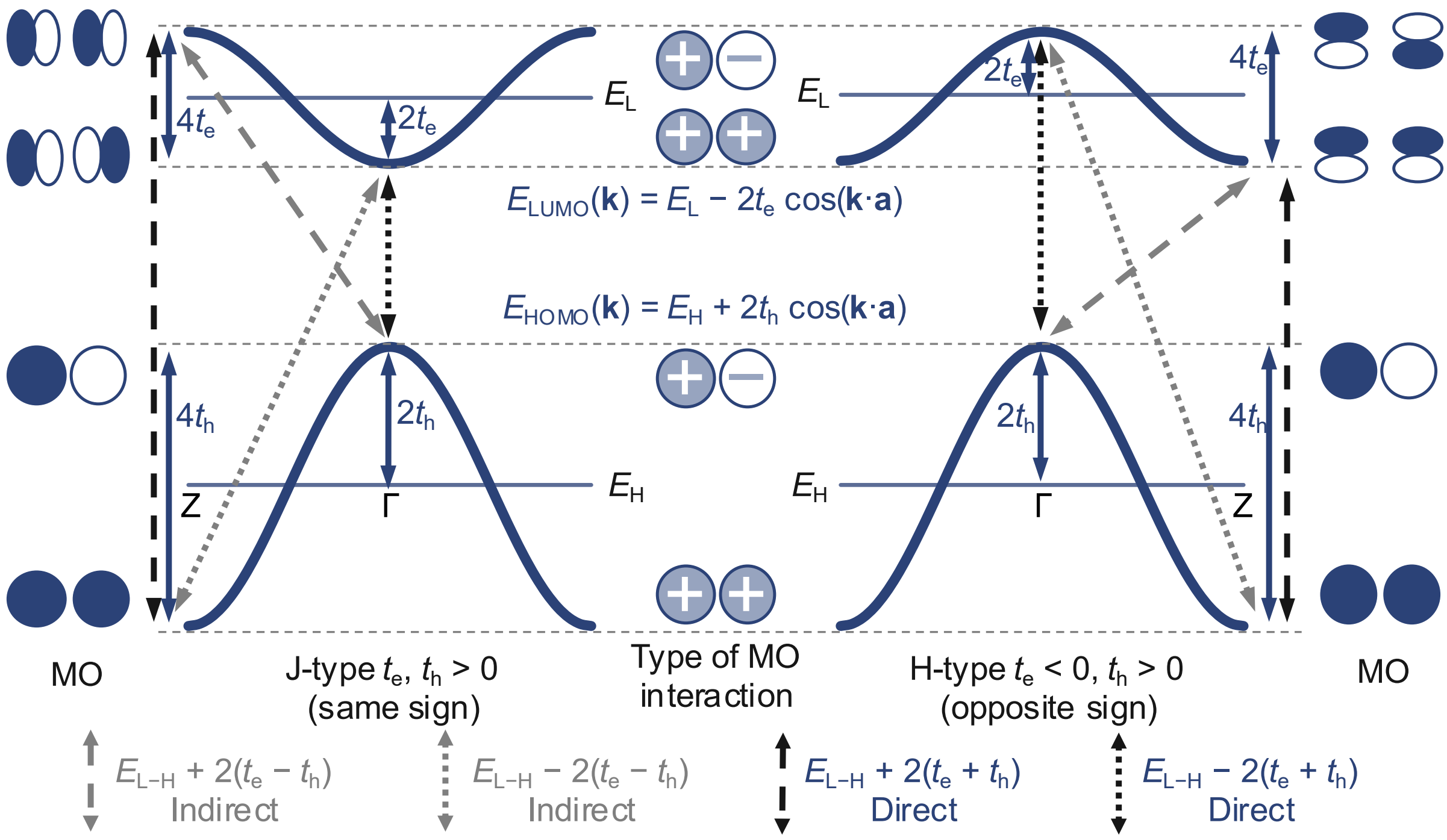

Figure S20. Schematic representation of the band-like frontier crystal orbital (FCO) contribution arising from intermolecular frontier-orbital interactions in a one-dimensional π-stack.

The type (J or H) of FCO interaction was assigned from the relative phase relationship of the interacting frontier orbitals in the crystal-derived dimer. For this purpose, dimer orbitals were calculated by DFT at the B3LYP/def2svp level of theory, and the phase pattern of the HOMO–HOMO interaction was compared with that of the LUMO–LUMO interaction. When both orbital pairs exhibit the same relative phase relationship, the interaction is classified as J-type. In contrast, when the relative phase relationship is reversed between HOMO–HOMO and LUMO–LUMO, the interaction is classified as H-type. As shown in Figure S21, the present dimer displays such a reversed phase relationship, and was therefore assigned to H-type.

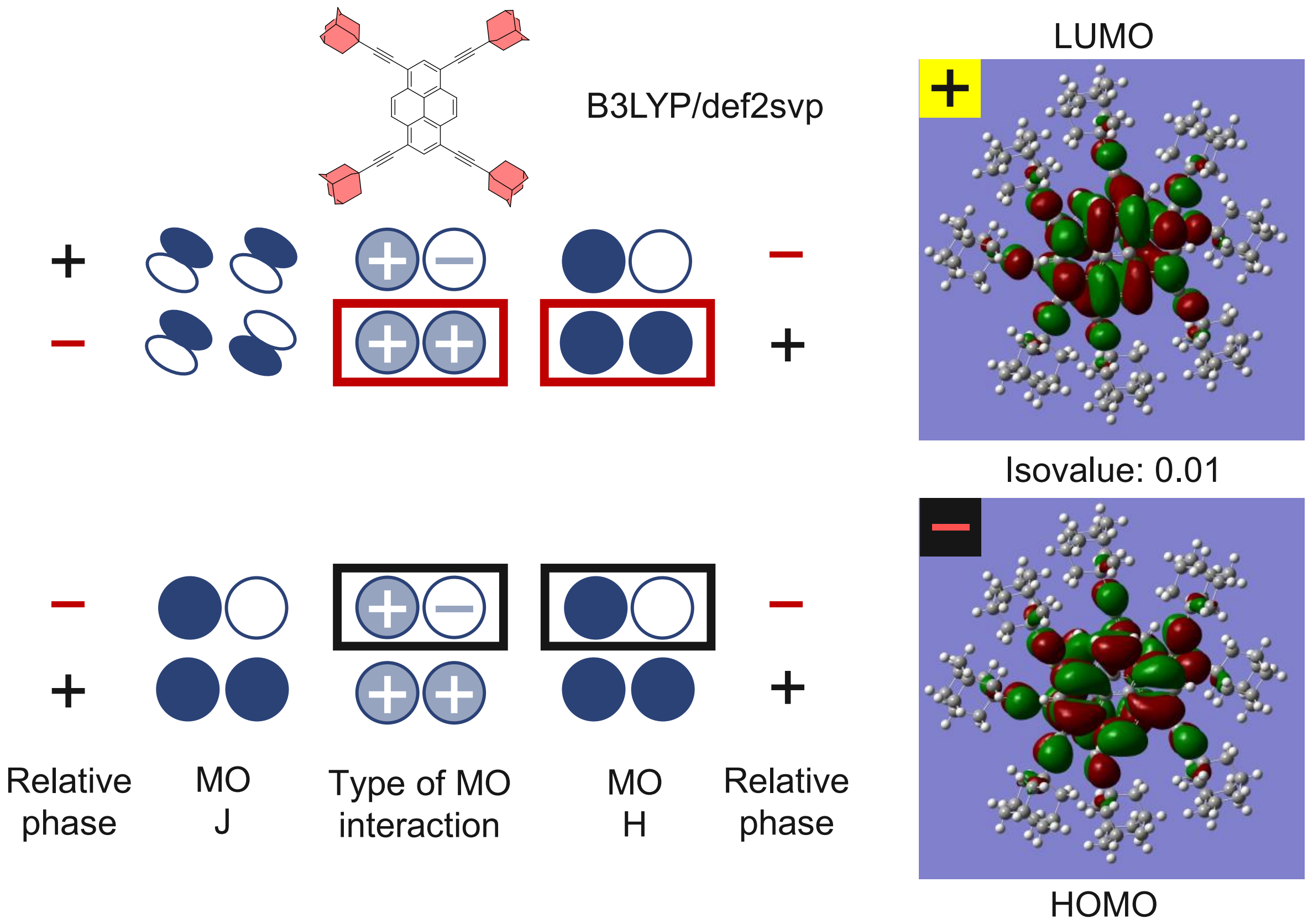

Figure S21. Assignment of J-/H-type frontier crystal orbital interaction based on the relative phase relationship of the interacting frontier orbitals in a dimer and calculated dimer frontier orbitals at the B3LYP/def2svp level of theory.

Estimation of the FCO contribution to the solution-to-crystal shift

Two models. The FCO contribution was evaluated using two different approaches: a transfer-integral model based on fragment orbital analysis and a dimer orbital-energy model based on calculation of the full dimer. The first approach estimates the degree of intermolecular orbital interaction from the calculated monomer orbitals of the individual molecules, whereas the second provides an estimate based on the frontier-orbital energies of the full dimer. In both cases, a one-dimensional tight-binding model was used to extend the effect to an ideal polymeric system. Comparison of the two models makes it possible to estimate the effect of orbital renormalization upon dimer formation, which is not captured in the first approach because of its fragment-based nature but is included in the second. The second approach was therefore adopted for the final evaluation.

Transfer-integral model. The transfer integrals were calculated within a fragment-analysis framework using the Amsterdam Density Functional (ADF) package.[S20] Within this model, the FCO-based change

in the frontier-orbital gap is estimated from the hole and electron transfer integrals, t_h and t_e, according to

$$\Delta E_{\mathrm{FCO}}^{\mathrm{frag}} = -2(|t_{\mathrm{h}}| + |t_{\mathrm{e}}|)$$

The factor of 2 reflects the extension from a single nearest-neighbor interaction to an infinite one-dimensional stack.

Table S11. Temperature-dependent hole and electron transfer integrals, t_h and t_e, and the resulting fragment-based FCO contribution, $\Delta E_{\mathrm{FCO}}^{\mathrm{frag}}$, for the polymeric π-stacks of 1 in CP1.[a]

Temperature	t_h	t_e	$\Delta E_{\mathrm{FCO}}^{\mathrm{frag}}$
K	eV	eV	eV
**90**	+0.163	−0.231	−0.788
**100**	+0.157	−0.232	−0.777
**120**	+0.148	−0.227	−0.750
**140**	+0.146	−0.226	−0.743
**160**	+0.142	−0.222	−0.729
**180**	+0.140	−0.220	−0.720
**200**	+0.139	−0.220	−0.718
**220**	+0.133	−0.216	−0.698
**240**	+0.132	−0.214	−0.694
**260**	+0.128	−0.207	−0.669
**280**	+0.124	−0.204	−0.656
**298**	+0.116	−0.199	−0.630

[a] t_h, t_e: hole and electron transfer integral obtained for the crystal-derived dimer geometry. $\Delta E_{\mathrm{FCO}}^{\mathrm{frag}}$: fragment-based FCO contribution for the polymeric one-dimensional stack, calculated as $\Delta E_{\mathrm{FCO}}^{\mathrm{frag}} = -2(|t_h| + |t_e|)$ within a nearest-neighbor tight-binding approximation. Negative values of $\Delta E_{\mathrm{FCO}}^{\mathrm{frag}}$ correspond to narrowing of the frontier-orbital gap and thus to a red-shifting FCO contribution.

Dimer orbital-energy model.[S3] As an alternative estimate, the FCO contribution was evaluated from the change in the frontier-orbital energy gap upon dimer formation. The HOMO–LUMO gaps of the monomer and the crystal-derived dimer were defined as

$$E_{\mathrm{g}}^{\mathrm{1mer}} = E_{\mathrm{LUMO}}^{\mathrm{1mer}} - E_{\mathrm{HOMO}}^{\mathrm{1mer}}$$

and

$$E_{\mathrm{g}}^{\mathrm{2mer}} = E_{\mathrm{LUMO}}^{\mathrm{2mer}} - E_{\mathrm{HOMO}}^{\mathrm{2mer}}$$

, respectively, where $E_{\mathrm{HOMO}}^{\mathrm{1mer}}$ and $E_{\mathrm{LUMO}}^{\mathrm{1mer}}$ are the HOMO and LUMO energies of the monomer, and $E_{\mathrm{HOMO}}^{\mathrm{2mer}}$ and $E_{\mathrm{LUMO}}^{\mathrm{2mer}}$ are the corresponding dimer frontier-orbital energies. The corresponding FCO contribution was then approximated as

$$\Delta E_{\mathrm{FCO}} = 2(E_{\mathrm{g}}^{\mathrm{2mer}} - E_{\mathrm{g}}^{\mathrm{1mer}})$$

. Here, ΔE_{FCO} denotes the frontier-crystal-orbital contribution to the optical energy shift estimated from dimer-induced frontier-orbital gap narrowing. Again, the factor of 2 accounts for extension from the dimer, which contains one intermolecular interaction, to the polymeric one-dimensional stack, in which each molecule interacts with two neighboring molecules along the stacking direction.

Table S12. Temperature-dependent monomer and crystal-derived dimer frontier-orbital energies and the resulting dimer-based FCO contribution, Δ*E*FCO, for the polymeric π-stacks of 1 in CP1.[a]

T	$E_{\mathrm{HOMO}}^{\mathrm{1mer}}$	$E_{\mathrm{LUMO}}^{\mathrm{1mer}}$	$E_{\mathrm{g}}^{\mathrm{1mer}}$	$E_{\mathrm{HOMO}}^{\mathrm{2mer}}$	$E_{\mathrm{LUMO}}^{\mathrm{2mer}}$	$E_{\mathrm{g}}^{\mathrm{2mer}}$	ΔE_{FCO}
K	eV	eV	eV	eV	eV	eV	eV
**90**	−5.058	−2.016	3.042	−4.665	−2.055	2.610	−0.864
**100**	−5.047	−2.013	3.034	−4.648	−2.072	2.575	−0.918
**120**	−5.052	−2.023	3.029	−4.661	−2.064	2.597	−0.865
**140**	−5.067	−2.018	3.049	−4.673	−2.064	2.609	−0.879
**160**	−5.052	−2.037	3.015	−4.673	−2.060	2.613	−0.803
**180**	−5.039	−2.053	2.986	−4.662	−2.070	2.592	−0.787
**200**	−5.070	−2.021	3.049	−4.700	−2.062	2.638	−0.822
**220**	−5.079	−2.007	3.071	−4.711	−2.032	2.679	−0.785
**240**	−5.063	−2.010	3.053	−4.702	−2.025	2.677	−0.753
**260**	−5.060	−2.027	3.033	−4.701	−2.038	2.664	−0.739
**280**	−5.086	−1.983	3.103	−4.710	−2.005	2.705	−0.796
**298**	−5.115	−1.982	3.133	−4.756	−1.988	2.768	−0.730

[a] T: temperature (K). $E_{\mathrm{HOMO}}^{\mathrm{1mer}}$ and $E_{\mathrm{LUMO}}^{\mathrm{1mer}}$: HOMO and LUMO energies of the crystal-derived monomer. $E_{\mathrm{g}}^{\mathrm{1mer}}$: monomer HOMO-LUMO gap, calculated as $E_{\mathrm{g}}^{\mathrm{1mer}} = E_{\mathrm{LUMO}}^{\mathrm{1mer}} - E_{\mathrm{HOMO}}^{\mathrm{1mer}}$. $E_{\mathrm{HOMO}}^{\mathrm{2mer}}$ and $E_{\mathrm{LUMO}}^{\mathrm{2mer}}$: HOMO and LUMO energies of the crystal-derived dimer. $E_{\mathrm{g}}^{\mathrm{2mer}}$: dimer HOMO-LUMO gap, calculated as $E_{\mathrm{g}}^{\mathrm{2mer}} = E_{\mathrm{LUMO}}^{\mathrm{2mer}} - E_{\mathrm{HOMO}}^{\mathrm{2mer}}$. ΔE_{FCO}: dimer-based FCO contribution for the polymeric one-dimensional stack, calculated as $\Delta E_{\mathrm{FCO}} = 2(E_{\mathrm{g}}^{\mathrm{2mer}} - E_{\mathrm{g}}^{\mathrm{1mer}})$. Negative values of ΔE_{FCO} correspond to narrowing of the frontier-orbital gap and thus to a red-shifting FCO contribution.

Difference between the models. The transfer-integral model describes the band-gap change only in terms of fragment-based intermolecular couplings between the monomer frontier orbitals. As such, it captures the energetic effect of orbital splitting, but does not include additional stabilization or destabilization arising from mutual orbital reorganization upon dimer formation. In contrast, the dimer orbital-energy model is based on the total frontier-orbital energies of the interacting dimer and therefore contains both the simple splitting contribution and these additional reorganization effects. The difference between the two estimates may thus be expressed as

$$\Delta E_{\mathrm{reorg}} = \Delta E_{\mathrm{FCO}} - \Delta E_{\mathrm{FCO}}^{\mathrm{frag}}$$

where $\Delta E_{\mathrm{reorg}}$ represents the part of the FCO stabilization not captured by the fragment-based transfer-integral picture. In the present context, this additional term is interpreted primarily as arising from orbital reorganization in the interacting dimer.

Table S13. Temperature-dependent fragment-based and dimer-based FCO contributions and the resulting reorganization contribution, $\Delta E_{\mathrm{reorg}}$, for the polymeric π-stacks of 1 in CP1.[a]

Temperature	$\Delta E_{\mathrm{FCO}}^{\mathrm{frag}}$	ΔE_{FCO}	$\Delta E_{\mathrm{reorg}}$
K	eV	eV	eV
**90**	−0.788	−0.864	−0.076
**100**	−0.777	−0.918	−0.141
**120**	−0.750	−0.865	−0.115
**140**	−0.743	−0.879	−0.137
**160**	−0.729	−0.803	−0.074
**180**	−0.720	−0.787	−0.067
**200**	−0.718	−0.822	−0.104
**220**	−0.698	−0.785	−0.087
**240**	−0.694	−0.753	−0.060
**260**	−0.669	−0.739	−0.070
**280**	−0.656	−0.796	−0.140
**298**	−0.630	−0.730	−0.100

[a] $\Delta E_{\mathrm{FCO}}^{\mathrm{frag}}$: fragment-based FCO contribution calculated by charge transfer integrals for the polymeric one-dimensional stack, calculated from $\Delta E_{\mathrm{FCO}}^{\mathrm{frag}} = -2(|t_{\mathrm{h}}| + |t_{\mathrm{e}}|)$. ΔE_{FCO}: dimer-based FCO contribution, calculated as $\Delta E_{\mathrm{FCO}} = 2(E_{\mathrm{g}}^{\mathrm{2mer}} - E_{\mathrm{g}}^{\mathrm{1mer}})$. $\Delta E_{\mathrm{reorg}}$: additional contribution not captured by the fragment-based transfer-integral model, defined as $\Delta E_{\mathrm{reorg}} = \Delta E_{\mathrm{FCO}} - \Delta E_{\mathrm{FCO}}^{\mathrm{frag}}$. Negative values of $\Delta E_{\mathrm{reorg}}$ indicate additional gap narrowing beyond the simple fragment-based orbital-splitting picture and are attributed here to orbital reorganization effects in the interacting dimer.

As summarized in Table S13, the fragment-based FCO contribution, $\Delta E_{\mathrm{FCO}}^{\mathrm{frag}}$, is consistently smaller in magnitude than the dimer-based estimate, ΔE_{FCO}. This is expected because $\Delta E_{\mathrm{FCO}}^{\mathrm{frag}}$ is derived solely from the transfer integrals within the fragment-based tight-binding picture and therefore accounts only for the frontier-orbital splitting caused by intermolecular electronic coupling. In contrast, ΔE_{FCO}, obtained from the dimer–monomer HOMO–LUMO gap difference, additionally includes energetic effects arising from mutual orbital reorganization within the interacting dimer. The relationship between these quantities is defined as $\Delta E_{\mathrm{FCO}}^{\mathrm{frag}} = -2(|t_{\mathrm{h}}| + |t_{\mathrm{e}}|)$, $\Delta E_{\mathrm{FCO}} = 2(E_{\mathrm{g}}^{\mathrm{2mer}} - E_{\mathrm{g}}^{\mathrm{1mer}})$, and $\Delta E_{\mathrm{reorg}} = \Delta E_{\mathrm{FCO}} - \Delta E_{\mathrm{FCO}}^{\mathrm{frag}}$.

The variation in ΔE_{FCO} with increasing temperature is larger than that in $\Delta E_{\mathrm{FCO}}^{\mathrm{frag}}$. We attribute this larger variation in ΔE_{FCO} to the following reason. **CP1** exhibits whole-molecule disorder: for each π-stacking monomer, two disordered molecular positions overlap with nearly 50:50 occupancies. As a result, the atomic positions show comparatively larger standard deviations than in cases without whole-molecule disorder. This larger structural uncertainty leads to greater errors in the calculated dimer frontier-orbital energies, because these also include energy deviations arising from non-optimized structural parameters. In other words, slight deviations in the atomic positions from their optimized values, including bond lengths and related structural parameters, introduce errors into the dimer HOMO–LUMO energies that are not directly related to stabilization or destabilization arising from intermolecular orbital overlap. Although stronger restraints or constraints during crystal-structure refinement, or additional geometry optimization by theoretical calculations, might produce smoother trends in ΔE_{FCO}, we chose not to do so in the present study in order to avoid overmodeling the molecular geometry of **1**.

Coherence lengths

Table S14 summarizes the experimentally observed optical shifts, ΔE_{obs}, together with the calculated FCO-based (ΔE_{FCO}) and total energy-shift (ΔE_{total}) estimates for the polymeric π-stacks of **1** in **CP1**. The comparison shows that the dimer-based FCO contribution, ΔE_{FCO}, already reproduces the correct sign, that is, a negative shift corresponding to a red shift, and accounts for a substantial fraction of the observed bathochromic shift. On this basis, an effective coherence length considering only FCO contribution $N_{\mathrm{coh}}^{\mathrm{FCO}}$ was estimated from

$$\Delta E_{\mathrm{obs}} = \Delta E_{\mathrm{FCO}} \cos\left[\frac{\pi}{N_{\mathrm{coh}}^{\mathrm{FCO}} + 1}\right]$$

giving $N_{\mathrm{coh}}^{\mathrm{FCO}}$ values in the range of about 4 to 9. Because the temperature dependence of these values is somewhat irregular, presumably due to large geometrical fluctuations arising from the heavy disorder of the crystal structure (vide supra), $N_{\mathrm{coh,fit}}^{\mathrm{FCO}}$ was additionally obtained by interpolation to provide a smooth temperature-dependent estimate. This procedure gave values consistently around 5 to 8 over the whole temperature range.

A total upper-bound red-shift estimate, ΔE_{total}, was constructed by combining ΔE_{FCO} with ΔE_{exci}, where all excitonic contributions were treated as red-shifting:

$$\Delta E_{\mathrm{total}} = \Delta E_{\mathrm{FCO}} + \Delta E_{\mathrm{exci}}$$

This treatment was chosen because formally forbidden transitions may acquire partial intensity in the crystal owing to vibronic coupling, static disorder, phonon-assisted relaxation, or other symmetry-breaking effects. The corresponding effective coherence length including all relevant contributions, N_{coh}, was then estimated from

$$\Delta E_{\mathrm{obs}} = \Delta E_{\mathrm{total}} \cos\left[\frac{\pi}{N_{\mathrm{coh}} + 1}\right]$$

The resulting N_{coh} values are slightly smaller than $N_{\mathrm{coh,fit}}^{\mathrm{FCO}}$ and are generally around 2 to 3. This is consistent with the interpretation that excitonic coupling provides only a minor secondary contribution relative to the dominant FCO-based band contribution. In this study, N_{coh} is therefore regarded as the more relevant effective coherence scale, whereas $N_{\mathrm{coh}}^{\mathrm{FCO}}$ is used primarily to assess how strongly the inclusion of excitonic contributions modifies the coherence estimate.

The comparatively low N_{coh} value of 2.5, relative to values commonly reported for exciton coherence,[S23] may be attributed to the whole-molecule disorder within the π-stack. This disorder may act as a structural perturbation that disrupts coherent orbital interactions already at the level of the third molecule in the column.

By contrast, the formally defined total shift, $\Delta E_{\mathrm{total}}^{\mathrm{formal}}$, retains the formal sign assignment and therefore treats H-type excitonic contributions as positive, that is, blue-shifting:

$$\Delta E_{\mathrm{total}}^{\mathrm{formal}} = \Delta E_{\mathrm{FCO}} + \Delta E_{\mathrm{exci}}^{\mathrm{formal}}$$

These $\Delta E_{\mathrm{total}}^{\mathrm{formal}}$ values are less red-shifted than ΔE_{FCO} alone, consistent with the small and slightly positive values of $\Delta E_{\mathrm{exci}}^{\mathrm{formal}}$. In some cases, they are even less negative than ΔE_{obs}, indicating that the available red-shift budget would be too small even under the assumption of infinite, and therefore unrealistic, coherence. This suggests that the experimentally observed crystalline-state peaks are unlikely to originate from formally allowed H-type shifted transitions. Overall, the data support the conclusion that the dominant contribution to the large observed red shift arises from band-like frontier-orbital interactions, whereas excitonic effects provide only a minor correction.

Table S14. Temperature-dependent experimental and calculated optical shifts, corresponding coherence lengths, and total energy-shift estimates for the polymeric π-stacks of 1 in CP1.[a]

T	**ΔE_{obs}**	**ΔE_{FCO}**	N_{coh}^{FCO}	$N_{coh,fit}^{FCO}$	**ΔE_{total}**	**N_{coh}**	$\Delta E_{total}^{formal}$
K	eV	eV	units	units	eV	units	eV
**77**	−0.768						
**90**	−0.766	−0.864	5.5	5.1	−1.299	2.3	−0.777
**100**	−0.761	−0.918	4.3	5.2	−1.344	2.2	−0.843
**120**	−0.758	−0.865	5.2	5.5	−1.251	2.4	−0.797
**140**	−0.754	−0.879	4.8	5.8	−1.266	2.4	−0.810
**160**	−0.748	−0.803	7.4	6.2	−1.167	2.6	−0.736
**180**	−0.738	−0.787	7.8	6.5	−1.146	2.6	−0.719
**200**	−0.731	−0.822	5.6	6.8	−1.168	2.5	−0.759
**220**	−0.728	−0.785	7.2	7.1	−1.104	2.7	−0.727
**240**	−0.714	−0.753	8.7	7.4	−1.068	2.7	−0.695
**260**	−0.703	−0.739	9.0	7.7	−1.033	2.8	−0.684
**280**	−0.698	−0.796	5.3	8.0	−1.086	2.6	−0.737
**298**	−0.691	−0.730	8.6	8.3	−0.979	3.0	−0.686

[a] *T*: temperature (K). ΔE_{obs}: experimentally observed solution-to-crystal shift. ΔE_{FCO}: dimer-based FCO contribution, calculated as $2(E_g^{2mer} - E_g^{1mer})$. N_{coh}^{FCO}: effective coherence length obtained by comparing ΔE_{obs} with ΔE_{FCO} according to $\Delta E_{obs} = \Delta E_{FCO}\cos[\pi/(N_{coh}^{FCO} + 1)]$. $N_{coh,fit}^{FCO}$: fitted effective coherence length obtained from linear interpolation of N_{coh}^{FCO} values over temperature. ΔE_{total}: total red-shifting upper-bound estimate obtained by combining ΔE_{FCO} with ΔE_{exci}, i.e., $\Delta E_{total} = \Delta E_{FCO} + \Delta E_{exci}$. N_{coh}: effective coherence length obtained by comparing ΔE_{obs} with ΔE_{total} according to $\Delta E_{obs} = \Delta E_{total}\cos[\pi/(N_{coh}+1)]$. $\Delta E_{total}^{formal}$: total shift obtained from formal summation of FCO and excitonic contributions, $\Delta E_{total}^{formal} = \Delta E_{FCO} + \Delta E_{exci}$. "units" for N_{coh} values indicates dimensionless quantities.

Effective FCO and excitonic contributions

The effective FCO and excitonic contributions were obtained by applying the coherence factor derived from N_{coh} to the ideal fully coherent contributions. In this simplified treatment, other possible factors, such as local geometrical disorder, electron–phonon coupling, and changes in dielectric screening, were not treated explicitly. These effects were assumed to be non-dominant for the present rigid, apolar crystalline phase **CP1**. The same effective coherence length was applied to both the FCO and excitonic contributions because both terms originate from intermolecular orbital overlap within the π-stack and were therefore assumed to be governed by the same coherence scale. Thus, $\Delta E_{\mathrm{FCO}}^{\mathrm{eff}}$ and $\Delta E_{\mathrm{exci}}^{\mathrm{eff}}$ represent the parts of ΔE_{FCO} and ΔE_{exci} that are effective within the finite coherent segment of the π-stack:

$$\Delta E_{\mathrm{FCO}}^{\mathrm{eff}} = \Delta E_{\mathrm{FCO}} \cos\left[\frac{\pi}{N_{\mathrm{coh}}+1}\right]$$
$$\Delta E_{\mathrm{exci}}^{\mathrm{eff}} = \Delta E_{\mathrm{exci}} \cos\left[\frac{\pi}{N_{\mathrm{coh}}+1}\right]$$

By construction, the sum of these two effective contributions reproduces the experimentally observed solution-to-crystal shift:

$$\Delta E_{\mathrm{obs}} = \Delta E_{\mathrm{FCO}}^{\mathrm{eff}} + \Delta E_{\mathrm{exci}}^{\mathrm{eff}}$$

The resulting values are summarized in Table S15. Across the measured temperature range, $\Delta E_{\mathrm{FCO}}^{\mathrm{eff}}$ remains the larger contribution, whereas $\Delta E_{\mathrm{exci}}^{\mathrm{eff}}$ provides a smaller additional red-shifting contribution. This supports the interpretation that the large solution-to-crystal shift from **1** to **CP1** is dominated by finite-coherence FCO formation, with exciton coupling acting as a secondary contribution of the shift.

Upon heating, both effective contributions $\Delta E_{\mathrm{FCO}}^{\mathrm{eff}}$ and $\Delta E_{\mathrm{exci}}^{\mathrm{eff}}$ become gradually less negative, following the reduction in the absolute magnitude of ΔE_{obs}. This trend is consistent with thermal expansion of the π-stack and the accompanying weakening of intermolecular electronic coupling. The analysis therefore indicates that the temperature dependence of the optical shift is governed mainly by changes in the strength of intermolecular coupling, while the effective coherence length remains between 2.3 and 3.0 and is largely temperature independent.

Table S15. Temperature-dependent coherence-length-scaled FCO and excitonic contributions calculated from the experimental solution-to-crystal shift and crystal-structure data.[a]

T	ΔE_{obs}	N_{coh}	ΔE_{FCO}	ΔE_{FCO}^{eff}	ΔE_{exci}	ΔE_{exci}^{eff}
K	eV	units	eV		eV	
**77**	−0.768					
**90**	−0.766	2.3	−0.864	−0.509	−0.435	−0.257
**100**	−0.761	2.2	−0.918	−0.520	−0.426	−0.241
**120**	−0.758	2.4	−0.865	−0.524	−0.386	−0.234
**140**	−0.754	2.4	−0.879	−0.524	−0.387	−0.230
**160**	−0.748	2.6	−0.803	−0.514	−0.365	−0.233
**180**	−0.738	2.6	−0.787	−0.507	−0.358	−0.231
**200**	−0.731	2.5	−0.822	−0.514	−0.347	−0.217
**220**	−0.728	2.7	−0.785	−0.518	−0.319	−0.210
**240**	−0.714	2.7	−0.753	−0.504	−0.315	−0.210
**260**	−0.703	2.8	−0.739	−0.503	−0.294	−0.200
**280**	−0.698	2.6	−0.796	−0.511	−0.290	−0.186
**298**	−0.691	3.0	−0.730	−0.515	−0.250	−0.176

a T: temperature (K). ΔE_{obs}: experimentally observed solution-to-crystal shift. N_{coh}: effective coherence length obtained by comparing ΔE_{obs} with the total red-shifting upper-bound estimate according to $\Delta E_{obs} = (\Delta E_{FCO} + \Delta E_{exci}) \cos[\pi/(N_{coh} + 1)]$. ΔE_{FCO}: ideal fully coherent FCO contribution, equivalent to the ideal band-structure contribution for an infinite π-stack. ΔE_{FCO}^{eff}: finite-coherence-scaled FCO contribution, calculated as $\Delta E_{FCO}^{eff} = \Delta E_{FCO} \cos[\pi/(N_{coh} + 1)]$. ΔE_{exci}: upper-bound excitonic contribution including Coulombic and CT-mediated terms. ΔE_{exci}^{eff}: finite-coherence-scaled excitonic contribution, calculated as $\Delta E_{exci}^{eff} = \Delta E_{exci} \cos[\pi/(N_{coh} + 1)]$. "units" for N_{coh} indicates a dimensionless quantity. Values at 77 K were not decomposed because the corresponding structural parameters were not available.

Table S16. Summary of temperature-dependent structural parameters, electronic couplings, energetic contributions, and coherence lengths for the π-stacked crystal of 1 in CP1.[a]

T	b	$d_{\pi\text{-}\pi}$	ΔE_{obs}	t_h	t_e	ΔE^{frag}_{FCO}	ΔE_{FCO}	N^{FCO}_{coh}	$N^{FCO}_{coh,fit}$	$E_{CT}−E_{S1}$	J_{Coul}	J_{CT}	Δ_{CT}	ΔE_{exci}	ΔE_{total}	N_{coh}	ΔE^{formal}_{exci}	$\Delta E^{formal}_{total}$
K	Å	Å	eV	eV	eV	eV	eV	units	units	eV	eV	eV	eV	eV	eV	units	eV	eV
**77**			−0.768															
**90**	6.591	3.296	−0.766	+0.163	−0.231	−0.788	−0.864	5.5	5.1	0.916	+0.048	+0.082	−0.174	−0.435	−1.299	2.3	+0.086	−0.777
**100**	6.594	3.297	−0.761	+0.157	−0.232	−0.777	−0.918	4.3	5.2	0.894	+0.044	+0.081	−0.175	−0.426	−1.344	2.2	+0.075	−0.843
**120**	6.608	3.304	−0.758	+0.148	−0.227	−0.750	−0.865	5.2	5.5	0.919	+0.040	+0.073	−0.159	−0.386	−1.251	2.4	+0.067	−0.797
**140**	6.624	3.312	−0.754	+0.146	−0.226	−0.743	−0.879	4.8	5.8	0.909	+0.042	+0.072	−0.159	−0.387	−1.266	2.4	+0.070	−0.810
**160**	6.638	3.319	−0.748	+0.142	−0.222	−0.729	−0.803	7.4	6.2	0.936	+0.040	+0.067	−0.149	−0.365	−1.167	2.6	+0.067	−0.736
**180**	6.653	3.327	−0.738	+0.140	−0.220	−0.720	−0.787	7.8	6.5	0.938	+0.041	+0.066	−0.145	−0.358	−1.146	2.6	+0.068	−0.719
**200**	6.665	3.333	−0.731	+0.139	−0.220	−0.718	−0.822	5.6	6.8	0.954	+0.038	+0.064	−0.142	−0.347	−1.168	2.5	+0.063	−0.759
**220**	6.681	3.341	−0.728	+0.133	−0.216	−0.698	−0.785	7.2	7.1	0.987	+0.036	+0.058	−0.131	−0.319	−1.104	2.7	+0.058	−0.727
**240**	6.696	3.348	−0.714	+0.132	−0.214	−0.694	−0.753	8.7	7.4	0.989	+0.036	+0.057	−0.128	−0.315	−1.068	2.7	+0.058	−0.695
**260**	6.714	3.357	−0.703	+0.128	−0.207	−0.669	−0.739	9.0	7.7	0.988	+0.034	+0.053	−0.119	−0.294	−1.033	2.8	+0.055	−0.684
**280**	6.734	3.367	−0.698	+0.124	−0.204	−0.656	−0.796	5.3	8.0	0.986	+0.036	+0.051	−0.116	−0.290	−1.086	2.6	+0.059	−0.737
**298**	6.751	3.376	−0.691	+0.116	−0.199	−0.630	−0.730	8.6	8.3	1.031	+0.029	+0.045	−0.103	−0.250	−0.979	3.0	+0.044	−0.686

[a] T: temperature (K). b: crystallographic b-axis length; $d_{\pi-\pi}$: intermolecular π–π distance, defined as $d_{\pi-\pi} = b/2$; ΔE_{obs}: experimentally observed solution-to-crystal shift; t_h and t_e: hole and electron transfer integrals; ΔE^{frag}_{FCO}: fragment-based FCO contribution from transfer integrals; ΔE_{FCO}: FCO contribution from dimer frontier-orbital splitting; N^{FCO}_{coh}: effective coherence length estimated from $\Delta E_{obs} = \Delta E_{FCO}\cos[\pi/(N^{FCO}_{coh}+1)]$; $N^{FCO}_{coh,fit}$: fitted effective coherence length from the FCO-only model; $E_{CT} − E_{S1}$: energy difference between the charge-transfer and locally excited states; J_{Coul}:Coulombic excitonic coupling; J_{CT}: charge-transfer-mediated excitonic coupling; Δ_{CT}: CT-induced site-energy correction; ΔE_{exci}: Upper red-shift limit of CT-mediated and Coulombic excitonic interactions, including the site-energy correction Δ_{CT}, defined as $\Delta E_{exci} = \Delta_{CT} −2(|J_{CT}|+|J_{Coul}|)$; ΔE_{total}: total red-shifting upper-bound estimate obtained by combining ΔE_{FCO} with ΔE_{exci}, i.e., $\Delta E_{total} = \Delta E_{FCO} + \Delta E_{exci}$; N_{coh}: effective coherence length obtained by comparing ΔE_{obs} with ΔE_{total} according to $\Delta E_{obs} = \Delta E_{total}\cos[\pi/(N_{coh}+1)]$; ΔE^{formal}_{exci}: Overall CT-mediated and Coulombic excitonic contribution including the site-energy correction Δ_{CT}, with signs preserved (allowed transitions), defined as $\Delta E^{formal}_{exci} = \Delta_{CT} +2(J_{CT} + J_{Coul})$; $\Delta E^{formal}_{total}$: total shift obtained from formal summation of FCO and excitonic contributions, $\Delta E^{formal}_{total} = \Delta E_{FCO} + \Delta E_{exci}$. No values are listed for 77 K where the corresponding parameters were not available.

8. Polarization optical microscopy (POM)

Polarized optical microscopy (POM) was used to examine the optical anisotropy and external habit of the crystals. Sample crystal was placed on a glass slide for measurement. Representative crystals displayed a strongly elongated, needle-like morphology. Upon changing the orientation of the polarizer/analyzer relative to the crystal long axis, pronounced variations in brightness were observed in both fluorescence and transmitted-light images, indicating marked polarization dependence of the optical response and thus a well-defined anisotropic crystalline order.

To relate the observed crystal habit to the crystal structure, a BFDH morphology was generated from the single-crystal X-ray diffraction data. The calculated morphology reproduces the strongly elongated habit of the crystals and indicates preferential growth along the crystallographic *a*-axis. The agreement between experiment and the BFDH model supports the crystallographic assignment of the dominant growth direction and is consistent with the uniform, well-developed morphology observed by optical microscopy.

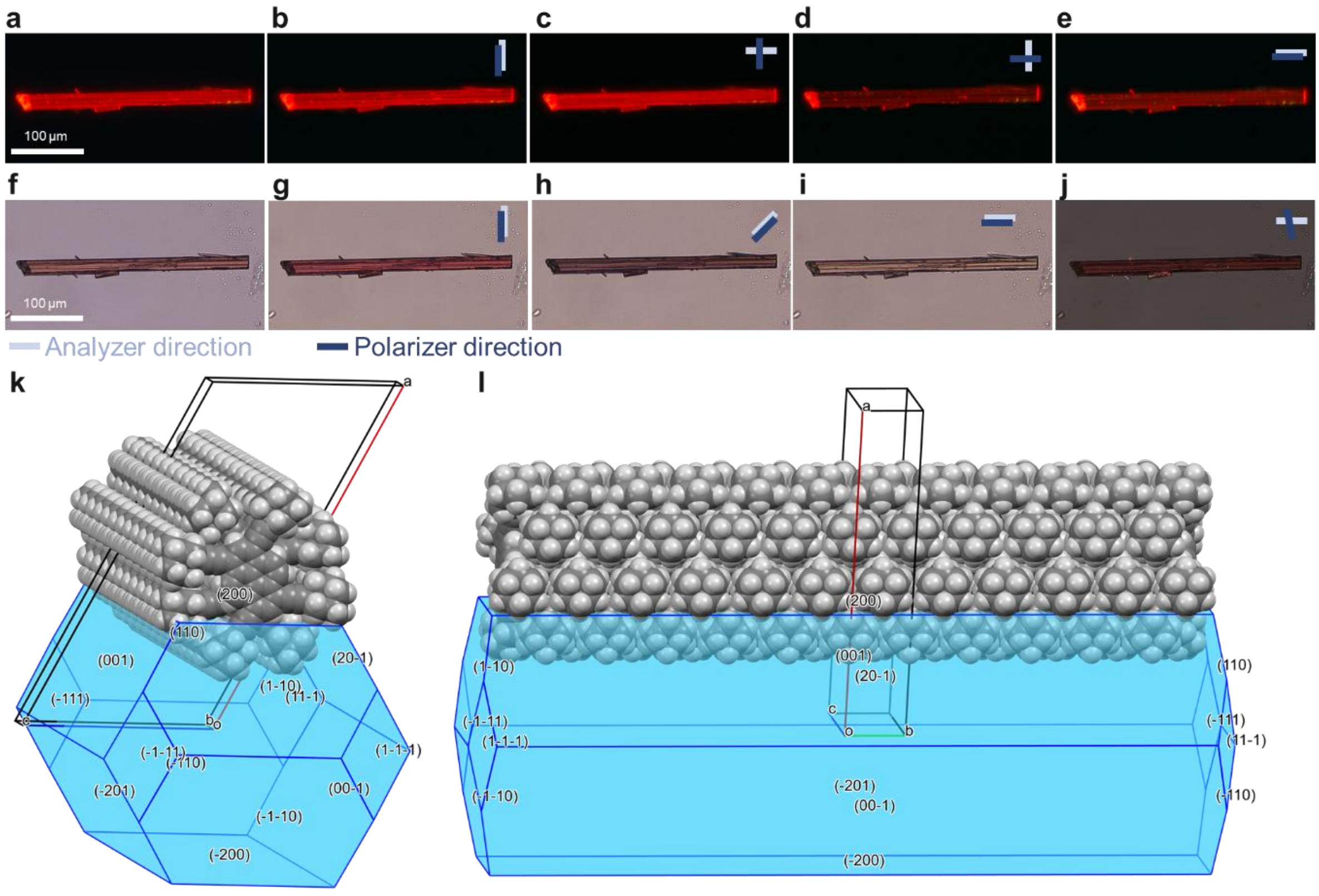

Figure S22. Polarized optical microscopy (POM) images of a representative crystal recorded under different orientations of the polarizer/analyzer relative to the crystal long axis. (a–e) Fluorescence POM images and (f–j) corresponding bright-field/POM images obtained for different polarization directions, as indicated by the blue markers. The analyzer and polarizer directions are summarized in the inset. (k,l) BFDH morphology calculated from the single-crystal structure, shown in two viewing directions. The predicted morphology reproduces the strongly elongated crystal habit and indicates growth predominantly along the crystallographic *a*-axis.

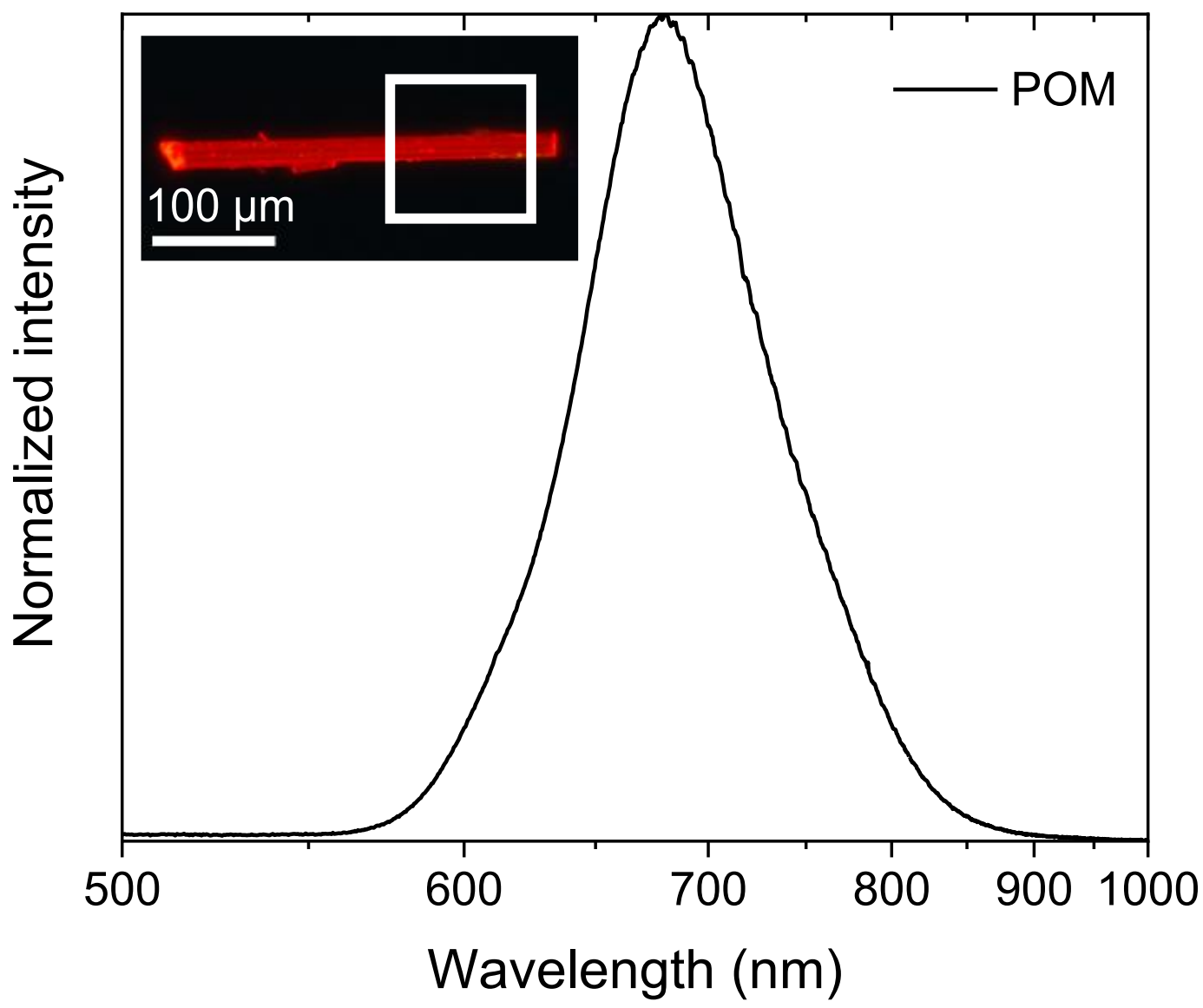

Figure S23. Emission profile of a single crystalline sample of **1** by POM. Neither polarizer nor analyzer was used. Inset shows approximate area for sampling of emission profile.

[S1] M. J. Frisch, G. W. Trucks, H. B. Schlegel, G. E. Scuseria, M. A. Robb, J. R. Cheeseman, G. Scalmani, V. Barone, G. A. Petersson, H. Nakatsuji, X. Li, M. Caricato, A. V. Marenich, J. Bloino, B. G. Janesko, R. Gomperts, B. Mennucci, H. P. Hratchian, J. V. Ortiz, A. F. Izmaylov, J. L. Sonnenberg, D. Williams-Young, F. Ding, F. Lipparini, F. Egidi, J. Goings, B. Peng, A. Petrone, T. Henderson, D. Ranasinghe, V. G. Zakrzewski, J. Gao, N. Rega, G. Zheng, W. Liang, M. Hada, M. Ehara, K. Toyota, R. Fukuda, J. Hasegawa, M. Ishida, T. Nakajima, Y. Honda, O. Kitao, H. Nakai, T. Vreven, K. Throssell, J. A. Montgomery, Jr., J. E. Peralta, F. Ogliaro, M. J. Bearpark, J. J. Heyd, E. N. Brothers, K. N. Kudin, V. N. Staroverov, T. A. Keith, R. Kobayashi, J. Normand, K. Raghavachari, A. P. Rendell, J. C. Burant, S. S. Iyengar, J. Tomasi, M. Cossi, J. M. Millam, M. Klene, C. Adamo, R. Cammi, J. W. Ochterski, R. L. Martin, K. Morokuma, O. Farkas, J. B. Foresman, D. J. Fox, *Gaussian 16*, Revision A.03, Gaussian, Inc., Wallingford CT, 2016.

[S2] R. Dennington, T. A. Keith, J. M. Millam, *GaussView*, Version 6, Semichem Inc., Shawnee Mission, KS, 2016.

[S3] B. Herbert, K. Shoyama, "Interplay of Orbital Overlap and Exciton Coupling in the Optical Properties of Adamantylethynyl-Substituted Pyrene Molecular Crystals", *Chemistry – A European Journal* (2026): e70949.

[S4] W. Kabsch, "XDS", *Acta Crystallographica Section D* **66**, (2010): 125–132.

[S5] G. M. Sheldrick, "SHELXT – Integrated Space-Group and Crystal-Structure Determination", *Acta Crystallographica Section A* **71**, (2015): 3–8.

[S6] G. M. Sheldrick, "Crystal Structure Refinement with SHELXL", *Acta Crystallographica Section C* **71**, (2015): 3–8.

[S7] C. B. Hübschle, G. M. Sheldrick, B. Dittrich, "ShelXle: A Qt Graphical User Interface for SHELXL", *Journal of Applied Crystallography* **44**, (2011): 1281–1284.

[S8] F. H. Allen, O. Kennard, D. G. Watson, L. Brammer, A. G. Orpen, R. Taylor, "Tables of Bond Lengths Determined by X-ray and Neutron Diffraction. Part 1. Bond Lengths in Organic Compounds", *Journal of the Chemical Society, Perkin Transactions 2* (1987): S1–S19.

[S9] A. L. Spek, "checkCIF Validation ALERTS: What They Mean and How to Respond", *Acta Crystallographica Section E* **76**, (2020): 1–11.

[S10] A. L. Spek, "Single-Crystal Structure Validation with the Program PLATON", *Journal of Applied Crystallography* **36**, (2003): 7–13.

[S11] P. L. Walker, H. A. McKinstry, C. C. Wright, "X-Ray Diffraction Studies of a Graphitized Carbon—Changes in Interlayer Spacing and Binding Energy with Temperature", *Industrial & Engineering Chemistry* **45**, (1953): 1711–1715.

[S12] K. D. Litasov, P. N. Gavryushkin, A. S. Yunoshev, S. V. Rashchenko, T. M. Inerbaev, A. T. Akilbekov, "Thermal Expansion of Coronene $C_{24}H_{12}$ at 185–416 K", *Journal of Thermal Analysis and Calorimetry* **119**, (2015): 1183–1189.
[S13] P. R. Horn, Y. Mao, M. Head-Gordon, "Probing Non-Covalent Interactions with a Second Generation Energy Decomposition Analysis Using Absolutely Localized Molecular Orbitals", *Physical Chemistry Chemical Physics* **18**, (2016): 23067–23079.
[S14] Y. Shao, Z. Gan, E. Epifanovsky, A. T. B. Gilbert, M. Wormit, J. Kussmann, A. W. Lange, A. Behn, J. Deng, X. Feng, D. Ghosh, M. Goldey, P. R. Horn, L. D. Jacobson, I. Kaliman, R. Z. Khaliullin, T. Kuś, A. Landau, J. Liu, E. I. Proynov, Y. M. Rhee, R. M. Richard, M. A. Rohrdanz, R. P. Steele, E. J. Sundstrom, H. L. Woodcock, P. M. Zimmerman, D. Zuev, B. Albrecht, E. Alguire, B. Austin, G. J. O. Beran, Y. A. Bernard, E. Berquist, K. Brandhorst, K. B. Bravaya, S. T. Brown, D. Casanova, C.-M. Chang, Y. Chen, S. H. Chien, K. D. Closser, D. L. Crittenden, M. Diedenhofen, R. A. DiStasio, H. Do, A. D. Dutoi, R. G. Edgar, S. Fatehi, L. Fusti-Molnar, A. Ghysels, A. Golubeva-Zadorozhnaya, J. Gomes, M. W. D. Hanson-Heine, P. H. P. Harbach, A. W. Hauser, E. G. Hohenstein, Z. C. Holden, T.-C. Jagau, H. Ji, B. Kaduk, K. Khistyaev, J. Kim, J. Kim, R. A. King, P. Klunzinger, D. Kosenkov, T. Kowalczyk, C. M. Krauter, K. U. Lao, A. D. Laurent, K. V. Lawler, S. V. Levchenko, C. Y. Lin, F. Liu, E. Livshits, R. C. Lochan, A. Luenser, P. Manohar, S. F. Manzer, S.-P. Mao, N. Mardirossian, A. V. Marenich, S. A. Maurer, N. J. Mayhall, E. Neuscamman, C. M. Oana, R. Olivares-Amaya, D. P. O'Neill, J. A. Parkhill, T. M. Perrine, R. Peverati, A. Prociuk, D. R. Rehn, E. Rosta, N. J. Russ, S. M. Sharada, S. Sharma, D. W. Small, A. Sodt, T. Stein, D. Stück, Y.-C. Su, A. J. W. Thom, T. Tsuchimochi, V. Vanovschi, L. Vogt, O. Vydrov, T. Wang, M. A. Watson, J. Wenzel, A. White, C. F. Williams, J. Yang, S. Yeganeh, S. R. Yost, Z.-Q. You, I. Y. Zhang, X. Zhang, Y. Zhao, B. R. Brooks, G. K. L. Chan, D. M. Chipman, C. J. Cramer, W. A. Goddard, M. S. Gordon, W. J. Hehre, A. Klamt, H. F. Schaefer, M. W. Schmidt, C. D. Sherrill, D. G. Truhlar, A. Warshel, X. Xu, A. Aspuru-Guzik, R. Baer, A. T. Bell, N. A. Besley, J.-D. Chai, A. Dreuw, B. D. Dunietz, T. R. Furlani, S. R. Gwaltney, Cs.-P. Hsu, Y. Jung, J. Kong, D. S. Lambrecht, W. Liang, C. Ochsenfeld, V. A. Rassolov, L. V. Slipchenko, J. E. Subotnik, T. Van Voorhis, J. M. Herbert, A. I. Krylov, P. M. W. Gill, M. Head-Gordon, "Advances in Molecular Quantum Chemistry Contained in the Q-Chem 4 Program Package", *Molecular Physics* **113**, (2015): 184–215.
[S15] X. Song, H. Liu, S. Liu, T. Li, L. Lv, B. Cui, T. Wang, W. Chen, Y. Chen, X. Li, "Enhancing Triplet-Triplet Annihilation Upconversion of Pyrene Derivatives for Photoredox Catalysis via Molecular Engineering" *Chem. Eur. J.* **2024**, *30*, e202302520.
[S 16] M. E. Madjet, A. Abdurahman, T. Renger, "Intermolecular Coulomb Couplings from Ab Initio Electrostatic Potentials: Application to Optical Transitions of Strongly Coupled Pigments in Photosynthetic Antennae and Reaction Centers", *Journal of Physical Chemistry B* **110**, (2006): 17268–17281.
[S 17] T. Lu, F. Chen, "Multiwfn: A Multifunctional Wavefunction Analyzer", *Journal of Computational Chemistry* **33**, (2012): 580–592.
[S18] C. Friedl, D. G. Fedorov, T. Renger, "Towards a Quantitative Description of Excitonic Couplings in Photosynthetic Pigment–Protein Complexes: Quantum Chemistry Driven Multiscale Approaches", *Physical Chemistry Chemical Physics* **24**, (2022): 5014–5038.
[S19] N. J. Hestand, F. C. Spano, "Expanded Theory of H- and J-Molecular Aggregates: The Effects of Vibronic Coupling and Intermolecular Charge Transfer", *Chemical Reviews* **118**, (2018): 7069–7163.
[S20] E. J. Baerends, N. F. Aguirre, N. D. Austin, J. Autschbach, F. M. Bickelhaupt, R. Bulo, C. Cappelli, A. C. T. van Duin, F. Egidi, C. F. Guerra, A. Förster, M. Franchini, T. P. M. Goumans, T. Heine, M. Hellström, C. R. Jacob, L. Jensen, M. Krykunov, E. van Lenthe, A. Michalak, M. M. Mitoraj, J. Neugebauer, V. P. Nicu, P. Philipsen, H. Ramanantoanina, R. Rüger, G. Schreckenbach, M. Stener, M. Swart, J. M. Thijssen, T. Trnka, L. Visscher, A. Yakovlev, S. van Gisbergen, "The Amsterdam Modeling Suite", *Journal of Chemical Physics* **162**, (2025): 162501.
[S 21] L. Gisslén, R. Scholz, "Crystallochromy of Perylene Pigments: Interference between Frenkel Excitons and Charge-Transfer States", *Physical Review B* **80**, (2009): 115309.
[S 22] R. Hoffmann, "How Chemistry and Physics Meet in the Solid State", *Angewandte Chemie International Edition* **26**, (1987): 846–878.
[S23] A. Jumbo-Nogales, V. Krivenkov, K. Rusakov, A. S. Urban, M. Grzelczak, Y. P. Rakovich, "Cross Determination of Exciton Coherence Length in J Aggregates", *Journal of Physical Chemistry Letters* **13**, (2022): 10198–10206.
